# Eya-controlled affinity between cell lineages drives tissue self-organization during *Drosophila* oogenesis

**DOI:** 10.1038/s41467-022-33845-1

**Published:** 2022-10-26

**Authors:** Vanessa Weichselberger, Patrick Dondl, Anne-Kathrin Classen

**Affiliations:** 1 Hilde-Mangold-Haus, University of Freiburg, Freiburg, Germany; 2 Faculty of Biology, University of Freiburg, Freiburg, Germany; 3 Spemann Graduate School of Biology and Medicine (SGBM), University of Freiburg, Freiburg, Germany; 4 Department for Applied Mathematics, University of Freiburg, Freiburg, Germany; 5 Cluster of Excellence livMatS, University of Freiburg, Freiburg, Germany; 6 Signalling Research Centers BIOSS and CIBSS, University of Freiburg, Freiburg, Germany

**Keywords:** Oogenesis, Morphogenesis, Cell adhesion, Stem-cell niche

## Abstract

Cooperative morphogenesis of cell lineages underlies the development of functional units and organs. To study mechanisms driving the coordination of lineages, we investigated soma-germline interactions during oogenesis. From invertebrates to vertebrates, oocytes develop as part of a germline cyst that consists of the oocyte itself and so-called nurse cells, which feed the oocyte and are eventually removed. The enveloping somatic cells specialize to facilitate either oocyte maturation or nurse cell removal, which makes it essential to establish the right match between germline and somatic cells. We uncover that the transcriptional regulator Eya, expressed in the somatic lineage, controls bilateral cell–cell affinity between germline and somatic cells in *Drosophila* oogenesis. Employing functional studies and mathematical modelling, we show that differential affinity and the resulting forces drive somatic cell redistribution over the germline surface and control oocyte growth to match oocyte and nurse cells with their respective somatic cells. Thus, our data demonstrate that differential affinity between cell lineages is sufficient to drive the complex assembly of inter-lineage functional units and underlies tissue self-organization during *Drosophila* oogenesis.

## Abstract (web summary)

Oogenesis depends on close interaction between germ cells and the surrounding somatic niche. Here the authors demonstrate that Eya controls bilateral affinity at the germline-soma interface to generate self-organizing inter-lineage units that ensure oocyte maturation.

## Introduction

Throughout development, it is essential that different cell lineages coordinate their morphogenesis to construct functional units. This requires self-organizing mechanisms that ensure that the right cells come into contact with each other and give rise to desired shapes. Such higher-order organization emerges from simple behaviours of individual cells guided by local information^1^. While many studies investigate self-organization within a lineage^2,3^, the literature is limited on functional studies elucidating mechanisms of self-organization across multiple cell lineages.

Oogenesis is a prime example of a developmental process that depends on the close interaction of two lineages. From invertebrates to vertebrates, oocytes develop within a germline cyst that is enveloped by somatic cells^4^. The germline cyst consists of the oocyte itself and so-called nurse cells^5–8^. The role of nurse cells is to supply the oocyte with essential materials during oogenesis, but eventually, nurse cells are removed to generate a single mature oocyte^6,7,9^. The maturation of the oocyte, as well as the removal of nurse cells is strictly dependent on the cooperation with somatic cells enveloping the germline cyst. These so-called follicle cells (FCs) differentiate into diverse cell fates, which, among others, specialize to facilitate oocyte maturation by eggshell secretion or nurse cell removal by phagoptosis^4,7,9–12^. Thus, it is essential that germline cells and somatic cells match each other, such that nurse cells are in contact with FCs that facilitate nurse cell removal, and the oocyte is in contact with FCs that enable oocyte maturation.

In *Drosophila* oogenesis, the establishment and maintenance of this match is a complex process that involves major cell redistributions. Oocytes develop within so-called egg chambers that consist of the germline cyst and a surrounding monolayer follicle epithelium. The germline cyst consists of 1 oocyte and 15 nurse cells, and the approximately 850 FCs differentiate into three major fates^4,10,12^. Whereas the fate of germline cells is determined already prior to egg chamber assembly^8^, FCs differentiate and specialize under the control of JAK/STAT, EGF and Notch signalling pathways during early egg chamber development^13–20^.

At the anterior tip of the egg chamber, approximately 10% of FCs differentiate into so-called anterior FCs (AFCs), which specialize to facilitate nurse cell removal and thus must cover the entire nurse cell compartment by mid-oogenesis. The remaining ~90% of FCs differentiate into main body FCs (MBFCs) and posterior FCs (PFCs), which specialize to facilitate oocyte maturation and thus must eventually cover the entire oocyte. However, at the time point of fate specification, germline cells and FCs do not match yet: MBFCs are initially in contact with nurse cells and AFCs cover only a small proportion of the nurse cell compartment^4,10,12,21,22^. Thus, FCs must redistribute over the germline surface to establish the right match. This redistribution must be coordinated with germline growth and changes in oocyte and nurse cell proportions, as the oocyte comprises only ~6% of the germline (1/16^th^) when FCs are specified but makes up ~40% of the germline in mid-oogenesis^21^. Consequently, germline and FCs must establish the right match under constantly changing morphologies. How germline and FCs coordinate to establish inter-lineage functional units essential to produce a fertile egg is currently not understood.

Here, we show that the transcriptional regulator Eyes absent (Eya), expressed in FCs, induces cell–cell affinity between nurse cells and Eya-expressing FCs in *Drosophila* oogenesis. Employing functional studies and phase field modelling, we demonstrate that differential cell–cell affinity and the resulting forces drive FC redistribution over the germline and control oocyte growth to establish a functional inter-lineage match between germline cells and FC populations.

## Results

### Egg chamber morphogenesis is divided into three phases with distinct soma-germline matching dynamics

**Fig. 1:**
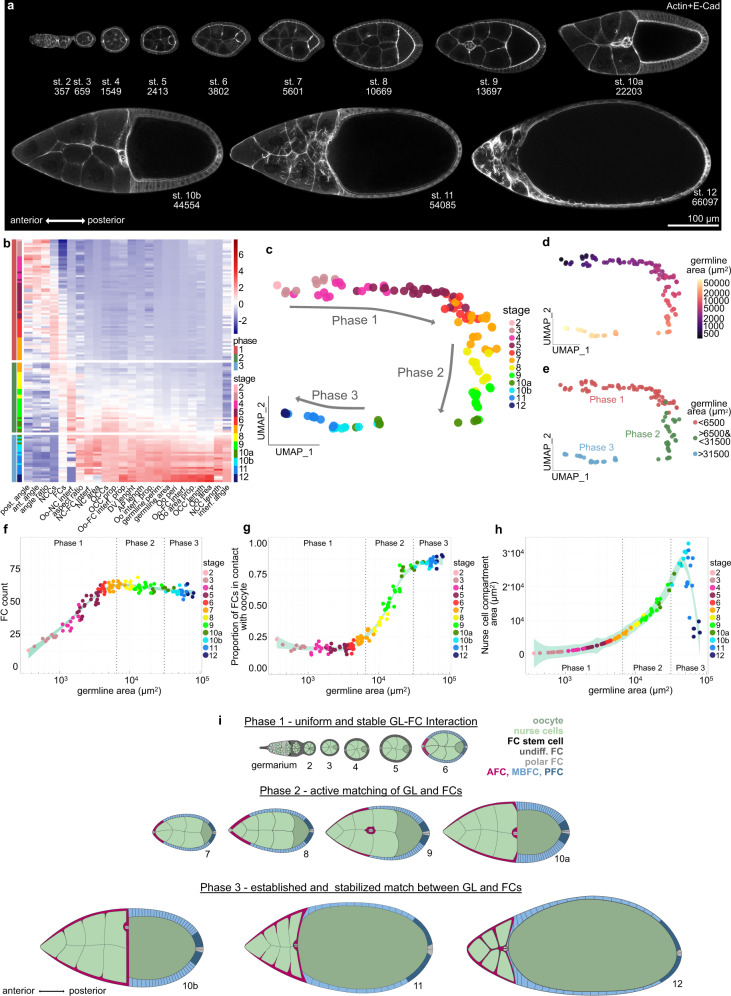
Egg chamber morphogenesis is divided into three phases with distinct soma-germline matching dynamics. **a** Medial confocal sections of wild type (wt) egg chambers depicting developmental stages from the germarium to stage 12, stained for E-Cad and F-Actin. Numbers denote germline areas in µm^2^. **b** Heatmap of the quantified 24 morphological parameters (see Supplementary Fig. 1). Each row represents an individual egg chamber, with increasing germline sizes from top to bottom. Breaks separate morphogenetic phases. **c** UMAP of multidimensional quantification of egg chamber morphogenesis. Egg chambers are coloured according to their respective developmental stage. Note that the developmental trajectory is subdivided into three phases. **d** UMAP with germline areas visualized. **e** UMAP with egg chambers assigned to the three phases based on their germline size. **f** Follicle cell (FC) count as a function of germline area. **g** Proportion of FCs in contact with the oocyte as a function of germline area. **h** Nurse cell compartment area as a function of germline area. Dotted lines mark germline sizes at the transition between two phases. All curves are LOESS fitted with a 95% CI area. *n* = 126 egg chambers (ECs). **i** Illustration of the three morphogenetic phases of *Drosophila* egg chamber development. See Supplementary Table 7 for detailed statistical information. Source data are provided as a Source Data file.

To analyse the dynamics of the soma-germline matching process, we performed an in-depth quantitative description of egg chamber morphogenesis. We quantified 24 morphological parameters in egg chambers from stages 2 to 12 (Fig. 1a, Supplementary Fig. 1). These parameters included among germline-, and FC-specific descriptors^21,23^, importantly, 10 parameters that characterized interactions between germline and FCs. To extract the dynamics of global egg chamber morphogenesis, we analysed the multidimensional dataset using UMAP (Uniform Manifold Approximation and Projection)^24^ (Fig. 1b, c). In the UMAP projection, individual egg chambers organized along a developmental trajectory of classical egg chamber stages (Fig. 1c). Importantly, germline sizes steadily increased along the trajectory, demonstrating that germline area can be used as a continuous variable representing developmental progression (Fig. 1d). This represents an advancement over classical egg chamber staging, which is dependent on morphological features often disrupted by genetic manipulations (for example ref. 22) and produces only a discrete description of a continuous process (see Methods).

The UMAP analysis revealed that egg chamber morphogenesis is subdivided into three phases. To characterize these phases, we assigned egg chambers based on their germline size to their respective phase and analysed individual morphological parameters as a function of germline size (Fig. 1e). As the differentiation of FCs into AFCs, MBFCs and PFCs at stage 6, is a crucial step for egg chamber development and coincides with an arrest of mitotic divisions^10,14,15^, we analyzed the number of FCs (Fig. 1f). We found that FCs cease to multiply, and thus receive the information with which germline cell they must match, at the end of phase 1 (Fig. 1f, i). To understand the dynamics of FC redistribution that matches FCs and germline cells, we analysed the proportion of FCs that was in contact with the oocyte (Fig. 1g). Throughout phase 1, 17 ± 3% of FCs were in contact with the oocyte. By phase 3, this proportion had increased to 84 ± 3%. Thus, the redistribution of FCs and therefore the active matching between FCs and germline cells is executed during phase 2 (Fig. 1i). This suggests that the right match is essential for phase 3. Indeed, nurse cell dumping, during which nurse cell volume decreases massively, has been shown to depend on the right match and takes place during phase 3^25,26^ (Fig. 1h, i).

Taken together, our multidimensional analysis reveals that global egg chamber morphogenesis is subdivided into three phases, which correlate with three distinct soma-germline interaction dynamics (Fig. 1i).

### FC distribution over germline cells co-evolve with Eya expression patterns

**Fig. 2:**
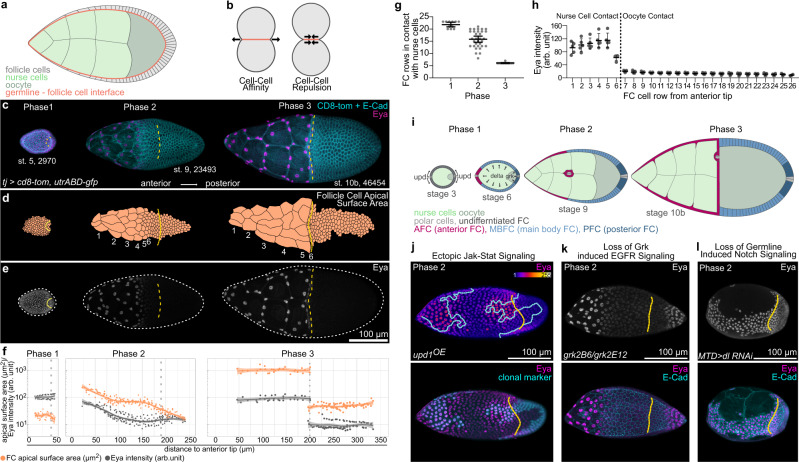
FC contact surfaces with germline cells correlate with Eya expression patterns. **a** Illustration of the germline-soma interface. **b** Illustration of cell–cell affinity and cell–cell repulsion. **c** Max projections of egg chambers expressing *CD8-tom* (membrane) and *utrABD-gfp* (actin) under the control of *tj-GAL4* (FC driver), stained for E-Cadherin (E-cad) and Eyes absent (Eya). Numbers denote germline area in µm^2^. **d** Segmented apical contact surfaces of FC with the germline of ECs shown in (**c**). Numbers denote FC rows from anterior to posterior. **e** Max projections of ECs from (**c**), depicting expression of Eya. White dotted lines mark EC outlines. **f** FC apical surface areas (orange) and Eya levels (grey) as a function of their distance to the anterior tip of ECs. Dotted lines mark the oocyte-nurse cell boundaries. Stage 4 ECs (phase 1, n = 45 FC of 2 ECs); stage 9 ECs (phase 2, n = 192 FC of 3 ECs); stage 10b ECs (phase 3, *n* = 112 FC of 3 ECs). Curves are LOESS fitted with a 95% CI area. **g** Count of FC rows in contact with nurse cells in wt EC. Mean+95% CI, n (Phase 1 = 11 EC, Phase 2 = 32 EC, Phase 3 = 12 EC). **h** Mean Eya fluorescence intensities in FC rows in phase 3 ECs (stage 10a & 10b). Mean±SD, n = 5 ECs. Dotted line separates FC rows in contact with nurse cells from FC rows in contact with the oocyte. **i** Illustration of FC patterning. During stage 3, polar cells release Upds and induce a JAK/STAT signalling gradient in nearby FCs. During stage 6, the oocyte activates EGFR signalling in oocyte-contacting cells through Grk. Dl from the germline activates Notch signalling in FCs, which allows FCs to adopt their fate. JAK/STAT + Notch = AFC, JAK/STAT + EGFR + Notch = PFC, Notch- only = MBFC. **j** Max projection of a phase 2 EC with clonal expression of *upd1* and *GFP*, stained for Eya. Ectopic JAK/STAT signalling leads to ectopic Eya gradients in MBFCs, but not PFCs. **k** Max projection of a phase 2 *grk(2B6) /grk(2E12)* (EGF) mutant EC, stained for Eya and E-Cad. Loss of EGFR signalling in PFCs leads to ectopic Eya expression. **l** Max projection of a phase 2 EC expressing *delta(dl)*-RNAi using *MTD-Gal4* (germline driver), stained for Eya and E-Cad. Loss of Notch signalling causes failure of Eya downregulation in MBFCs and PFCs. See Supplementary Table 7 for detailed statistical information. Source data are provided as a Source Data file.

The matching of FCs and germline cells must be coordinated by interactions at the soma-germline interface (Fig. 2a). Cell–cell interactions are controlled by surface tension at the interface^2,3^ and can be described along a spectrum of cell–cell affinity to cell–cell repulsion, where affinity causes an increase in contact size between cells and repulsion a decrease (Fig. 2b). We therefore quantified the size of apical FC surfaces, which are in contact with the germline, to characterize how FCs interact with germline cells (Fig. 2c, d, f). We found that throughout phase 1, contact areas of FCs were similar, suggesting that coverage of the available germline surface was evenly distributed among all FCs. However, during phase 2, a gradient in contact areas developed, with AFCs increasing their apical contact surfaces more rapidly than the remaining FCs. The gradient resolved by phase 3 and resulted in a segregation of FCs with large contact surfaces over nurse cells and FCs with comparatively small surfaces over the oocyte.

The gradual increase of AFC contact surfaces during phase 2 is called AFC flattening and is specific to AFC fate^22,27,28^. Previous studies suggested that the increase in AFC areas could solely be a result of AFCs being stretched within the epithelium to accommodate germline growth^12,21,29,30^. To test this idea, we reduced intra-epithelial cohesion by removing the cell–cell adhesion molecules E-Cadherin and/or N-Cadherin^3,22^ (Supplementary Fig. 2a, b). We found that this manipulation did not disrupt AFC expansion nor flattening. Secondly, we fully uncoupled individual AFCs by limiting cellular growth via ectopic expression of *hpo*^31^. As the germline grows, the reduced cell size of affected AFCs caused them to detach from each other. Yet, these FCs maximized contacts with nurse cells by spreading out via elaborate protrusions (Supplementary Fig. 2c). We therefore propose that AFCs expand apical surfaces independent of intra-epithelial cohesion by actively and autonomously increasing their contact surface with nurse cells.

In search for a regulator of FC interaction with the germline, we identified Eya (Eyes Absent). Eya is a highly conserved transcriptional co-regulator and phosphatase, and well-characterized for its role in eye specification^32–35^. Eya is also reported to distinguish FC fate from polar and stalk cell fate during egg chamber assembly and is used as a functionally uncharacterized marker for AFC fate^13,21,36–38^. We found that Eya expression patterns appeared with similar dynamics as apical FC surface sizes, with uniform expression in phase 1, a gradient in Eya levels from anterior to posterior during phase 2 and a strict segregation of Eya-positive FCs over nurse cells and Eya-negative FCs over the oocyte by phase 3 (Fig. 2e, f, Supplementary Fig. 2d, e). A cell row-wise analysis revealed that this dynamic robustly led to 6 rows of FCs in contact with nurse cells by phase 3 and confirmed that exclusively these 6 rows maintained Eya expression (Fig. 2g, h).

As Eya expression patterns track with AFC fate after phase 1 (Supplementary Fig. 2f), we asked if the signalling pathways determining AFC fate also control Eya dynamics. FC specification is controlled by the Jak-Stat, EGFR and Notch signalling pathways (Fig. 2i). Specifically, polar cells at each egg chamber pole secrete the ligand Upd and thereby induce a gradient of Jak-Stat signalling in surrounding FCs. In addition, the oocyte secretes the ligand Grk, which activates EGFR signalling in posterior cells. Lastly, the germline induces Notch signalling in all FCs by providing Delta, which allows FCs to adopt the fate they were primed for by Jak-Stat and EGFR signalling. Thus, FCs with Jak-Stat and Notch activation differentiate into AFCs, FCs with Jak-Stat, EGFR and Notch activation differentiate into PFCs and FCs which solely activate Notch become MBFCs^13–20^ (Fig. 2i). We found that Eya expression was positively regulated by an ectopic Upd-induced Jak-Stat signalling gradient (Fig. 2j, Supplementary Fig. 2g), negatively regulated by EGFR activation (Fig. 2k, Supplementary Fig. 2g) and that the switch from uniform levels to an anterior-posterior Eya-gradient was dependent on Notch signalling inducing FC differentiation (Fig. 2l, Supplementary Fig. 2g). Thus, by the end of phase 1, Eya expression becomes dependent on AFC fate specification and therefore tracks with AFCs during phase 2 and 3.

### Eya controls the size of FC contacts with germline cells

**Fig. 3:**
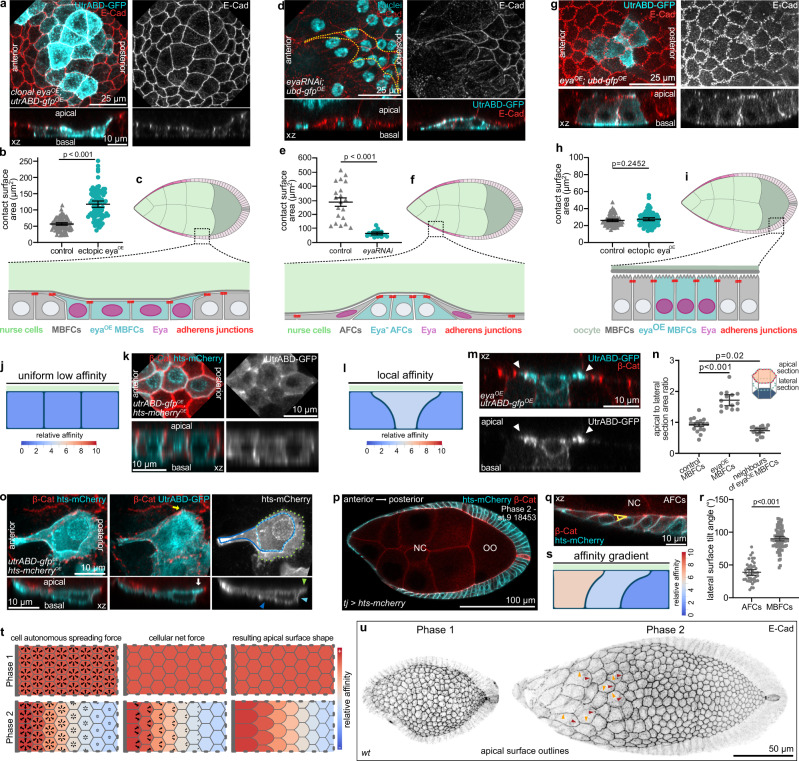
Eya expression in FCs induces Affinity for Nurse Cells. **a** MBFCs in contact with nurse cells during phase 2 with clonal expression of *utrABD-gfp* and ectopically expressing *Eya* (*eya*^*OE*^), stained for E-Cad. Apical surface projection and xz-reslice shown. **b** Quantification of apical contact surface areas of control MBFCs and *eya*^*OE*^-MBFCs in contact with nurse cells during phase 2. Mean+95% CI. Two-tailed Welch’s t-test. n (control: 71 MBFC, *eya*^*OE*^: 64 MBFC). **c** Illustration of cell morphologies upon ectopic *eya*^*OE*^ expression in MBFC clones in contact with nurse cells during phase 2. **d** AFCs in contact with nurse cells during phase 2 with clonal expression of *eya-RNAi*, stained for E-Cad and nuclei (DAPI). Yellow line depicts clonal outline. Apical surface projection and xz-reslice shown. **e** Quantification of apical contact surface areas of control and *eya-RNAi* AFCs during phase 2. Mean+95% CI, two-tailed Welch’s t-test, n (control: 20 AFCs, *eya-RNAi*: 20 AFCs). **f** Illustration of cell morphologies upon *eya-RNAi* expression in AFCs during phase 2. **g** MBFCs in contact with the oocyte during phase 2 with clonal expression of *utrABD-gfp* and *Eya *(*eya*^*OE*^), stained for E-Cad. Apical surface projection and xz-reslice shown. **h** Quantification of apical contact surface areas of control MBFCs and MBFCs ectopically expressing *Eya* (*eya*^*OE*^) in contact with the oocyte. Mean+95% CI. Unpaired Student’s *t*-test. n (control: 75 cells, *eya*^*OE*^*:* 83 cells). **i** Illustration of cell morphologies upon *Eya* (*eya*^*OE*^) expression in MBFC clones in contact with the oocyte during phase 2. **j** Phase field model of 3 FCs in contact with nurse cells with low and equal affinities. **k** MBFCs in contact with nurse cells during phase 2 with clonal expression of *utrABD-gfp* and *hts-mCherry* (membrane), stained for β-catenin. Apical surface projection and xz-reslice shown. **l** Phase field model of 3 FCs in contact with nurse cells with the central cell developing a relatively higher affinity. **m** MBFCs in contact with nurse cells during phase 2 with one MBFC expressing *utrABD-gfp* and *Eya* (*eya*^*OE*^), stained for β-catenin. xz-reslice shown. White arrowheads point to apical actin-rich protrusions extending towards neighbouring Eya-negative FCs. **n** Quantification of apical to lateral area ratios (see illustration and Fig S3a) for control MBFCs, *eya*^*OE*^-MBFCs and direct neighbours of *eya*^*OE*^-MBFCs. Mean+95% CI, Welch one-way Anova with Dunnett’s T3 multiple comparisons test. n (control MBFCs: 16 cells, *eya*^*OE*^-MBFCs: 14 cells, neighbours: 18 cells). **o** AFCs in phase 2 with clonal expression of *utrABD-gfp* and *hts-mcherry*, stained for β-catenin. Dotted lines outline apical (green), lateral (light blue) and basal (dark blue) surfaces. Arrowhead of same colour marks surfaces in xz. Yellow arrow points at actin-based filopodium. White arrow points at actin rich apical surface protrusion. Max projection of whole cell and xz-reslice shown. **p** Medial confocal section of a phase 2 egg chamber expressing *hts-mCherry* under the control of *tj-GAL4* (FC driver), stained for β-cat. Germline area in µm^2^. **q** Tilt of lateral membranes in AFCs. Angle for quantification is depicted in yellow. **r** Quantification of angles between lateral membranes and the germline surface in AFCs and MBFCs. Mean+95% CI, two-tailed unpaired t-test, n (45 AFCs, 82 MBFCs, 4 ECs). **s** Phase field model of 3 FCs with an affinity gradient. **t** Illustration of cell-autonomous spreading as a function of affinity, the resulting forces and apical surface shapes as a result of phase 1 and 2 affinity patterns. Grey bar represents anterior tip in egg chambers. **u** Local z-projection of the FC junctional network. wt ECs, stained for E-Cad. Orange arrowheads point at junctions between cells of the same row that remain straight, and red arrowheads point at convex junctions between FCs of different rows with different affinities. See Supplementary Table 7 for detailed statistical information. Source data are provided as a Source Data file.

The correlation between Eya patterns and FC contact surfaces in combination with the clear segregation of Eya-positive FCs over nurse cells and Eya-negative FCs over the oocyte made us question whether Eya played a role in soma-germline matching. To test this, we manipulated Eya expression in FC clones during phase 2, when FC-germline matching takes place. First, we ectopically expressed Eya in MBFC clones, which normally lose Eya expression and transition onto the oocyte. Ectopic Eya was sufficient to cause an increase of MBFC surfaces in contact with nurse cells, which occurred via broad apical protrusions extending towards and displacing apical surfaces of neighbouring Eya-negative MBFCs (Fig. 3a–c). Next, we reduced Eya expression in AFCs, which normally express Eya and expand their contact with nurse cells and found that clonal expression of *eya-RNAi* caused a failure of contact surface increase (Fig. 3d–f). Lastly, we ectopically expressed Eya in MBFC clones, which had already transitioned onto the oocyte and found that Eya expression had no effect on the apical surface size of FCs in contact with the oocyte (Fig. 3g–i). Thus, Eya induces FCs to expand their contact surface exclusively with nurse cells, which led us to hypothesize that Eya causes FCs to experience cell–cell affinity towards nurse cells, but not towards the oocyte.

Interestingly, as Eya is also expressed in the two somatic cells that enwrap each germline cyst in testis, we asked if Eya might also control affinity-like interactions in developing spermatocytes^39,40^. We found that the somatic cells closely envelope each cell within the cyst and thereby maximize the soma-germline interface (Supplementary Fig. 3a, c). When we expressed *eya-RNAi* in somatic cells, they failed to extend in between germline cells (Supplementary Fig. 3b, c) and caused an overall change in spermatocyte morphology reflecting a minimization of the soma-germline interface (Supplementary Fig. 3d). Thus, Eya controls soma-germline interfaces, possibly via regulating soma-germline affinity, in both ovaries and testis.

### Eya induces FC affinity for nurse cells in a level-dependent manner

To test if cell–cell affinity between Eya-positive FCs and nurse cells alone could account for the observed FC morphologies, we designed a phase field model that allowed us to simulate cell shapes as a function of interface dynamics^41,42^. We modelled 3 FCs and specified affinity as the energetic preference of an FC to be in contact with a defined boundary, representing the nurse cell surface (Supplementary Fig. 3e). First, we assigned low and equal affinities to all 3 FCs, simulating low Eya levels in MBFCs during phase 2 (Fig. 3j). This gave rise to an even distribution of FCs with equal germline-contacting surfaces, recapitulating the shape of phase 2 MBFCs (Fig. 3j, k). Next, we replicated clonal ectopic expression of Eya by assigning higher affinity to the central cell (Fig. 3l, Supplementary Movie 1). This caused the central cell to expand its contact with the simulated nurse cell surface at the expense of neighbouring cells, recapitulating the dominant apical surface expansion of MBFCs with ectopic Eya expression (Fig. 3m, n, Supplementary Fig. 3e). Thus, translating Eya levels into differential affinities towards nurse cells was sufficient to account for Eya-dependent FC shapes.

To further explore how Eya levels determine the interaction of FCs with nurse cells, we analysed AFCs during phase 2. As described before, AFCs expand their contact surface with nurse cells in a gradient^27^, which positively correlated with Eya levels (Supplementary Fig. 3f). We found that AFCs increased their contact surface with nurse cells in a polarized manner by extending a broad actin-rich protrusion posteriorly (Fig. 3o). The polarized apical expansion deformed posterior adherens junctions, and coincided with a trailing edge-like structure on the basal side and a tilt in lateral membranes (Fig. 3o–r). To test if Eya-controlled affinity for nurse cells could give rise to such polarized cell morphologies during phase 2, we assigned a gradient of affinities based on Eya levels to the 3 simulated FCs. This recapitulated polarized protrusions of “apical” surfaces towards decreasing affinities and a tilt of lateral membranes (Fig. 3s, Supplementary Movie 2). Thus, an Eya-dependent gradient in affinity for nurse cells can account for the polarized expansion of AFCs during phase 2.

Importantly, Eya-controlled affinity for nurse cells could also account for the prominent differences in FC shapes during phase 1 and 2 (Fig. 3t, u). During phase 1, uniform Eya levels in FCs translate into uniform apical expansion forces that are balanced at cell–cell junctions and thereby give rise to a regular quasi-hexagonal arrangement of cells. In contrast, an affinity gradient causes imbalanced expansion forces at junctions, which resolve into a polarized expansion towards decreasing affinities giving rise to the observed fish-scale like pattern in AFCs during phase 2. The experimental data combined with the mathematical modelling of cellular behaviors as a function of affinity propose that Eya induces level-dependent affinity of the apical FC surface for the nurse cell surface and thereby controls FC shapes.

### Eya-controlled affinity dynamics account for FC distribution over germline cells

**Fig. 4:**
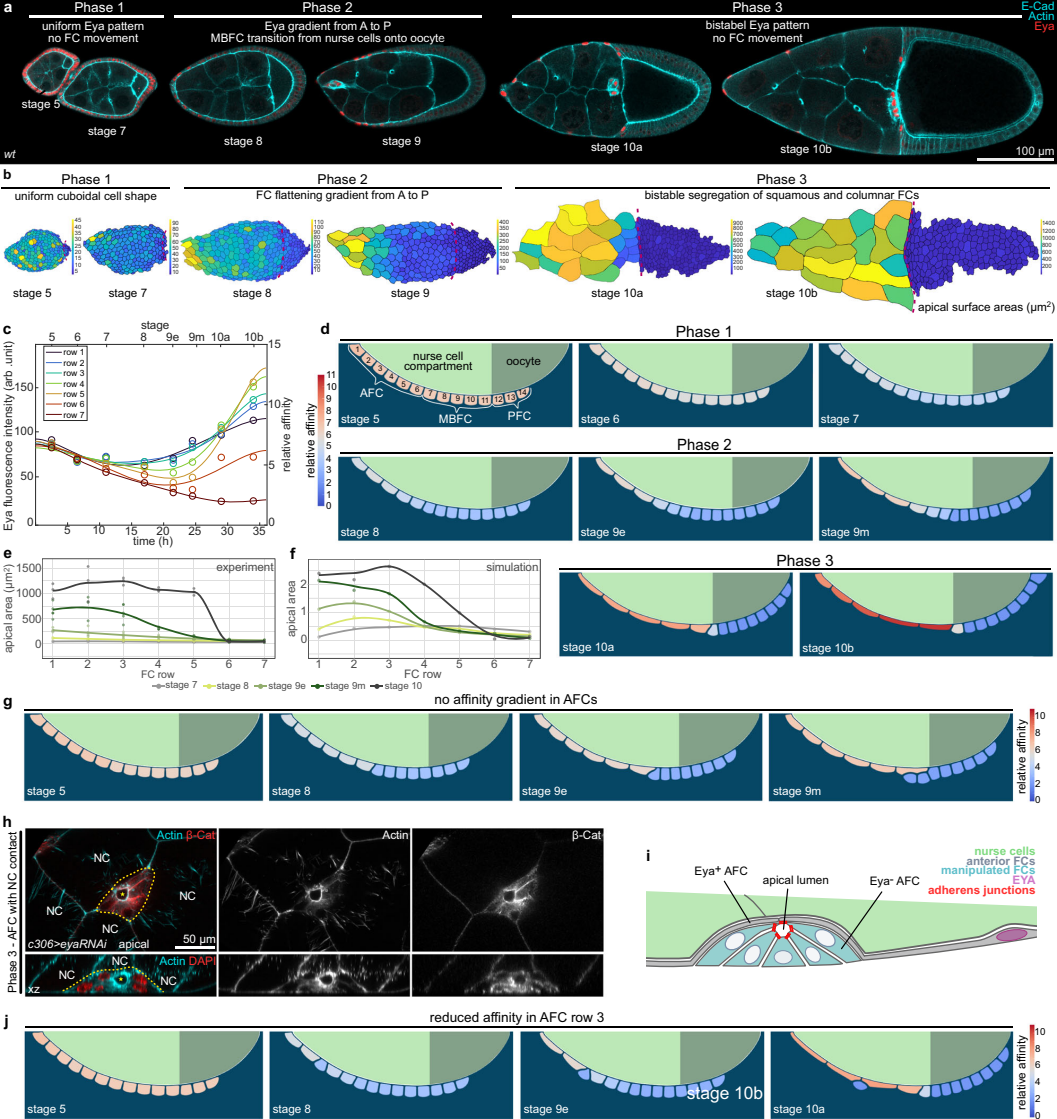
Eya-regulated affinity accounts for FC dynamics throughout egg chamber development. **a** Medial confocal sections of ECs stained for E-Cad, F-Actin and Eya. **b** Segmented apical surface areas of FCs in ECs. Red dotted line marks nurse cell–oocyte boundary. Colour scale bar shows apical surface area sizes in (µm^2^). **c** Average Eya fluorescence intensities of the 7 anterior rows as a function of time. Time denotes hours after the beginning of stage 5. Intensities are assigned to the midpoint of each stage. Eya dynamics serve as proxy for affinity dynamics in simulations (Supp. File S1). Row 1-6 dynamics were assigned to cells 1-6 (AFCs) and row 7 dynamic was assigned to cells 7–14 (MBFCs) in the phase field model. n (stage 5: 5 ECs, stage 6: 5 ECs, stage 7: 5 ECs, stage 8: 4 ECs, stage 9e: 8 ECs, stage 9m: 9 ECs, stage 10a: 5 ECs, stage 10b: 5 ECs). 6th order polynomial fit constrained to have vanishing derivatives at *t* = 0 and *t* = 36 hours. **d** Phase field model simulating collective behaviour of FCs as a function of their affinity for germline cells. wt affinity dynamics are based on measured Eya levels. **e** Apical surface area of the 7 anterior cell rows in stage 7-10 wt ECs. Apical surface area gradient first appears at stage 8. Stage 10a and 10b were pooled (stage 10). n (stage 7: 4 ECs, stage 8: 3 ECs, stage 9e: 5 ECs, stage 9 m: 5 ECs, stage 10: 3 ECs). **f** Apical surface areas of the 7 anterior cells in the simulation ((apical length)^2^). Apical surface area gradient first appears at stage 8 and develops with similar dynamics as observed in vivo. **g** Phase field model simulating collective behaviour of FCs as a function of their affinity for germline cells. All 6 AFCs share row 1 high-affinity dynamics. **h** AFCs expressing *eya-RNAi* under the control of *c306-GAL4* (variegated AFC driver, c306>*eya-RNAi*). AFCs of a phase 3 EC, stained for β-cat, F-Actin and nuclei (DAPI). Formation of an apical lumen (yellow star) in *eya-RNAi* AFC cluster (yellow dotted line). NC marks nurse cells. Section through apical lumen and xz-reslice shown. **i** Illustration of cell morphologies upon *eya-RNAi* knockdown in a group of AFCs in contact with nurse cells during phase 3. **j** Phase field model simulating the collective behaviour of FCs as a function of their affinity for germline cells. Reduction in affinity in row 3 causes a failure to flatten and drives displacement of AFC from the nurse cell surface. See Supplementary Table 7 for detailed statistical information. Source data are provided as a Source Data file.

To test if Eya-controlled affinity is sufficient to account for the shape as well as distribution of FCs throughout the 3 phases of egg chamber morphogenesis (Fig. 4a, b), we designed a more elaborate phase field model (Supplementary Fig. 4a–d). We modelled 14 FCs representing 6 rows of AFCs, and for simplicity, just 5 rows of MBFCs and 3 rows of PFCs (Supplementary Fig. 4b). The boundary, representing the germline surface, was divided into an affine (nurse cells) and non-affine (oocyte) compartment (Supplementary Fig. 4c). Our model did not include germline growth and FC volumes were set to be constant. We measured Eya levels in rows 1-6 (AFCs) and row 7 (MBFC) in egg chambers during stages 5-10b (phase 1-3) and used the estimated length of developmental stages^12,43^ to interpolate the temporal development of Eya levels within each row (Fig. 4c). The resultant Eya dynamics were then used as direct proxy for affinity dynamics, with row 7 dynamics being assigned to rows 7–14 (MBFCs & PFCs). Simulating affinity based on measured Eya levels was sufficient to recapitulate FC behavior throughout development (Fig. 4d, Supplementary Movie 3). During phase 1, all FCs had similar contact surface sizes, cuboidal shapes and maintained their relative positions. During phase 2, AFCs progressively increased their contact surface in a gradient from anterior to posterior and consequently displaced MBFCs (row 7-11) onto the oocyte (Fig. 4e, f). Eventually, in phase 3, FCs stably segregated with high affinity cells in contact with nurse cells and low affinity cells positioned over the oocyte. Thus, simulating FC behavior based on Eya-controlled affinity recapitulates FC positions, shapes and contact surface sizes throughout all three phases and is sufficient to establish a match between Eya-positive AFCs and nurse cells, as well as between Eya-negative MBFCs+PFCs and the oocyte.

To understand if the Eya-gradient itself is required to drive proper matching of AFCs with nurse cells during phase 2, we abolished the gradient in simulations by assigning all 6 AFC rows the high-affinity dynamic of row 1 (Fig. 4g, Supplementary Movie 4). This disrupted the AFC surface area gradient during phase 2, as recapitulated by experimental data (Supplementary Fig. 4e, f), but more significantly, caused row 6 to displace row 7 and 8 from the nurse cell surface. This suggests that steep affinity differences between neighboring cells result in forces that are strong enough to displace low-affinity cells from the germline surface.

Lacking an experimental setup to manipulate specifically the Eya-gradient in AFCs during phase 2, we created steep differences in affinity between individual AFCs by expressing *eya-RNAi* in small AFC clones. We observed that single *eya-RNAi*-expressing AFCs as well as *eya-RNAi*-expressing AFC clones lost nurse cell contact (Fig. 4h, i, S4g, h). Single cells extruded as spheres, while clones retained epithelial features and formed a cyst with an apical lumen. When we recapitulated this experiment in simulations by reducing the affinity of cell row 3, row 3 failed to increase its contact surface area with nurse cells and was eventually displaced from the germline surface (Fig. 4j, Supplementary Movie 5). Thus, steep differences in Eya levels and the resulting differences in affinities cause displacement and exclusion of low affinity FCs from nurse cells. Consequently, the gradient in Eya levels during phase 2 is essential to retain all FCs in contact with the germline while driving the redistribution of FCs to establish the right match between FCs and germline cells.

### Controlling FC distribution over the germline surface by controlling Eya-expression

**Fig. 5:**
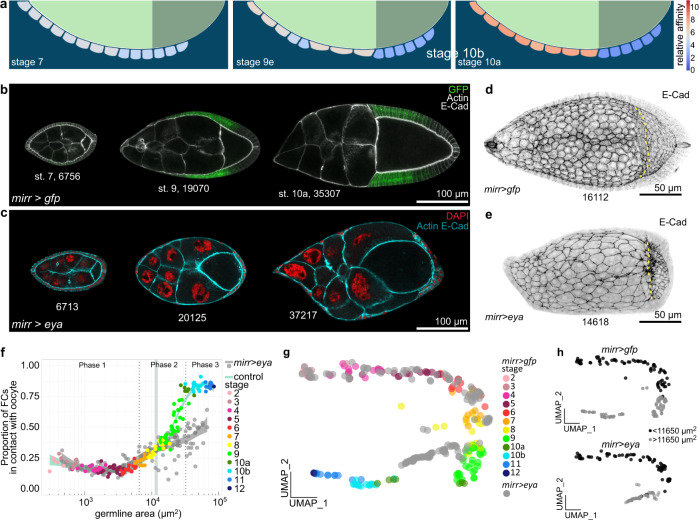
Ectopic Eya expression in MBFCs matches MBFCs with nurse cells, instead of the oocyte. **a** Phase field model simulating the collective behaviour of FCs as a function of their affinity for germline cells. An ectopic increase of affinity in rows 6-8 was simulated. Rows ectopically increase contact with nurse cells and fail to transition onto the oocyte. **b** Medial confocal sections of egg chambers from stage 7 to 10a expressing *gfp* under the control of *mirr-GAL4* (*mirr* > *gfp*, MBFC driver), stained for E-Cad and F-Actin. Note how GFP-positive cells shift from nurse cells onto the oocyte. Numbers denote germline areas in µm^2^. **c** Medial confocal sections of egg chambers expressing *Eya* (*eya*^*OE*^) under the control of *mirr-GAL4* (*mirr* > *eya*^*OE*^, MBFC driver), stained for E-Cad, F-Actin and nuclei (DAPI). Numbers denote germline areas in µm^2^. **d** Local z-projection of the FC junctional network of an egg chamber expressing *gfp* under the control of *mirr-GAL4* (*mirr* > *gfp*, MBFC driver), stained for E-Cad. Yellow dotted line marks oocyte-nurse cell boundary. Number denotes germline area in µm^2^. **e** Local z-projection of the FC junctional network of an egg chamber expressing *Eya* (*eya*^*OE*^) under the control of *mirr-GAL4* (*mirr* > *eya*^*OE*^, MBFC driver), stained for E-Cad. Yellow dotted line marks oocyte-nurse cell boundary. Number denotes germline area in µm^2^. **f** Proportion of FCs contacting the oocyte as a function of germline area of *mirr* > *gfp* and *mirr* > *eya*^*OE*^ egg chambers. Bluegrey area marks 95% CI of the critical size (10632–12569 µm^2^, see Supplementary Fig. 5). n (*mirr* > *gfp*: 153 EC; *mirr* > *eya*^*OE*^: 157 EC). **g** UMAP comparing *mirr* > *gfp* and *mirr* > *eya*^*OE*^ egg chamber morphogenesis. *mirr* > *eya*^*OE*^ trajectory diverts from control trajectory during phase 2. **h** UMAP plot of *mirr* > *gfp* and *mirr* > *eya*^*OE*^ grouped into egg chambers with germline sizes smaller (black) or larger (grey) than the critical size (11650 µm^2^, see Supplementary Fig. 5). Note how the switch from black to grey in the *mirr* > *eya*^*OE*^ trajectory correlates with the point of diversion from the control trajectory. See Supplementary Table 7 for detailed statistical information. Source data are provided as a Source Data file.

If Eya levels determine whether FCs remain in contact with nurse cells or not, we should be able to control FC distribution by simply changing Eya expression patterns. To test if we can retain more FCs in contact with nurse cells, we assigned the high affinity dynamic of row 2 to rows 6-8 in our model (Fig. 5a, Supplementary Movie 6). The simulation revealed an ectopic increase of their contact surfaces with nurse cells and a failure of their transition onto the oocyte, resulting in a reduced number of FCs in contact with the oocyte by phase 3. Experimentally, we tested this hypothesis by forcing MBFCs to ectopically express Eya after phase 1 using *mirr*-GAL4 (*mirr* > *eya*) (Fig. 5b, c). In control egg chambers, the first *mirr*-GAL4 positive cell row reached the oocyte at a germline size of 11650 µm^2^ (95% CI: 10630–12570 µm^2^) (Supplementary Fig. 5a–d), defining a critical size at which ectopic *eya* expression (*eya*^*OE*^) by *mirr*-GAL4 was expected to prevent further FC transition onto the oocyte. As predicted by the simulation, *mirr* > *eya* MBFCs ectopically increased their contact surface with nurse cells and failed to transition onto the oocyte once the critical size was reached (Fig. 5d–f, Supplementary Fig. 5e). The forced mismatch of Eya-expressing MBFCs with nurse cells caused the UMAP trajectory to divert from the control trajectory exactly at the critical germline size, highlighting the importance of appropriate germline-soma matching for overall egg chamber morphology (Fig. 5g, h, Supplementary Fig. 5f–h).

Thus, Eya expression patterns and resulting differential affinities dictate FC distribution over the germline. Accordingly, linking Eya expression with AFC fate ensures robust matching of AFCs with nurse cells and MBFCs+PFCs with the oocyte.

### Oocyte growth dynamics correlate with Eya-expression patterns in FCs

**Fig. 6:**
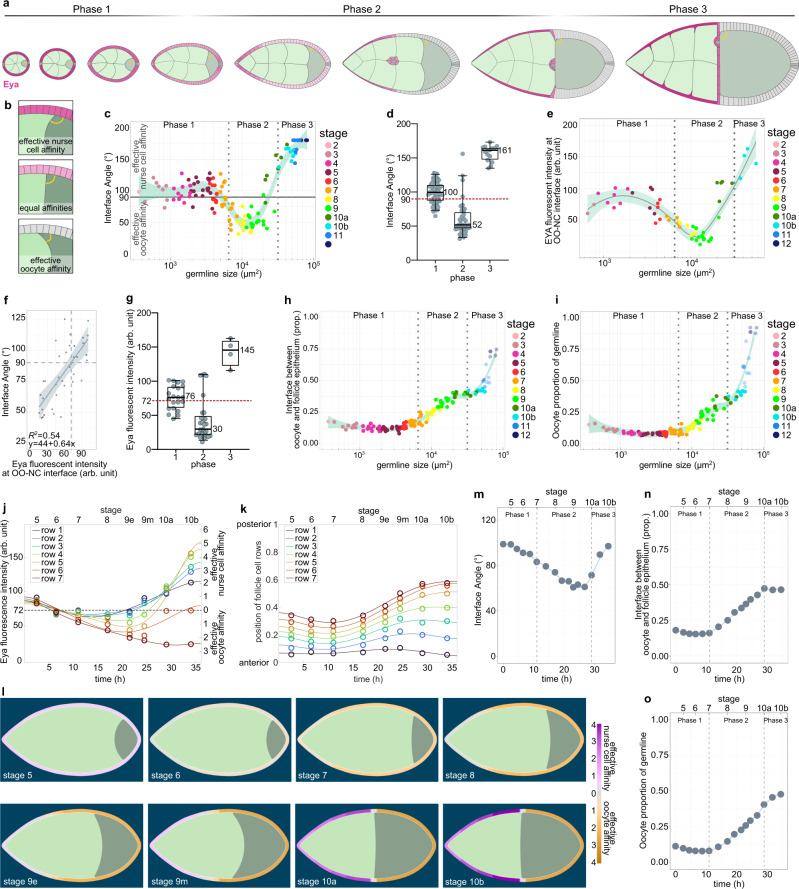
Eya-controlled effective germline affinities account for oocyte growth and shape dynamics. **a** Illustrations of ECs depicting the interface angle and Eya expression patterns. **b** Illustration of the interface angle as a parameter characterizing effective affinities of oocyte or nurse cells towards FCs. **c** Interface angle as a function of the germline area. Dotted lines mark germline sizes at the transition between two phases. LOESS fitted with a 95% CI area. *n* = 126 ECs. **d** Interface angle of wt ECs grouped into the three morphogenetic phases. Box plot with whiskers marking the 5th and 95th percentile. Numbers state the median of the corresponding phase. n (phase 1: 62 ECs, phase 2: 39 ECs, phase 3: 15 ECs) **e** Eya levels in FCs at the nurse cell–oocyte boundary as a function of germline area. Dotted lines mark germline sizes at the transition between two phases. LOESS fitted with a 95% CI area. *n* = 54 ECs. **f** Linear regression between the interface angle and Eya levels of FCs in contact with the nurse cell–oocyte boundary. Dashed line marks 90° angle. Linear regression with 95% CI area, *n* = 36 ECs. **g** Eya levels of FCs at nurse cell–oocyte boundary of wt ECs grouped into the three morphogenetic phases. Box plot with whiskers marking the 5th and 95th percentile. Number states median of the corresponding phase. n (phase 1: 19 ECs, phase 2: 29 ECs, phase 3: 4 ECs) **h** Oocyte–FC interface proportion of germline-FC interface as a function of germline area. LOESS fitted with a 95% CI area. *n* = 126 ECs. **i** Oocyte area proportion of the germline as a function of the germline area. Dotted lines mark germline sizes at the transition between two phases. LOESS fitted with a 95% CI area. *n* = 126 ECs. **j** Average Eya fluorescence intensity of the first 7 anterior FC rows as a function of time. Time denotes hours after the beginning of stage 5. Eya dynamics of rows serve as proxy for effective affinity dynamics implemented in simulations (Supp. File S1). n (stage 5: 5 ECs, stage 6: 5 ECs, stage 7: 5 ECs, stage 8: 4 ECs, stage 9e: 8 ECs, stage 9 m: 9 ECs, stage 10a: 5 ECs, stage 10b: 5 ECs). 6th order polynomial fit constrained to have vanishing derivatives at *t* = 0 and *t* = 36 hours. **k** Average normalized distances to anterior pole of the 7 anterior FC rows as a function of time. Distances and intensity dynamics were used to simulate effective affinities of germline cells (Supp. File S1). Row 7 affinity dynamic was assigned from the distance of row 7 to the posterior pole. n (stage 5: 5 ECs, stage 6: 5 ECs, stage 7: 5 ECs, stage 8: 4 ECs, stage 9e: 8 ECs, stage 9 m: 9 ECs, stage 10a: 5 ECs, stage 10b: 5 ECs). **l** Phase field model simulating germline cell behaviour as a function of their affinity for the follicle epithelium. wt affinity dynamics are based on measured Eya levels. **m–o** Individual morphological parameters of the simulation as a function of time. **m** Interface Angle. **n** Oocyte-FC interface proportion of the germline-FC interface. **o** Oocyte proportion of the germline. Phase boundaries were assigned to mid stage 7 and mid stage 10a. See Supplementary Table 7 for detailed statistical information. Source data are provided as a Source Data file.

After characterizing how Eya expression in FCs controls their interaction with the germline, we switched perspectives and asked if Eya expression in FCs also affects how germline cells interact with FCs. To analyse a possible differential interaction of nurse cells and the oocyte with the follicle epithelium, we characterized the angle at the interface, where oocyte and nurse cells compete for FC contact (Fig. 6a). The angle depicts which germline cell type preferentially expands its contact surface with the follicle epithelium, and thereby serves as a read-out for whether nurse cells or the oocyte harbour effective affinity for the follicle epithelium (Fig. 6b). An angle of 90° is the result of balanced forces, whereas an angle >90° represents effective nurse cell affinity and an angle <90° effective oocyte affinity. We found that the angle was larger than 90° during phase 1, decreased below 90° during phase 2, and increased above 90° again in phase 3 (Fig. 6c, d). This suggested that the oocyte harbours effective affinity for FCs exclusively during phase 2. We then analysed Eya levels in FCs overlying the nurse cell–oocyte boundary and found that these appeared with similar dynamics as the interface angle (Fig. 6e). To characterize the relationship between Eya levels and the interface angle, we performed a linear regression of the interface angle as a function of Eya levels and found a positive correlation, with Eya levels <72 arb. unit predicting an interface angle <90° (effective oocyte affinity) and Eya levels >72 arb.unit predicting an interface angle >90° (effective nurse cell affinity) (Fig. 6f). A phase-wise analysis suggested that exclusively during phase 2 Eya levels in FCs at the nurse cell–oocyte boundary are low enough to establish effective oocyte affinity (Fig. 6g). In line with that, we found that the contact surface of the oocyte with the follicle epithelium only started to increase at the transition from phase 1 to phase 2 and came to a halt at the end of phase 2, before it further increased during nurse cell dumping in phase 3 (Fig. 6h). Remarkably, the increase in interface with the follicle epithelium was accompanied by an increase in oocyte size (Fig. 6i).

### Eya-controlled effective germline affinities account for oocyte growth and shape dynamics

We therefore hypothesized that Eya levels in FCs non-cell autonomously regulate the contact surfaces of germline cells with the follicle epithelium and that this subsequently controls oocyte expansion. To test this hypothesis, we designed a phase field model, simulating the oocyte and the nurse cell compartment, and defined the outer boundary as the contact surface with FCs (Supplementary Fig. 6a, b). We quantified Eya levels in FC rows from stage 5-10b and the row’s relative position along the AP-axis, covering all three morphogenetic phases (Fig. 6j, k). We used these spatio-temporal dynamics as proxy for effective-affinity-inducing conditions on the boundary, with Eya levels of 72 arb.unit inducing 0 effective affinity (Fig. 6j). The resulting simulation recapitulated interface angle and oocyte expansion dynamics very well (Fig. 6l, Supplementary Movie 7). The interface angle was >90° during phase 1 and dropped below 90° at the end of phase 1, when the switch from effective nurse cell to effective oocyte affinity took place (Fig. 6m). This switch also caused an expansion of the oocyte contact surface with FCs and oocyte growth (Fig. 6n, o). Once, the oocyte had expanded along the entire Eya-negative FC surface, the angle increased above 90° and the oocyte seized to grow. Thus, these simulations suggest that Eya in FCs controls effective germline affinity for the follicle epithelium, causing the oocyte to expand its surface specifically along Eya-negative FCs. Next to Eya-controlled FC redistributions, this would result in an additional morphogenetic dynamic ensuring the establishment of the right match between FCs and germline cells.

### Premature loss of Eya in FCs during phase 1 induces premature oocyte expansion

**Fig. 7:**
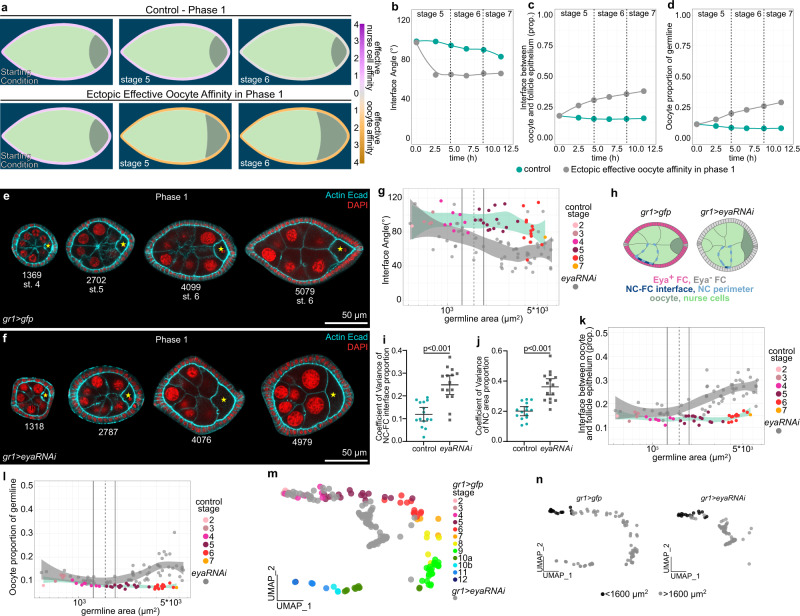
Premature loss of Eya in FCs during phase 1 induces premature oocyte expansion. **a** Phase field model simulating germline cell behaviour as a function of their affinity for the follicle epithelium. wt affinity vs. ectopic effective oocyte affinity in phase 1 (simulating phase 1 FC Eya loss). **b**–**d** Individual morphological parameters of the simulation as a function of time for phase 1. **b** Interface Angle. **c** Proportion of the germline-FC interface made up by the oocyte. **d** Oocyte proportion of the germline. **e**, **f** Medial confocal sections of phase 1 ECs expressing *gfp* (**e**) or *eya-RNAi* (**f**) under the control of *gr1-GAL4* (*gr1* > *gfp*, *gr1* > *eya-RNAi*, FC driver), stained for E-Cad, F-Actin and nuclei (DAPI). Yellow stars mark oocytes. Numbers denote germline areas in µm^2^. **g** Interface Angle as a function of germline area of *gr1* > *gfp* and *gr1* > *eya-RNAi* ECs in phase 1 (germline area <6500µm^2^). Dotted line marks critical germline area and solid lines mark 95% CI. All curves are LOESS fitted with a 95% CI area. n (*gr1* > *gfp* = 42 ECs, *gr1* > *eya-RNAi* = 60 ECs). **h** Illustrations of *gr1* > *gfp* and *gr1* > *eya-RNAi* egg chambers in phase 1 (stage 6). **i** Quantification of the coefficient of variance (CV) of the nurse cell-FC interface (dark blue line) proportion of the nurse cell perimeter (light blue dotted line) within a nurse cell cluster. Mean+95%CI, two-tailed unpaired Student’s *t*-test, n (*gr1* > *gfp*: 15 ECs, 71 NCs; *gr1* > *eya-RNAi*: 15 ECs, 89 NCs). **j** Quantification of the coefficient of variance (CV) of nurse cell area proportions within a nurse cell cluster. Mean+95%CI, two-tailed unpaired Welch’s t-test, n (*gr1* > *gfp*: 15 ECs, 71 NCs; *gr1* > *eya-RNAi*: 15 ECs, 89 NCs). **k** Oocyte-FC interface proportion of germline-FC interface as a function of germline area of *gr1* > *gfp* and *gr1* > *eya-RNAi* ECs in phase 1 (germline area <6500 µm^2^). **l** Oocyte area proportion of the germline area as a function of germline area of *gr1* > *gfp* and *gr1* > *eya-RNAi* ECs in phase 1 (germline area <6500µm^2^). Dotted line marks critical germline area, solid lines mark 95% CI of critical germline area. Curves are LOESS fitted with a 95% CI area. n (*gr1* > *gfp* = 42 ECs, *gr1* > *eya-RNAi* = 60 ECs). **m** UMAP comparing *gr1* > *gfp* and *gr1* > *eya-RNAi* EC morphogenesis. **n** UMAP of *gr1* > *gfp* and *gr1* > *eya-RNAi* coloured by germline sizes smaller (black) and larger (grey) than the critical size (1600 µm^2^, see Supplementary Fig. 7). See Supplementary Table 7 for detailed statistical information. Source data are provided as a Source Data file.

To test the role of FC Eya expression in controlling oocyte expansion, we first manipulated Eya expression in phase 1. We hypothesized that the uniform expression of Eya above the critical level of 72 arb.unit during phase 1 resulted in effective nurse cell affinity, which prevented the oocyte from increasing its contact with FCs and grow in size. In line with that, simulating an early switch to effective oocyte affinity during phase 1 led to a premature decrease in the interface angle below 90° and a premature expansion of the oocyte (Fig. 7a–d, Supplementary Movie 8). Experimentally, we reduced Eya levels in phase 1 egg chambers by GR1-GAL4 driven *eya-RNAi* (*gr1* > *eya-RNAi*) (Fig. 7e, f), which led to a substantial decrease in Eya levels when egg chambers reached a germline size of 1600 µm^2^ (95% CI: 1300–1918 µm^2^) (Supplementary Fig. 7a, b). The loss of Eya caused a premature decrease of the interface angel below 90°, indicating a switch from effective nurse cell affinity to effective oocyte affinity (Fig. 7g, Supplementary Fig. 7c). The premature loss of effective nurse cell affinity also caused a loss of nurse cell organization within the germline cyst (Fig. 7h). In control egg chambers, nurse cells were of similar sizes and distributed evenly along the follicle epithelium, whereas a loss of Eya in FCs resulted in a high variance of nurse cell-FC interface lengths and nurse cell sizes (Fig. 7i, j, Supplementary Fig. 7d, e). Thus, Eya expression in FCs non-autonomously controls nurse cell arrangement and morphology. Furthermore, as predicted by simulations, the *eya-RNAi*-driven switch from effective nurse cell affinity to effective oocyte affinity caused a premature expansion of the oocyte-FC interface and oocyte size (Fig. 7k, l, Supplementary Fig. 7f–h). The disruption of the *gr1* > *eya-RNAi* UMAP trajectory from the control during phase 1 further highlighted the importance of FC Eya expression and the resulting soma-germline affinity for global egg chamber morphogenesis (Fig. 7m, n). Taken together, during phase 1, uniform Eya levels in FCs above the critical level give rise to effective nurse cell affinity, which inhibits premature oocyte expansion and is essential to organize the germline cyst.

### Ectopic Eya expression in MBFCs inhibits oocyte expansion

**Fig. 8:**
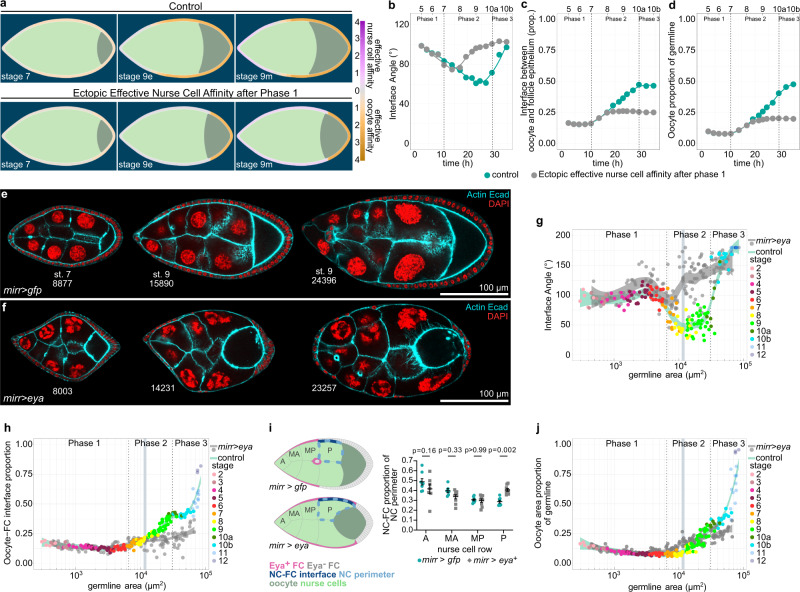
Ectopic Eya expression in MBFCs induces ectopic effective nurse cell affinity and inhibits oocyte growth. **a** Phase field model simulating germline cell behaviour as a function of their affinity for the follicle epithelium. wt affinity dynamics vs. ectopic effective nurse cell affinity after phase 1 (simulating ectopic Eya expression in MBFCs after phase 1). **b**–**d** Individual morphological parameters of the simulation as a function of time. **b** Interface Angle. **c** Proportion of the germline-FC interface made up by the oocyte. **d**, Oocyte proportion of the germline. Phase boundaries were assigned to mid stage 7 and mid stage 10a. **e**, **f** Medial confocal sections of ECs expressing *gfp* (**e**) or *Eya* (**f**) under the control of *mirr-GAL4* (*mirr* > *gfp*, *mirr* > *eya*^OE^, MBFC driver), stained for E-Cad, F-Actin and nuclei (DAPI). Numbers denote germline areas in µm^2^. **g**, **h** Individual morphological parameters as a function of germline area for *mirr* > *gfp* and *mirr* > *eya*^*OE*^ ECs. **g** Interface Angle. **h** Proportion of the germline-FC interface made up by the oocyte. Dotted lines mark germline sizes at the transition between two phases. Bluegrey area marks 95% CI of the critical size (see Supplementary Fig. 5). All curves are LOESS fitted with a 95% CI area. n (*mirr* > *gfp*: 153 EC; *mirr* > *eya*^OE^: 157 EC). **i** Illustrations depicting phase 2 *mirr* > *gfp* and *mirr* > *eya*^*OE*^ egg chambers and the quantification of the nurse cell perimeter (light blue dotted line) proportion made up by the nurse cell-FC interface (dark blue line) for individual nurse cells in *mirr* > *gfp* and *mirr* > *eya*^*OE*^ ECs. Nurse cells are grouped by nurse cell row (A: anterior, MA: mid-anterior, MP: mid-posterior, P: posterior). Nurse cell row averages of ECs were analysed. Mean±SE, two-way Anova with Šídák’s multiple comparisons test, n (*mirr* > *gfp* (A: 8 ECs, MA: 6 ECs, MP: 8 ECs, P: 8 ECs), *mirr* > *eya*^*OE*^ (A: 7 ECs, MA: 9 ECs, MP: 9 ECs, P: 9 ECs)). **j** Oocyte proportion of the germline as a function of germline area for *mirr* > *gfp* and *mirr* > *eya*^*OE*^ ECs. Dotted lines mark germline sizes at the transition between two phases. Bluegrey area marks 95% CI of the critical size. All curves are LOESS fitted with a 95% CI area. n (*mirr* > *gfp*: 153 EC; *mirr* > *eya*^OE^: 157 EC). See Supplementary Table 7 for detailed statistical information. Source data are provided as a Source Data file.

Next, we hypothesized that the loss of Eya in MBFCs and the resultant switch to effective oocyte affinity drives oocyte expansion during phase 2. To test this, we simulated ectopic effective nurse cell affinity in the region of MBFCs after phase 1 (Fig. 8a, Supplementary Movie 9). As a consequence, the interface angle failed to decrease, and the oocyte failed to expand its contact with FCs and to increase in size during phase 2 (Fig. 8b–d). Experimentally, we forced MBFCs to ectopically express Eya during phase 2 using *mirr*-GAL4 (*mirr* > *eya*) (Fig. 8e, f). As predicted, ectopic Eya expression in MBFCs retained the interface angle above 90° representing a failure in switching to effective oocyte affinity (Fig. 8g, Supplementary Fig. 8a), once egg chambers had reached the critical size for mirr-GAL4-driven interference (Supplementary Fig. 5a-d). Accordingly, the oocyte failed to increase its interface with FCs, as posterior nurse cells outcompeted the oocyte for FC contact disrupting germline cluster organization (Fig. 8h, i). Ultimately, the ectopic effective nurse cell affinity resulted in a failure of oocyte growth (Fig. 8j, Supplementary Fig. 8b, c). Thus, the loss of Eya expression in MBFCs and the resultant switch to effective oocyte affinity is essential for oocyte growth and consequently egg chamber morphogenesis (Fig. 5g, h, Supplementary Fig. 5f–h).

### Creating an entirely Eya-negative follicle epithelium results in ectopic oocyte expansion

**Fig. 9:**
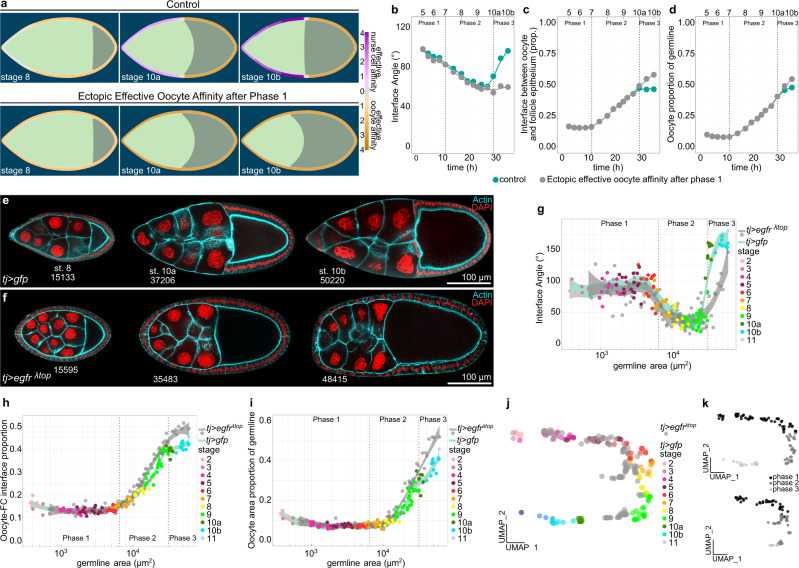
Inhibition of AFC differentiation causes ectopic oocyte affinity and oocyte expansion during phase 3. **a** Phase field model simulating germline cell behaviour as a function of their affinity for the follicle epithelium. wt affinity dynamics vs. ectopic effective oocyte affinity after phase 1 (simulating an Eya-negative follicle epithelium after phase 1). **b**–**d** Individual morphological parameters of the simulation as a function of time. **b** Interface Angle. **c** Proportion of the germline-FC interface made up by the oocyte. **d** Oocyte proportion of the germline. Phase boundaries were assigned to mid stage 7 and mid stage 10a. **e** Medial confocal sections of egg chambers expressing *CD8*-*tom* and *gfp* under the control of *tj-GAL4* (*tj* > *gfp*, FC driver), stained for F-Actin and nuclei (DAPI). Numbers denote germline areas in µm^2^. **f** Medial confocal sections of egg chambers expressing *CD8*-*tom* and *egfr*^λtop^ under the control of *tj-GAL4* (*tj* > *egfr*^λtop^, FC driver), stained for F-Actin and nuclei (DAPI). Numbers denote germline areas in µm^2^. **g–i** Individual morphological parameters as a function of germline area for *tj* > *gfp* and tj> *egfr*^λtop^ egg chambers. **g** Interface Angle. **h** Proportion of the germline-FC interface made up by the oocyte. **i** Oocyte proportion of the germline. Dotted lines mark germline sizes at the transition between two phases. Curves are LOESS fitted with a 95% CI area. n (*tj* > *gfp*: 119 ECs, *tj* > *egfr*^λtop^: 109 ECs). **j** UMAP comparing *tj* > *gfp* and *tj* > *egfr*^λtop^ egg chamber morphogenesis. **k** UMAP of *tj* > *gfp* and *tj* > *egfr*^λtop^ grouped into phase 1 (black, germline area<6500µm^2^), phase 2 (darkgrey, germline area >6500 µm^2^ & <31500 µm^2^), and phase 3 (lightgrey, germline area > 31500µm^2^). See Supplementary Table 7 for detailed statistical information. Source data are provided as a Source Data file.

**Fig. 10:**
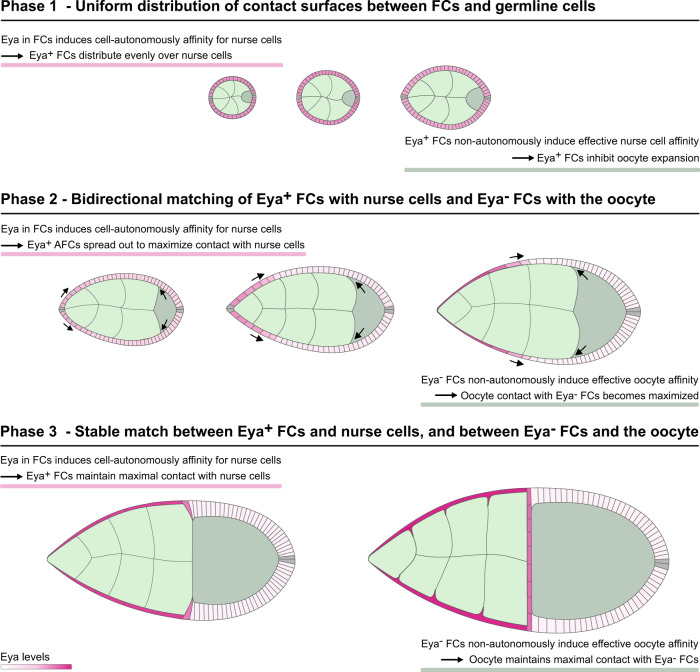
Eya-driven matching of FCs and germline cells. Illustration of Eya-controlled soma-germline coordination during *Drosophila* egg chamber morphogenesis. Eya in FCs cell-autonomously induces affinity for nurse cells, but not for the oocyte. This causes Eya-positive AFCs to spread out posteriorly to maximize their contact with nurse cells, which consequently displaces Eya-negative MBFCs onto the oocyte. Additionally, Eya in FCs non-autonomously controls effective germline affinity, such that Eya-positive FCs induce effective nurse cell affinity, while Eya-negative FCs induce effective oocyte affinity. As a result, the oocyte expands anteriorly during phase 2 to maximize its contact exclusively with Eya-negative FCs. Ultimately, these bilateral affinity dynamics result in a bidirectional matching dynamic that ensure robust matching of Eya-positive FCs with nurse cells and Eya-negative FCs with the oocyte. The established match represents the energetically preferred and therefore highly stable state.

We found that establishing the right match between germline cells and FCs by the end of phase 2 correlated with a switch back to effective nurse cell affinity and a cessation of relative oocyte expansion up until nurse cell dumping is initiated mid phase 3 (Fig. 6c–g). Of note, here we do not describe the total growth of the oocyte, but the relative expansion of the oocyte within the growing germline. We hypothesized that the match of the oocyte with all available Eya-negative FCs and the consequent positioning of Eya-positive FCs at the nurse cell–oocyte boundary at the transition from phase 2 to 3 causes the temporary halt in relative oocyte expansion. We thus tested if we could override this halt in relative oocyte growth by turning the entire epithelium Eya-negative. We simulated this by modelling effective oocyte affinity along the entire interface from phase 2 onwards. This caused the interface angle to remain smaller than 90° and resulted in a continuation of relative oocyte expansion after phase 2 (Fig. 9a–d, Supplementary Movie 10). In vivo, we expressed a constitutively active EGFR under the control of TJ-Gal4 (*tj* > *egfr*^*λtop*^) throughout the epithelium, which prohibited FCs at the anterior tip to adopt AFC fate^20^, consequently producing egg chambers with an Eya-negative follicle epithelium from phase 2 onwards (Fig. 9e, f). We found that these egg chambers retained their interface angles below 90° even after phase 2, reflecting prolonged effective oocyte affinity (Fig. 9g, Supplementary Fig. 8d). In line with that, the Eya-negative follicle epithelium failed to halt relative oocyte expansion at the end of phase 2 (Fig. 9h, i, Supplementary Fig. 8e, f), which caused a disruption of egg chamber morphogenesis (Fig. 9j, k, Supplementary Fig. 8g, h). Hence, the oocyte expands its contact exclusively with Eya-negative FCs and therefore halts its relative expansion once the right match is established at the end of phase 2. Thus, our data demonstrate that Eya in FCs non-cell autonomously controls the interaction of germline cells with the follicle epithelium, and thereby regulates oocyte growth dynamics. Taken together, Eya in FCs drives the matching of FCs and germline cells by controlling soma-germline coordination in a bilateral manner, giving rise to a robust matching mechanism (Fig. 10).

## Discussion

Our study demonstrates how germline and soma self-organize into functional units by using differential cell–cell affinity to match cell populations across cell lineages. We identify the co-transcriptional regulator Eya as the master regulator of this process. Our data demonstrate that Eya-expressing FCs cell-autonomously experience affinity towards nurse cells, but not towards the oocyte, while Eya-negative FCs experience affinity for neither. Additionally, we show that Eya in FCs regulates non-cell-autonomously the interaction of germline cells with the follicle epithelium, such that nurse cells experience effective affinity for Eya-positive FCs, whereas the oocyte experiences effective affinity for Eya-negative FCs. Moreover, our experiments demonstrate that these Eya-controlled bilateral affinity dynamics underlie the critical matching of FC subpopulations with germline cells.

Importantly, our phase field simulations provide a controlled environment to isolate the effects of changing affinity between cell lineages. We see that an affinity differential between cell types proportional to their relative Eya expression very closely recovers experimentally observed morphologies of both FCs and the germline. Furthermore, with parameters only adapted to wild-type models, the numerical simulations correctly predict significant morphological changes, such as FC-germline contact loss or premature oocyte expansion when affinities are suitably altered. This lends additional credence to our finding that Eya controlled differential affinity of cell lineages is underlying soma-germline matching.

Taken together, we demonstrate that Eya-controlled bilateral affinity dynamics at the soma-germline interface create a robust self-organizing system to drive the establishment of inter-lineage functional units. Thus, it represents a prime example of how differential cell–cell affinity can be utilized to drive complex morphogenetic events of multi-lineage tissues.

Yet, molecular and cellular details of Eya-dependent interactions remain unknown. A previous study revealed that MBFCs display high levels of apical-medial myosin when in contact with nurse cells and that MBFCs lose apical-medial myosin as soon as they encounter the oocyte^22^. While we show that Eya downregulation in MBFCs is sufficient to remove affinity for nurse cells and displace MBFCs onto the oocyte, the contact-dependent myosin enrichment suggests that Eya-negative FCs may not just lack affinity for nurse cells but experience active repulsion from the nurse cell surface. However, either mechanism will ensure reliable matching between Eya-negative MBFCs and the oocyte.

Furthermore, as we cannot experimentally separate the interaction of nurse cell or the oocyte with FCs, we introduced ‘effective affinities’ to characterize the interaction of germline cells with FCs in a relative manner. Consequently, we cannot distinguish whether effective oocyte affinity is the result of active cell-autonomous affinity of the oocyte for Eya-negative FCs or the consequence of a repulsion between nurse cells and Eya-negative FCs. However, both scenarios will result in the same effective affinity which would control oocyte expansion.

The most pressing question might be how differential affinity at the soma-germline interface is established at the molecular level. We expect that Eya regulates the expression of transmembrane receptors in FCs, which recognize nurse cell or oocyte-specific ligands or receptors. Subsequently, signaling downstream of these receptors must alter interfacial tensions by targeting the cytoskeleton and adhesion complexes in FCs and germline cells^2,3^.

## Methods

### *Drosophila* stocks and genetics

All experiments were performed on *Drosophila melanogaster*. Stocks (Supplementary Table 1) and experimental crosses (Supplementary Table 2) were maintained on standard fly food (10 L water, 74.5 g agar, 243 g dry yeast, 580 g corn meal, 552 mL molasses, 20.7 g Nipagin, 35 mL propionic acid) at 18 °C, 22 °C and 25 °C. For *mirr*-GAL4 and *fru*-GAL4 driven expression, newly hatched females were shifted to 27 °C or 30 °C for 48 h to relieve tub-GAL80-mediated repression of GAL4. Mosaic analysis was performed using the ‚flip-out’ and the mitotic FLP/FRT system^44^. For follicle epithelium clones, *flp* expression was induced in young adult females using a heat shock varying from 4 to 20 min depending on the *hsflp* construct (*hsflp [122]* vs. *hsflp[1]*) for ‚flip-out’ experiments, or 1 h using *hsflp [122]* for FRT/FLP experiments at 37 °C. Flies were fed yeast paste for 24 to 48 h prior to dissection.

### Immunohistochemistry and imaging

Ovaries, gonads and testis were dissected and fixed in 4% paraformaldehyde/PBS for 15 min at 22 °C. Washes were performed in PBS + 0.1% Triton X-100 (PBT). Samples were incubated with primary antibodies in PBT overnight at 4 °C: mouse anti-β-catenin (1:100, DSHB, N27A1), rat anti-E-cadherin (1:50, DSHB, DCAD2), rabbit anti-GFP (1:200, Thermo Fisher, G10362), rat anti-RFP (1:20, gift from H. Leonhardt, 5F8), mouse anti-β-gal (1:1000, Promega Z378B) mouse anti-Eya (1:100, DSHB eya10H6), rabbit anti-pMad (1:200, abcam ab52903). Ovaries were incubated with secondary antibodies for 2 h at 22 °C. DAPI (0.25 ng/μl, Sigma), Phalloidin (Alexa Fluor 488, Alexa Fluor 647 and Alexa Fluor 555, Molecular Probes, or Phalloidin-TRITC, Sigma) was used to visualize DNA and filamentous Actin. Following secondary antibodies were used: goat anti-mouse Alexa Fluor488 (Abcam, AB150117, 1:500), goat anti-rat Alexa Fluor488 (Abcam, AB150153, 1:500), goat anti-rabbit Alexa Fluor488 (Invitrogen, A11008, 1:500), donkey anti-mouse Alexa Fluor555 (Abcam, AB150110, 1:500), donkey anti-rat Alexa Fluor555 (Abcam, AB150154, 1:500), donkey anti-mouse Alexa Fluor647 (Abcam, AB150111, 1:500), donkey anti-rat Alexa Fluor647 (Abcam, AB150155, 1:500), goat anti-rabbit Alexa Fluor647 (Invitrogen, A21244, 1:500). Samples were mounted using Molecular Probes Antifade Reagents and imaged using Leica TCS SP8 confocal microscopes. Control and experimental samples were processed in parallel, and images were acquired using the same confocal settings.

### Image acquisition, analysis and quantification

Images were obtained with a LEICA TCS SP8 using the software LAS X. Images were processed in FIJI^45^ or MATLAB (R2021a, The Mathworks). Statistical analysis and generations of graphs were performed in R (R version 4.0.5), GraphPad (GraphPad Prism 9) and MATLAB (R2021a, The Mathworks).

### Quantification of Egg chamber morphology

Egg chamber morphology measurements were performed in FIJI, using the polygon, line, multi-point and angle tools. In total 24 parameters (Supplementary Table 3) were determined in 2D medial cross sections (through the anterior and posterior pole) for each genotype and its respective control (Supplementary Fig. 1b, Supplementary Table 4). Egg chambers were selected to sufficiently cover the entire developmental range and are therefore not representing frequencies of developmental stages or sizes. Wild type and control egg chambers were staged by previously described criteria^12,46^.

### Heatmap

Heatmaps were produced in R on standardized data. The wild type dataset was standardized independently, whereas datasets of genetically manipulated egg chambers were standardized in combination with their respective control dataset (i.e. from GAL4-driver backgrounds). Individual egg chambers were ordered by germline area size, smallest to largest from top to bottom. Egg chambers were assigned to their respective phase based on their germline area size. Additionally, all control egg chambers were assigned their respective stage according to classical staging criteria.

### UMAP Analysis

Analysis was performed in R (R version 4.0.5). Multidimensional morphology description datasets of all genotypes were pooled (*N* = 1026) and standardized. PCA was performed on the standardized dataset and the first 5 PCs (cumulative variance > 90%) were selected. Consecutively, uniform manifold approximation (UMAP) was applied with the following parameters: n_neighbors = 15, min_dist = 0.2^24^. UMAP analysis revealed three phases of egg chamber morphologenesis. Phase 1 was characterized by high UMAP_2 values and increasing UMAP_1 values, Phase 2 covered egg chambers with stable high UMAP_1 values and decreasing UMAP_2 values. Phase 3 consisted of egg chambers with low UMAP_2 levels and decreasing UMAP_1 levels. Germline size was increasing steadily along the trajectory allowing to assign egg chambers based on their germline size to their respective phase (Supplementary Table 5).

### Analysis of individual morphometric parameters as a function of germline area

Since classical egg chamber staging relies on morphology, it can only be reliably applied to wild type egg chambers but not to genetically manipulated egg chambers with disrupted morphology^22^. Moreover, classical staging is a method that leads to a discrete description of a continuous process. As live imaging of the entire egg chamber development is still impossible^47^, we were looking for a variable, as an alternative to temporal length, to identify developmental progression. Based on our previous work indicating that germline growth is to a large extent independent of follicle cell morphogenesis^22^, we decided that germline size would allow for a continuous and morphology-independent description of developmental progression. While we are aware of possible limitations to the assumption that the germline size increases independently, we are convinced that representing developmental progression by germline size is an advancement to classical staging and at this time point the best alternative to time.

Using germline size as a continuous independent variable allowed us to compare parameters as a function of germline area between control and manipulated egg chambers. Differences in curves of control and manipulated egg chambers were statistically compared by multiple linear regression with an interaction term.

1 $${{{{{\rm{Y}}}}}} \sim {{{{{\rm{germline}}}}}}\,{{{{{\rm{size}}}}}}+{{{{{\rm{genotype}}}}}}+{{{{{\rm{germline}}}}}}\,{{{{{\rm{size}}}}}}\,*\,{{{{{\rm{genotype}}}}}}$$

Since the interpretation of main effects is limited upon the existence of an interaction effect first p-values of the interaction term are stated. If the interaction effect *p* value ≥ 0.05 multiple linear regression without interaction was performed to analyze the main effect of the genotype.

2 $${{{{{\rm{Y}}}}}} \sim {{{{{\rm{germline}}}}}}\,{{{{{\rm{size}}}}}}+{{{{{\rm{genotype}}}}}}$$

Additionally, egg chambers were grouped into egg chambers with smaller germline areas than the critical size and egg chambers with larger germline areas than the critical size. Genotypes were compared by a two-way Anova and multiple comparisons testing. This analysis reduced the impact of the assumption that germline size was independent.

### Eya immunofluorescence intensity measurement

All images were processed in FIJI^45^. Images that were used for Eya fluorescence intensity measurements were acquired from the same sample, mounted on the same slide and imaged with identical settings. Eya was detected with an Alexa647-tagged secondary antibody to reduce auto-fluorescence interference. Maximum Intensity projections of a stack covering half of an egg chamber were used to quantify Eya intensities. Intensities were measured in FIJI with the polygon tool by creating nuclear (DAPI) ROIs and determining the mean Eya fluorescence intensity within each ROI.

### Analysis of apical area morphology

For the measurements of apical areas along the AP axis throughout development, we made use of a FIJI and Matlab pipeline to correct for 3D curvature in 2D images^48^. Egg chambers expressing *utrABD-gfp* and *cd8-tom* were used and stained for E-Cad to facilitate the segmentation of apical areas throughout egg chamber development. 3D confocal stacks of egg chambers were first projected on a 2D plane using the LocalZProjector Fiji plugin (ref. 49; https://gitlab.pasteur.fr/iah-public/localzprojector, v1.5.4). The LocalZProjector tool produces for each input stack the 2D projection of the tissue, restricted to include only voxels close to the tissue surface, and the height-map. The height-map is a 2D image where the pixel value stores the Z position of the tissue. The projected images were then segmented in Fiji, using the Tissue Analyzer^49^. There segmentation results were analyzed in a second step using DeProj (ref. 49; https://gitlab.pasteur.fr/iah-public/DeProj/) in MATLAB (R2021a, The Mathworks). Briefly, cells obtained from the 2D segmentation on projected images were mapped back onto the 3D surface of the tissue, obtained via the height-map generated during the projection step. From the apical contour of cells now in 3D, morphological parameters such as cell area, cell orientation and tissue slope were calculated and exported for subsequent analysis.

### Nurse cell morphology quantification

Individual nurse cell morphology was quantified in FIJI using the polygon tool. For each nurse cell a 2D-section was selected that cut through the center of the nucleus. In this section, the perimeter and area of the nurse cell, the length of the shared interface with FCs and the area of the nucleus (DAPI) were measured. Additionally, each nurse cell was assigned to a NC row within its egg chamber. Parameters were then used to calculate proportions and coefficients of variance (Supplementary Table 6).

### Morphological parameter quantification of simulations

Images representing the stages and the transition between stages were quantified in FIJI using the polygon, line, and angle tools. Measured data were plotted as a function of the reported duration of developmental stages^12,43^.

### Reporting summary

Further information on research design is available in the Nature Research Reporting Summary linked to this article.

## Supplementary information

Supplementary Information

Description of Additional Supplementary Files

Supplementary Movie 1

Supplementary Movie 2

Supplementary Movie 3

Supplementary Movie 4

Supplementary Movie 5

Supplementary Movie 6

Supplementary Movie 7

Supplementary Movie 8

Supplementary Movie 9

Supplementary Movie 10

Reporting Summary

## Data Availability

The data generated in this study are provided in the Source Data file. Source data are provided with this paper.

## Source data

Source Data

## Code availability

The C++ program code for the finite element phase field simulations is available from 10.5281/zenodo.7116639.
