# Supplementary material for: Eya-controlled affinity between cell lineages drives tissue self-organization during Drosophila oogenesis
Source: Nat Commun. 2022 Oct 26;13:6377. doi: 10.1038/s41467-022-33845-1 (PMC9605976; doi:10.1038/s41467-022-33845-1)
Supplement: Supplementary file 1 — Supplementary Information [file 41467_2022_33845_MOESM1_ESM.pdf]

Supplementary information

- Supplementary Figures 1-8
- Supplementary Note: Phase Field Model
- Supplementary Tables 1-7

Supplementary Figures 1-8

Figure S1

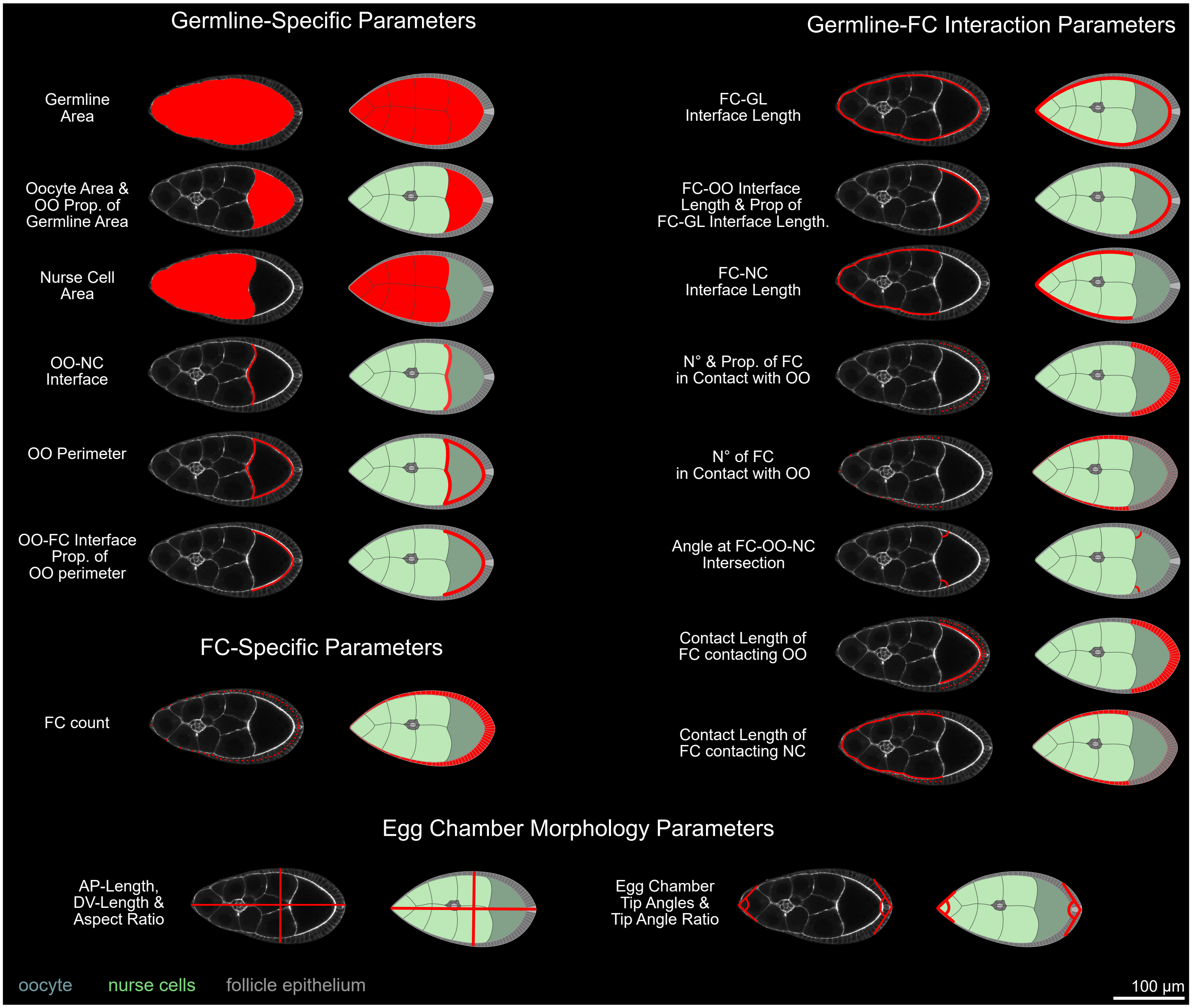

Germline-Specific Parameters
Germline Area
Oocyte Area & OO Prop. of Germline Area
Nurse Cell Area
OO-NC Interface
OO Perimeter
OO-FC Interface Prop. of OO perimeter
FC-Specific Parameters
FC count
Germline-FC Interaction Parameters
FC-GL Interface Length
FC-OO Interface Length & Prop of FC-GL Interface Length.
FC-NC Interface Length
N° & Prop. of FC in Contact with OO
N° of FC in Contact with OO
Angle at FC-OO-NC Intersection
Contact Length of FC contacting OO
Contact Length of FC contacting NC
Egg Chamber Morphology Parameters
AP-Length, DV-Length & Aspect Ratio
Egg Chamber Tip Angles & Tip Angle Ratio
oocyte
nurse cells
follicle epithelium
100 µm

Supplementary Figure 1

Drosophila egg chamber morphology parameters

Medial confocal sections and illustrations of phase 2 egg chambers visualizing parameters (red) that were quantified for the multidimensional morphology description.

Figure S2

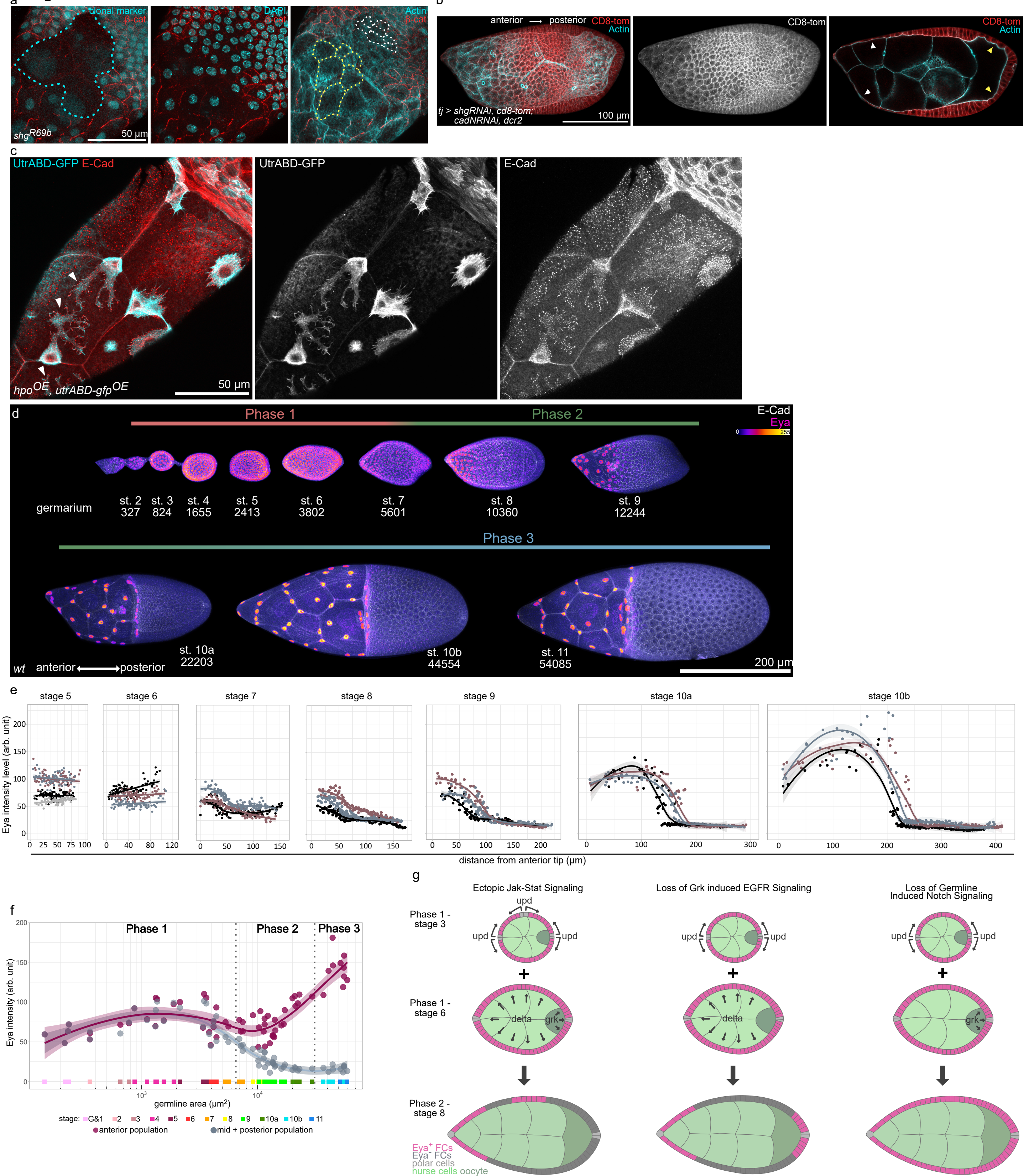
a
clonal marker β-cat
DAPI β-cat
Actin β-cat
shgR69b
50 µm
b
anterior → posterior
CD8-tom Actin
CD8-tom
tj > shgRNAi, cd8-tom, cadNRNAi, dcr2
100 µm
c
UtrABD-GFP E-Cad
UtrABD-GFP
E-Cad
hpoOE, utrABD-gfpOE
50 µm
d
Phase 1
Phase 2
E-Cad
Eya
germarium
st. 2 327
st. 3 824
st. 4 1655
st. 5 2413
st. 6 3802
st. 7 5601
st. 8 10360
st. 9 12244
Phase 3
st. 10a 22203
st. 10b 44554
st. 11 54085
wt anterior ←→ posterior
200 µm
e
stage 5
stage 6
stage 7
stage 8
stage 9
stage 10a
stage 10b
Eya intensity level (arb. unit)
distance from anterior tip (µm)
f
Phase 1
Phase 2
Phase 3
Eya intensity (arb. unit)
germline area (µm2)
stage: G&1 2 3 4 5 6 7 8 9 10a 10b 11
anterior population
mid + posterior population
g
Ectopic Jak-Stat Signaling
Loss of Grk induced EGFR Signaling
Loss of Germline Induced Notch Signaling
Phase 1 - stage 3
Phase 1 - stage 6
Phase 2 - stage 8
upd
delta
grk
Eya+ FCs
Eya- FCs
polar cells
nurse cells oocyte

Supplementary Figure 2

AFCs spread actively over the nurse cell surface

a, Maximum fluorescence intensity projection of a *shg*^{R69b} (null mutant) clone (cyan dotted outline) in a phase 2 (stage 9) egg chamber consisting of AFCs and MBFCs. Stained for β-cat, F-Actin and nuclei (DAPI). Apical surfaces of AFCs (yellow) and MBFCs (white) are outlined. E-Cad loss does not disrupt AFC flattening, and MBFCs maintain their comparatively small apical areas.
b, Maximum fluorescence intensity projection of an egg chamber expressing s*hg-RNAi, cadN-RNAi, CD8-tom* and *dcr2* under the control of *tj-GAL4* (FC driver), stained for F-Actin. Loss of E-Cad and N-Cad causes disruption of PFC morphology (yellow arrowheads), but not of AFC spreading (white arrowheads).
c, Maximum fluorescence intensity projection of a phase 3 egg chamber with clonal overexpression of *hippo (hpo)* and *utrABD-gfp*, stained for E-Cad. *hpo* overexpression leads to reduced cell volume. AFCs detach from each other but continue to spread out cell autonomously (white arrowheads point at protrusions).
d, Maximum fluorescence intensity projections of representative egg chambers covering all three morphological phases, stained for E-Cad and Eya. Fire LUT visualizes Eya levels in nuclei of FCs throughout egg chamber development. Numbers denote germline area in μm^2.
e, Nuclear Eya levels in FCs as a function of their distance to the anterior pole of egg chambers from stages 5 to 10b. Colours represent individual egg chambers at each stage. Colours do not relate between stages. n (st. 5: 4 EC, st. 6-10b: 3 EC). Curves are LOESS fitted with a 95% CI area.
f, Measured mean Eya intensities in anterior (maroon) and mid+posterior (grey) FC populations of egg chambers as a function of germline area. Coloured squares represent developmental stages of egg chambers. Curves are LOESS fitted with a 95% CI area. (n=66 egg chambers). See Supp. File S2 for detailed stage-wise statistical comparison.
g, Illustrations of genetic manipulations targeting fate determining factors and the result on Eya expression in FCs of phase 2.
See Supplementary Table 7 for detailed statistical information. Source data are provided as a Source Data file.

Figure S3

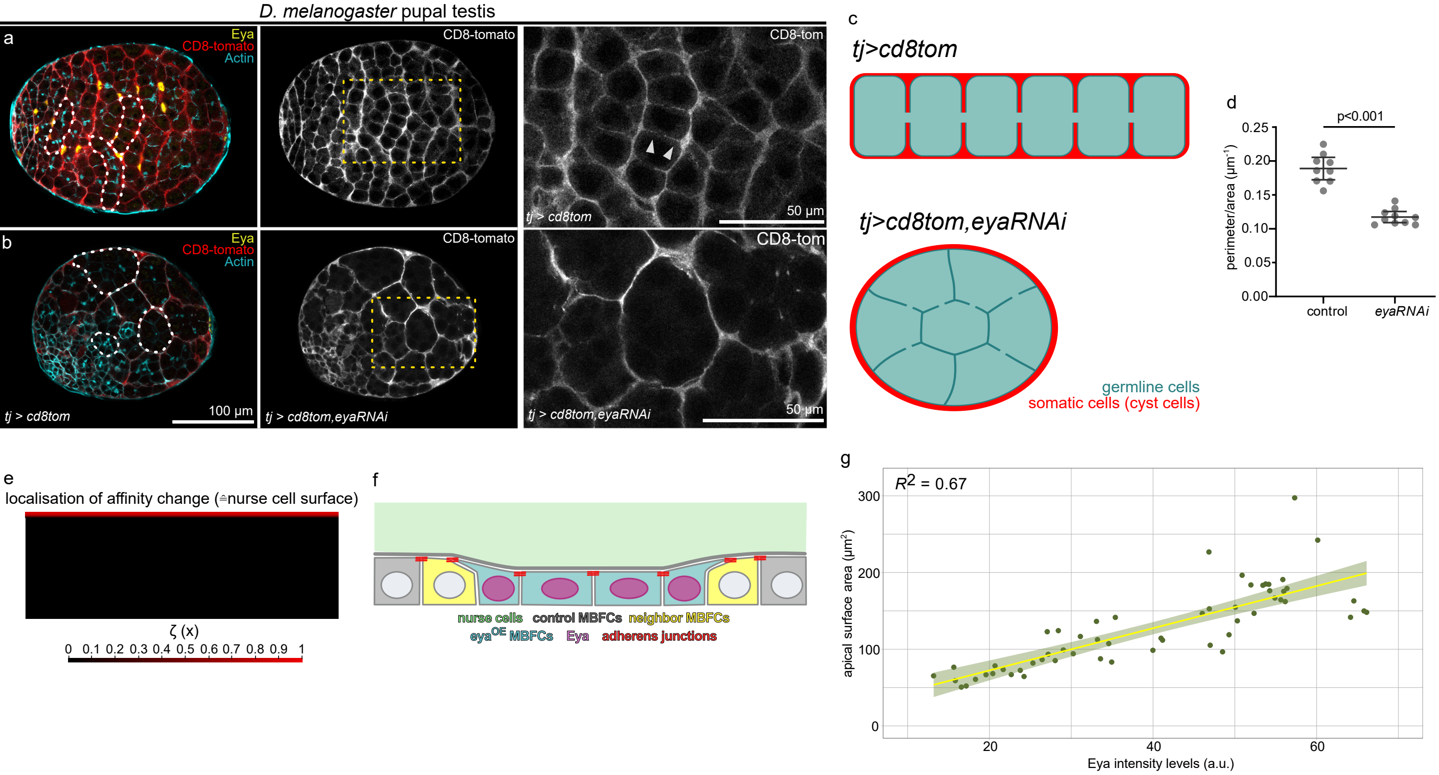

D. melanogaster pupal testis
a
Eya
CD8-tomato
Actin
CD8-tomato
CD8-tom
tj > cd8tom
50 µm
b
Eya
CD8-tomato
Actin
CD8-tomato
CD8-tom
tj > cd8tom
100 µm
tj > cd8tom,eyaRNAi
tj > cd8tom,eyaRNAi
50 µm
c
tj>cd8tom
tj>cd8tom,eyaRNAi
germline cells
somatic cells (cyst cells)
d
p<0.001
perimeter/area (µm⁻¹)
0.25
0.20
0.15
0.10
0.05
0.00
control
eyaRNAi
e
localisation of affinity change (≙nurse cell surface)
ζ (x)
0 0.1 0.2 0.3 0.4 0.5 0.6 0.7 0.8 0.9 1
f
nurse cells
control MBFCs
neighbor MBFCs
eya^OE MBFCs
Eya
adherens junctions
g
R2 = 0.67
apical surface area (µm2)
300
200
100
0
20
40
60
Eya intensity levels (a.u.)

Supplementary Figure 3

Maximization of the soma-germline interface in testis depends on Eya

a, Confocal sections of *D. melanogaster* pupal testis expressing *CD8-tom* under the control of *tj-GAL4* (*tj>CD8tom*, cyst cell driver). *CD8-tom* visualizes somatic cells (cyst cells) that envelope the developing germline. Somatic cells extend between individual germline cells and thereby maximize the soma-germline contact surface (white arrowheads). White dotted lines mark individual germline cysts. Yellow dotted rectangular marks position of enlarged area.
b, Confocal sections of *D. melanogaster* pupal testis expressing *CD8-tom* and *eya-RNAi* under the control of *tj-GAL4* (*tj>CD8tom,eya-RNAi,* cyst cell driver). *CD8-tom* visualizes somatic cells (cyst cells) that envelope the developing germline. Note that loss of Eya causes failure of cyst cells extending between individual germline cells and that the germline cyst adopts a spherical shape. Consequently, the contact surface between somatic cells and germline cells is minimized. White dotted lines mark individual germline cysts. Yellow dotted rectangular marks position of enlarged area.
c, Illustrations of *tj>CD8-tom* and *tj>CD8-tom,eya-RNAi* spermatogonial cysts.
d, Quantification of the ratio between the germline cyst interface in contact with somatic cells and germline area. Mean+95%CI, two-tailed unpaired Student's t-test, n (*tj>CD8-tom*: 9 cysts, *tj>CD8-tom,eya-RNAi*: 10 cysts).
e, Localization of the affinity change that represents the nurse cell surface.
f, Illustration of cell morphologies upon ectopic eya^{OE} expression in MBFC clones in contact with nurse cells during phase 2. Corresponds to Fig. 3m.
g, Linear regression between apical surface areas and Eya levels of FCs (FC from anterior rows 1-7 of stage 9 egg chambers, phase 2). Linear regression with 95% CI area, n= 57 AFCs from 3 EC.
See Supplementary Table 7 for detailed statistical information. Source data are provided as a Source Data file.

Figure S4

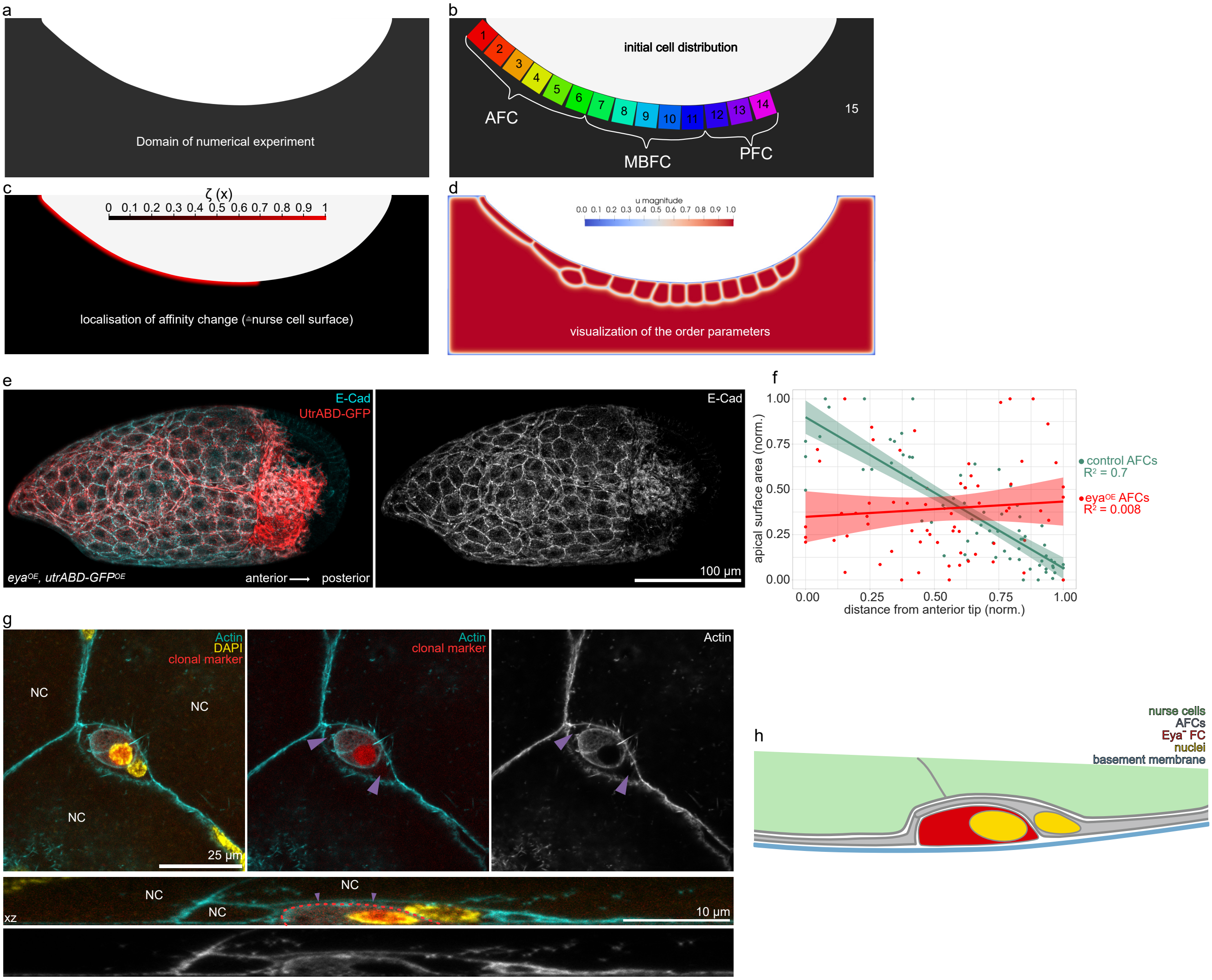
a
Domain of numerical experiment
b
initial cell distribution
1 2 3 4 5 6 7 8 9 10 11 12 13 14 15
AFC
MBFC
PFC
c
ζ (x)
0 0.1 0.2 0.3 0.4 0.5 0.6 0.7 0.8 0.9 1
localisation of affinity change (≙nurse cell surface)
d
u magnitude
0.0 0.1 0.2 0.3 0.4 0.5 0.6 0.7 0.8 0.9 1.0
visualization of the order parameters
e
E-Cad
UtrABD-GFP
eyaOE, utrABD-GFPOE
anterior ⟶ posterior
E-Cad
100 µm
f
apical surface area (norm.)
distance from anterior tip (norm.)
0.00 0.25 0.50 0.75 1.00
control AFCs
R² = 0.7
eyaOE AFCs
R² = 0.008
g
Actin
DAPI
clonal marker
NC
25 µm
Actin
clonal marker
Actin
xz
NC
10 µm
h
nurse cells
AFCs
Eya⁻ FC
nuclei
basement membrane

Supplementary Figure 4

Affinity-controlled FC interaction with the germline drives FC shapes and movements

a, Domain of computation Ω of the numerical experiment for the phase field model.
b, Initial cell distribution in the phase field model. Cells numbered from anterior to posterior.
c, Localization of the affinity change that represents the nurse cell surface.
d, Visualization of the order parameters of the phase field simulation.
e, Maximum fluorescence intensity projection of an egg chamber with clonal expression of *eya*^{OE} and *utrABD-gfp*, stained for E-Cad. Note how the gradient in apical surface areas of AFCs is lost upon broad overexpression of *eya*^{OE}.
f, Quantification of apical areas of AFCs as a function of their distance to the anterior tip for control AFCs and *eya*^{OE} AFCs. Linear regression with 95% CI area. n (control FCs: 71 FC, 3 EC; *eya*^{OE} FCs: 65 FC, 3 EC)
g, AFCs with clonal *eya-RNAi* expression of a phase 3 egg chamber, stained for F-Actin and nuclei (DAPI). One AFC is expressing *eya-RNAi* (red). *Eya-RNAi* expressing AFC (red dotted line) is rounded up and disconnected from nurse cells (NC). Wild type AFC extends between *eya-RNAi* AFC and nurse cells (purple arrowheads). Confocal section and xz-reslice shown.
h, Illustration of an AFC lacking Eya in a phase 3 egg chamber. The Eya-negative AFC is displaced from the nurse cell surface by wild type AFCs.
See Supplementary Table 7 for detailed statistical information. Source data are provided as a Source Data file.

Figure S5

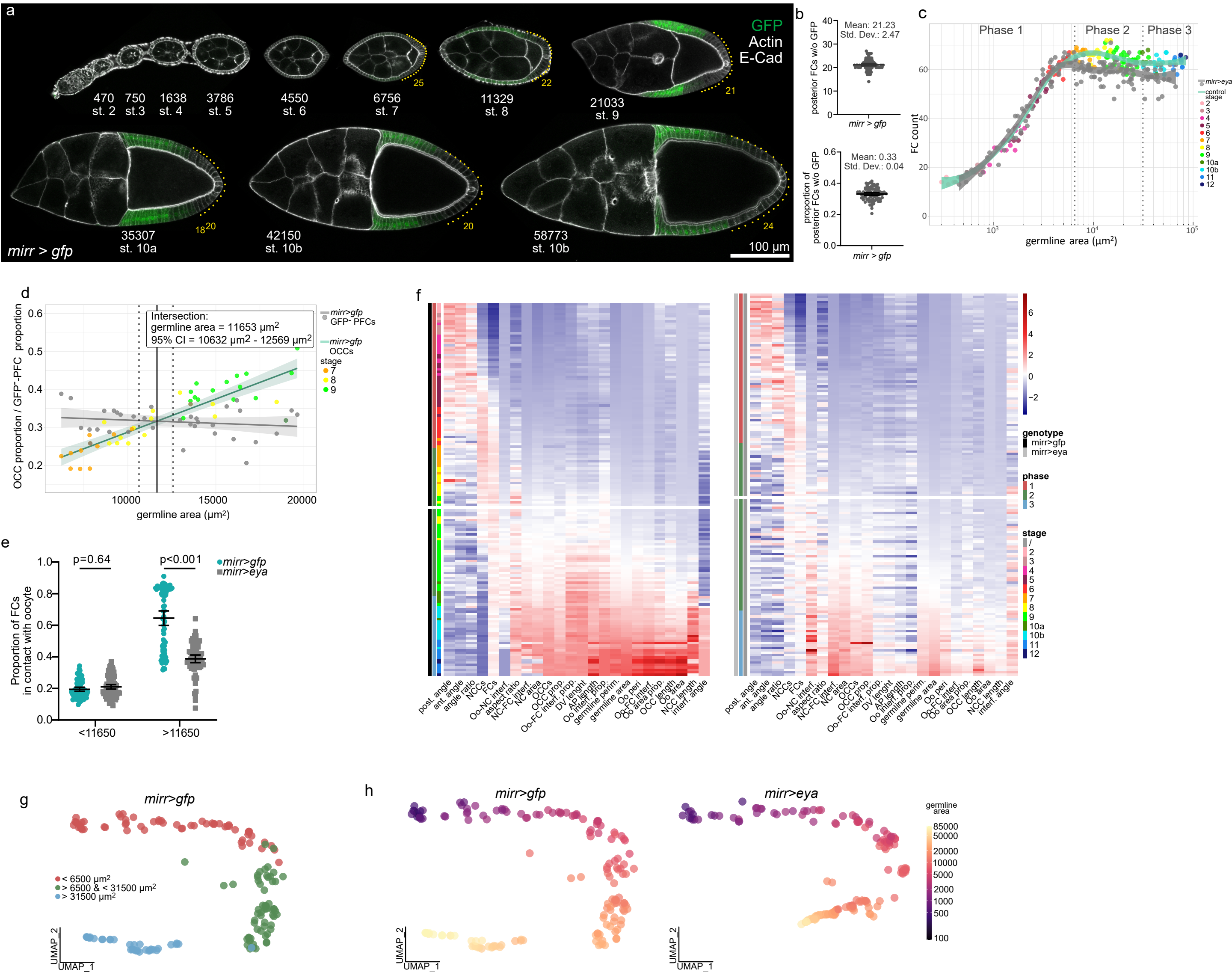

Supplementary Figure 5

Ectopic Eya expression in MBFCs during phase 2 inhibits MBFC transition onto the oocyte

a, Medial confocal sections of egg chambers expressing *gfp* under the control of *mirr-GAL4* (*mirr>gfp*, MBFC driver), stained for F-Actin and E-Cad. Yellow dots mark posterior cells that are not under the control of *mirr-GAL4*. Numbers denote germline areas in µm^2.
b, Quantification of posterior cells without GFP as total cell count and as proportion of all FCs. Mean+95% CI, n = 80 EC.
c, FC count as a function of germline area for *mirr>gfp* and *mirr>eya^{OE}* egg chambers. LOESS fitted curves with 95% CI area.
d, Determining the 'critical size' as the germline area at which first GFP-positive FCs are expected to come into contact with the oocyte. *mirr>gfp* egg chambers with germline areas > 6500 µm^2 and < 20000 µm^2 were used. Linear regression between the proportion of FCs in contact with the oocyte (OCC=oocyte contacting FCs) and the proportion of posterior FCs without GFP (GFP-negative PFCs) was performed. Crossing point of the two linear regression curves was fitted. Linear regression +95% CI area shown. Solid grey line marks germline area at estimated intersection point and dotted grey lines mark 95% CI of the intersection germline area. See Supp. File S2 for detailed statistical information.
e, Parameter comparison between *mirr>gfp* and *mirr>eya* egg chambers grouped by germline area into smaller and larger than critical size (11650 µm^2). Mean +95% CI, two-way Anova with Šídák's multiple comparisons test; n (*mirr>gfp* (<11650 µm^2): 74 EC, *mirr>gfp* (>11650 µm^2): 74 EC, *mirr> eya^{OE}* (<11650 µm^2): 86 EC, *mirr> eya^{OE}* (>11650 µm^2): 71 EC).
f, Heatmap of the 24 morphological parameters of *mirr>gfp* and *mirr>eya^{OE}* egg chambers. Each row represents an individual egg chamber with increasing germline areas from top to bottom. Break in heatmap marks critical size (11650 µm^2). n (*mirr>gfp*: 153 egg chambers, *mirr>eya*^{OE}: 157 egg chambers).
g, UMAP plot depicting *mirr>gfp* egg chambers coloured based on their phase affiliation.
h, UMAP plot of *mirr>gfp* and *mirr>eya^{OE}* egg chambers coloured by their germline area.
See Supplementary Table 7 for detailed statistical information. Source data are provided as a Source Data file.

Figure S6

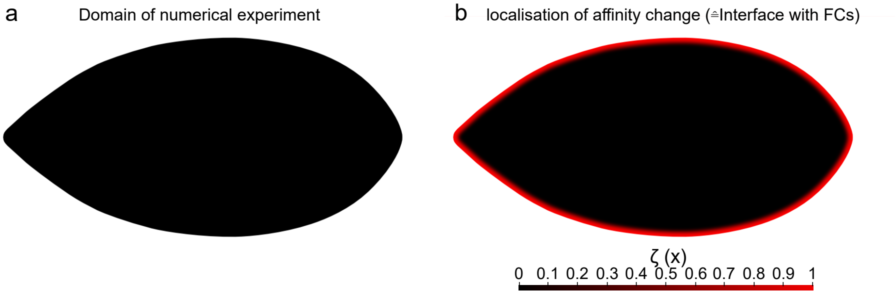
a
Domain of numerical experiment
b
localisation of affinity change (≙Interface with FCs)
ζ (x)
0 0.1 0.2 0.3 0.4 0.5 0.6 0.7 0.8 0.9 1

Supplementary Figure 6

Phase Field Model of Germline Cell Behaviour as a Function of their Effective Affinity for the Follicle Epithelium

a, Domain of computation Ω of the numerical experiment for the phase field model.
b, Localization of the affinity change that represents the nurse cell surface.

Figure S7

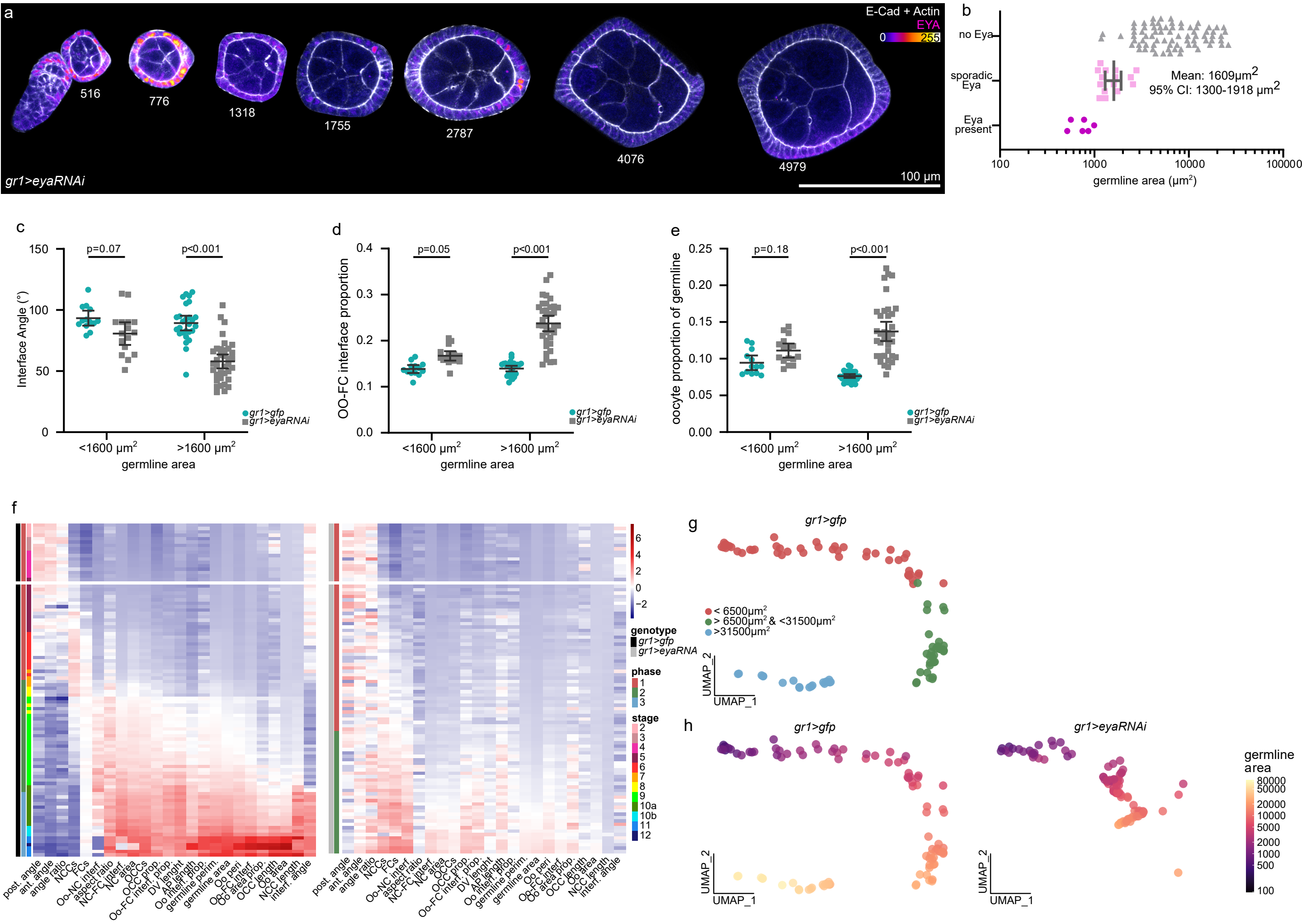

a
E-Cad + Actin
EYA
0
255
516
776
1318
1755
2787
4076
4979
gr1>eyaRNAi
100 µm
b
no Eya
sporadic Eya
Eya present
Mean: 1609µm2
95% CI: 1300-1918 µm2
100
1000
10000
100000
germline area (µm2)
c
Interface Angle (°)
p=0.07
p<0.001
gr1>gfp
gr1>eyaRNAi
<1600 µm2
>1600 µm2
germline area
d
OO-FC interface proportion
p=0.05
p<0.001
e
oocyte proportion of germline
p=0.18
p<0.001
f
post. angle
ant. angle
angle ratio
NCCs
FCs
Oo-NC interf.
aspect ratio
NC-FC interf.
NC area
OCCs
OCC prop.
Oo-FC interf. prop.
DV lenght
AP length
Oo interf. prop.
germline perim.
germline area
Oo peri
Oo-FC interf.
Oo area prop.
OCC length
Oo area
NCC length
interf. angle
genotype
gr1>gfp
gr1>eyaRNA
phase
1
2
3
stage
2
3
4
5
6
7
8
9
10a
10b
11
12
g
gr1>gfp
< 6500µm2
> 6500µm2 & <31500µm2
>31500µm2
UMAP_2
UMAP_1
h
gr1>gfp
gr1>eyaRNAi
germline area
80000
50000
20000
10000
5000
2000
1000
500
100

Supplementary Figure 7

Premature loss of Eya during phase 1 disrupts egg chamber morphogenesis

a, Medial confocal sections of egg chambers expressing *eya-RNAi* under the control of *gr1-GAL4* (*gr1>eya-RNAi*, FC driver), stained for F-Actin, E-Cad and Eya. Numbers denote germline area.
b, Quantification of Eya expression in FCs. Egg chambers were grouped into three categories (Eya present, sporadic Eya and no Eya in FCs) and plotted against their germline area. Mean+95%CI of the sporadic Eya group was determined as critical size from which on effects of *eya-RNAi* expression could be expected. Mean+95% CI, n (Eya present: 6 EC, sporadic Eya: 15 EC, no Eya: 75 EC).
c,d,e, Parameter comparison between *gr1>gfp* and *gr1>eya-RNAi* egg chambers for phase 1 (germline area < 6500 μm^2). Egg chambers grouped into smaller and larger than critical germline area (1600 μm^2). **c,** Interface Angle. **d,** Oocyte-FC interface proportion of germline-FC interface. **e,** Oocyte area proportion of germline area. Mean+95% CI, two-way Anova with Šídák's multiple comparisons test; n (*gr1>gfp* (<1600 μm^2): 13 EC, *gr1>gfp* (>1600 μm^2): 27 EC, *gr1>eya-RNAi* (<1600 μm^2): 16 EC, *gr1>eya-RNAi* (>1600 μm^2): 36 EC).
f, Heatmap of the 24 morphological parameters of *gr1>gfp* and *gr1>eya-RNAi* egg chambers. Each row represents an individual egg chamber with increasing germline areas from top to bottom. Note that no egg chambers of *gr1>eya-RNAi* exist with germline sizes corresponding to phase 3, as they degenerate before. Break in heatmap marks critical size (1600 μm^2). n (*gr1>gfp*: 97 EC, *gr1>eya-RNAi*: 96 EC).
g, UMAP plot of *gr1>gfp* egg chambers coloured based on their phase affiliation.
h, UMAP plot of *gr1>gfp* and *gr1>eya-RNAi* egg chambers coloured by their germline area size.
See Supplementary Table 7 for detailed statistical information. Source data are provided as a Source Data file.

Figure S8

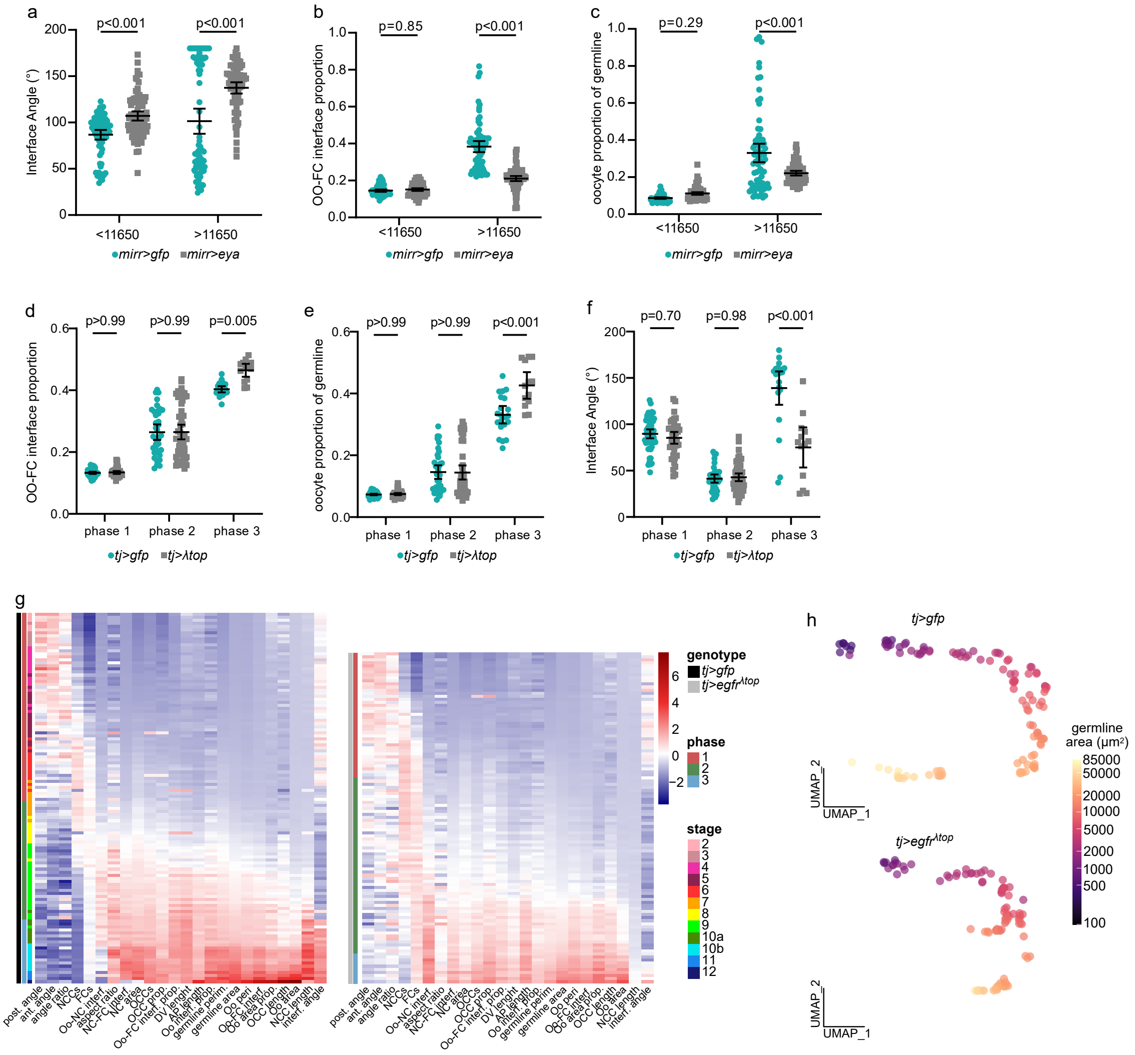

a
p<0.001
p<0.001
Interface Angle (°)
<11650
>11650
mirr>gfp
mirr>eya
b
p=0.85
p<0.001
OO-FC interface proportion
<11650
>11650
mirr>gfp
mirr>eya
c
p=0.29
p<0.001
oocyte proportion of germline
<11650
>11650
mirr>gfp
mirr>eya
d
p>0.99
p>0.99
p=0.005
OO-FC interface proportion
phase 1
phase 2
phase 3
tj>gfp
tj>λtop
e
p>0.99
p>0.99
p<0.001
oocyte proportion of germline
phase 1
phase 2
phase 3
tj>gfp
tj>λtop
f
p=0.70
p=0.98
p<0.001
Interface Angle (°)
phase 1
phase 2
phase 3
tj>gfp
tj>λtop
g
genotype
tj>gfp
tj>egfrλtop
phase
1
2
3
stage
2
3
4
5
6
7
8
9
10a
10b
11
12
post. angle
ant. angle
angle ratio
NCCs
FCs
Oo-NC interf.
aspect ratio
NC-FC interf.
NC area
OCCs
OCC prop.
Oo-FC interf. prop.
DV length
AP length
Oo interf. prop.
germline perim.
germline area
Oo peri
Oo-FC interf.
Oo area prop.
OCC length
Oo area
NCC length
interf. angle
h
tj>gfp
tj>egfrλtop
UMAP_1
UMAP_2
germline area (μm²)
85000
50000
20000
10000
5000
2000
1000
500
100

Supplementary Figure 8

Manipulating Eya expression patterns during phase 2 and 3 disrupts oocyte expansion dynamics

a,b,c, Parameter comparison between *mirr>gfp* and *mirr>eya^{OE}* egg chambers. Egg chambers grouped into smaller and larger than critical germline area (11650 µm^2). **a,** Interface Angle. **b,** Oocyte-FC interface proportion of germline-FC interface. **c,** Oocyte area proportion of germline area. Mean+95% CI, two-way Anova with Šídák's multiple comparisons test; n (*mirr>gfp* (<11650 µm^2): 74 EC, *mirr>gfp* (>11650 µm^2): 74 EC, *mirr>eya^{OE}* (<11650 µm^2): 86 EC, *mirr>eya^{OE}* (>11650 µm^2): 71 EC).

d,e,f Parameter comparison between *tj>gfp* and *tj>egfr$^{\lambda top}$* egg chambers. Egg chambers subdivided by phases. **d,** Interface Angle. **e,** Oocyte-FC interface proportion of germline-FC interface. **f,** Oocyte area proportion of germline area. Mean+95% CI, two-way Anova with Šídák's multiple comparisons test; n (*tj>gfp* (phase 1): 62 EC, *tj>gfp* (phase 2): 39 EC, *tj>gfp* (phase 3): 21 EC, *tj>egfr$^{\lambda top}$* (phase 1): 41 EC, *tj>egfr$^{\lambda top}$* (phase 2): 58 EC, *tj>egfr$^{\lambda top}$* (phase 3): 10 EC). See Supp. File S2 for detailed statistical information.

g, Heatmap of the 24 morphological parameters of *tj>gfp* and *tj>egfr$^{\lambda top}$* egg chambers. Each row represents an individual egg chamber with increasing germline areas from top to bottom. n (*tj>gfp*: 122 EC, *tj>egfr$^{\lambda top}$*: 109 EC).

h, UMAP plot of *tj>gfp* and *tj>egfr$^{\lambda top}$* egg chambers coloured based on germline area size.

See Supplementary Table 7 for detailed statistical information. Source data are provided as a Source Data file.

Supplementary Note: Phase Field Model

Based on classical work[1], we formulated a phase field model to describe the collective behavior of follicle cells (FCs) – or, in a separate simulation, the behavior of the oocyte and nurse cells – as their relative affinity to the germline surface or the FC surface, respectively, changes. Nomoura[2] used phase fields to describe multicellular systems. Moure and Gomez[3] provide a recent overview of the application of phase field models to study the migration of individual cells and their collective behavior. Wenzel and Voigt[4] consider collective cell migration. Phase field models have furthermore been successfully applied in, e.g., tumor growth modeling[5,6]. The ideas therein are similar to the use of such models for grain boundary evolution in polycrystalline materials[7,8]. In the spirit of Modica and Mortola[9], we consider the phase field model as a diffuse approximation for a sharp interface model, where each cell is subject to a surface tension, exhibits a prescribed affinity to a defined part of the boundary, and must satisfy a number of constraints.

Set-up of the model. Roughly following work for grain boundary evolution[7], we start with an energy of the form

$$F(u, x, t) = \int_\Omega f(u_1(x), u_2(x), \dots, u_N(x), x, t) + \sum_{j=1}^{N} \frac{\kappa_j}{2} |\nabla u_j|^2 \, \mathrm{d}x, \tag{1}$$

associated to a vector valued order parameter field $u = (u_1, u_2, \dots, u_N)$ defined on a domain $\Omega \subset \mathbb{R}^2$, describing N cells, where the function f is given as

$$f(u, x, t) = \sum_{j=1}^{N} \phi_j(x, t) \left(-\frac{\alpha}{2} u_j^2 + \frac{\beta}{4} u_j^2 \right) \tag{2}$$

$$+ \gamma \sum_{j=1}^{N} \sum_{i=j+1}^{N} u_i^2 u_j^2 \tag{3}$$

for constants $\kappa_j > 0$, $\alpha > 0$, $\beta > 0$, $\gamma > 0$ and functions $\phi_j(x, t)$. It should be noted that, as long as $2\gamma > \phi_j(x, t)\beta$, the function f is locally minimized exactly at the "pure phases" $u_j = \pm 1$, $u_i = 0$ if $i \neq j$. For ϕ_j independent of j, these minimizers are degenerate, otherwise minimizers for which the corresponding ϕ_j is larger are energetically favored. Each one of these pure phases denotes the position of a given cell, i.e., the set $\{u \approx (0, \dots, 0, 1, 0, \dots, 0)\}$, with the entry 1 in the j-th component of the vector is the part of the domain Ω occupied by cell number j. Our experiments are conducted such that the negative minima are not seen by the simulation.

In our phase field simulation, we compute the L^2-gradient flow of the time dependent energy F, thus solving

$$\mu \dot{u}_j = -\phi_j(x, t) \left(-\alpha u_j + \beta u_j^3 \right) - 2\gamma \left(\sum_{i \neq j} u_i^2 \right) u_j + \kappa_j \Delta u_j, \tag{4}$$

given an initial condition $u^{(0)}(x) = \left(u_1^{(0)}(x), \dots, u_N^{(0)}(x) \right)$ describing the starting arrangement of cells and boundary conditions $u(\cdot, t) = 0$ on $\partial\Omega$ for all $t \geq 0$. Above, $\dot{u}_j$ denotes the time derivative of u_j and Δ is the Laplace-operator. The constant $\mu > 0$ denotes the time-scale. For a subset

Table S7: Polynomial coefficients for $\phi_j(t) = \sum_{k=0}^{6} a_{jk} t^k$.

	a_{j0}	a_{j1}	a_{j2}	a_{j3}	a_{j4}	a_{j5}	a_{j6}
Row $j = 1$	7.22519	0	-77.5944	279.897	-398.117	276.264	-78.7263
Row $j = 2$	6.99944	0	-95.0403	458.222	-905.773	836.279	-290.315
Row $j = 3$	6.82173	0	-65.7434	295.514	-588.111	576.878	-214.334
Row $j = 4$	6.48935	0	-47.8079	243.145	-605.621	707.857	-291.604
Row $j = 5$	7.02846	0	-96.7535	521.399	-1246.33	1351.44	-523.595
Row $j = 6$	6.65126	0	-24.4169	-5.49423	91.6108	-72.3666	10.2843
Row $j = 7$	6.79833	0	-79.8118	304.779	-528.733	433.352	-134.256

of cells we constrain this gradient flow to be area preserving, i.e.,

$$\int_\Omega u_j(x,t)\,\mathrm{d}x = \int_\Omega u_j^{(0)}(x)\,\mathrm{d}x \quad \text{for all } t > 0 \text{ and } j \in J_{\mathrm{vol}} \subset \{1,\ldots,N\}, \tag{5}$$

where J_{vol} simply denotes the subset of cells for which volume preservation is enabled.

Follicle cells simulation. The numerical experiments are performed on a domain resembling the exterior of the germline at stage 10a, see Fig. S4a (in the supplementary materials), which is the rectangle $(-2,2) \times (0,1.5)$ minus the space occupied by the germline. The initial cell distribution is given as seen in Fig. S4b, where (from left-to-right), the individual colors denote individual cells (cells 1 through 14).

In our simulation, cells 1–6 represent AFCs, where cell 6 is a centripetal cell. Cells 7–11 are MBFCs, and 12–14 PFCs. We reduced the number of FCs since MBFCs and PFCs share similar affinity dynamics.

The gray background is assigned to "cell" $N = 15$. On the area occupied by cell j we then simply set $u_j^{(0)} = 1$, zero outside that area. Area preservation during the evolution is enforced for cells 1 through 14, but not for cell 15 (for which it is automatically preserved as the remaining area of Ω). For all experiments, we first relax the initial condition by simulating the evolution equation with the initial $\phi_j(x,0)$ until an equilibrium was reached.

To simulate the changing affinity of the individual FCs' apical surface to the nurse cells, we increase the function $\phi_j(x,t)$ in the vicinity of the nurse cells' surface – this makes it energetically favorable for cell j to concentrate its volume near the apical surface. More precisely, we fix a function $\zeta(x) \in [0,1]$ on Ω, as seen in Fig. S4c and set $\phi_j(x,t) = \hat{\phi}_j(t)\zeta(x) + 2(1-\zeta(x))$. In the sense of a sharp interface limit, this change near the boundary should be seen as an affinity changing boundary condition for the phase field variables.

In all experiments, the time-scale μ, which fixes the relaxation time relative to the time-scale on which $\hat{\phi}_j(t)$ changes, is set to $\mu = 1.1125 \cdot 10^{-3}$ hours. The further constants are $\alpha = \beta = 1$, $\gamma = 20$ and $\kappa_j = 3 \cdot 10^{-4}$ for $j \neq 15$, and $\kappa_{15} = 6 \cdot 10^{-4}$.

For the experiment recreating the wild-type situation, we fix $\hat{\phi}_j(t)$ for cells $j = 1$–7 (from left to right) corresponding to rows 1–7 in Fig. 4d. All other cells are set to the affinity of row 7, up to cell 15. The affinities were generated from averaged measured EYA levels (normalized to lie between 2 and 12, shown as circles in Fig. 4c) using least squares approximation by a sixth-order polynomial $\phi_j(t) = \sum_{k=0}^{6} a_{jk} t^k$ constrained to have vanishing derivative at $t = 0$ and $t = 36$ hours. The coefficients can be found in Table S7.

A number of further numerical simulations with 15 cells, corresponding to experiments, were conducted.

- For the experiment with equal affinities we set the affinities $\hat{\phi}_j(t)$ of all cells 1–15 constant to that of row 1 at $t = 0$.
- Simulating the behavior with absent affinity gradient in AFCs, we set the affinity for cells 1–6 to that of row 1. Cells 7–15 receive the affinity of row 7. The time-dependent behaviors of $\hat{\phi}_1(t)$ and $\hat{\phi}_7(t)$ are not changed here.
- In the experiment where the third cell is assigned a lower affinity, the setting is equal to the wild-type experiment, except that cell 3 is assigned the lower affinity of row 7.
- To simulate the ectopic affinity increase in MBFCs, we assign affinities of row 1–5 to AFCs 1–5 as usual. Centripetal cell 6, as well as MBFCs 7 and 8, however, receive the increased affinity of row 2. All other cells receive the affinity of row 7, as usual.

Rectangular domain. To further examine the shape of adjacent cells, an experiment with three square cells on a rectangle $(0, 1.5) \times (0, 0.5)$ was performed. All parameters were chosen as in the above described simulations. On the rectangle, we set $\zeta(x) = \left(1 - \frac{x_2}{10}\right)^+ = \max\left\{0, 1 - \frac{x_2}{10}\right\}$. For the experiment showing the polarization of cell motion, we set $\hat{\phi}_j(t)$ for the three cells to the affinities of rows 5, 6, and 7, from left to right. For the experiment with symmetric affinities we used rows 7, 5, and again 7.

Oocyte and nurse cells. Finally, the interior of the egg chamber, i.e., the oocyte and the nurse cells, were simulated on a suitable domain (essentially the complement of the domain for the FCs simulation, see Fig. S6a). The nurse cells were considered collectively as one "cell", $j = 1$, and the oocyte is cell $j = 2$ (thus a total of $N = 2$ cells). In this experiment, we set $\kappa_j = 5 \cdot 10^{-4}$ for all j. The initial condition was set such that 84% of the total length of the domain is covered by the nurse cells - this initial condition is allowed to relax before the start of stage 5. Again, the affinity towards the surface of the germline was modulated again by making the functions ϕ_j space- and time dependent. For this experiment, we chose

$$\phi_1(x, t) = \zeta(x)\hat{\phi}_1(x, t), \tag{6}$$

$$\phi_2(x, t) = \zeta(x)\hat{\phi}_2(x, t) + p + p_{\text{constr}}(v), \tag{7}$$

where ζ (shown in Fig. S6b) is again the cutoff function to ensure the changed affinity is only applied at the surface. Furthermore, we add a constant pressure $p = 6.5 \cdot 10^{-2}$ and an oocyte volume v dependent pressure $p_{\text{constr}}(v) = \frac{7 \cdot 10^{-3}}{v - 7 \cdot 10^{-2}}$ to the oocyte model, noting that the total area of the computational domain is 3.96. These terms model oocyte growth effects due to transport of matter by the nurse cells as well as a minimal volume cutoff for the oocyte.

The now space- and time-dependent functions $\hat{\phi}_j(x, t)$ are given as follows. First, the relative affinity $\iota(x, t)$ of FCs at different points on the boundary is computed by evaluating which cell row $j = 1, \ldots, 7$ is occupying which point on the boundary, and then evaluating the respective relative affinity of that cell row by the polynomial expression as in the previous experiment. The centers of the cell positions are measured by experiment and then fit using a cubic polynomial with coefficients given in Table S8, where a position of 0 denotes the anterior end of the germline and 1 denotes the posterior end. The positions and measured values are illustrated in Fig. 6 j&k, and we assume that each cell occupies the space up to the mid point between itself and its neighbor (or up to the ends for the first and the last cell). We remark that "cell" 7 again encompasses a number of cells towards the posterior end, which all have comparable Eya expression. From the relative affinity we then compute the oocyte and nurse cell affinity as

Table S8: Polynomial coefficients for the cell position centers $\sum_{k=0}^{5} b_{jk} t^k$.

	b_{j0}	b_{j1}	b_{j2}	b_{j3}	b_{j4}	b_{j5}
Row $j = 1$	0.069791	0	-0.63461	3.28734	-4.76849	2.09623
Row $j = 2$	0.128197	0	-2.09907	8.93208	-11.334	4.54757
Row $j = 3$	0.189402	0	-2.93928	11.7103	-14.0911	5.4224
Row $j = 4$	0.224447	0	-2.83186	11.2882	-13.2071	4.92549
Row $j = 5$	0.261511	0	-2.69858	10.2531	-11.154	3.85078
Row $j = 6$	0.313222	0	-2.98772	10.8291	-11.3885	3.80847
Row $j = 7$	0.361412	0	-3.41095	12.3623	-13.3566	4.63224

$\hat{\phi}_1(x,t) = 2.0 + 0.7(\imath(x,t) - 5.7120)^+$ and $\hat{\phi}_2(x,t) = 2.0 + 0.7(5.7120 - \imath(x,t))^+$, respectively, where the cutoff of 5.7120 corresponds to an Eya fluorescence intensity of 72. Again, the superscript "+" denotes taking the positive part of a term. Note that in figures we simply show the function $0.7(5.7120 - \imath(x,t))$, so that positive values indicate increased oocyte affinity, and negative values indicate increased nurse cell affinity. Fig. 6j shows for which FC row at what time nurse cell and oocyte contact are preferred, respectively. The transitions between constant affinities were smoothed by an affine transition layer of width 0.05.

One further change compared to the simulations of the exterior of the germline concerns the change of volume of the involved cells. Here, the volumes of the nurse cells and the oocyte are not fixed, but the in each time step only a fraction of $8 \cdot 10^{-4}$ of the unconstrained volume change for the oocyte is allowed, simulating the limited volume exchange through ring canals. This is implemented by first solving the unconstrained problem and then constraining the new volume using a Lagrange multiplier.

Again, a number of further simulations, corresponding to experiments, were conducted.

- Oocyte affinity was set high on the entire surface starting already from stage 5. This recapitulates a premature loss of Eya during phase 1.
- Nurse cell affinity was set high on 80% of the surface, and oocyte affinity is high only on 20% of the surface starting stage 6. This simulated ectopic effective nurse cell affinity in the region of MBFCs.
- Oocyte affinity was increased on the entire surface from stage 6, creating an entirely Eya-negative follicle epithelium during phase 2.

Further simulation and visualization details. All numerical experiments were conducted using the finite element method. For the exterior of the germline, Ω was discretized using P1 finite elements on a uniform tesselation with 92 321 triangles (for a domain volume of 4.03), on the rectangular domain we used 68 887 triangles (for a domain volume of 0.75) and for the interior of the germline Ω was discretized using 62 968 triangles (for a domain volume of 3.96). The time-discretization was semi-implicit with a time-step size of $5.625 \cdot 10^{-5}$ hours. For visualization, we show the sets $u_j \geq 0.3$, $j = 1, \dots, 14$ (or $j = 1, \dots, 3$ for the rectangular domain), smoothly cut off and colored by the respective cell's affinity. Each (multi-threaded) simulation requires between 6 hours and two days of wall time (depending on the number of phase field variables considered) on an Intel Xeon Gold 6230. For visualization of the interior of the germline, the nurse cells and the oocyte were colored in light and dark green, respectively, with the affinity visualized by the color in a band around the germline. For an example of a more direct visualization of the order parameters, see Fig. S4d, where the value $\left(\sum_{j=1}^{N} u_j^2\right)^{\frac{1}{2}}$

is plotted for the experiment with modified cell 3 affinity. For creating the images, the software Paraview 5.10.1[10,11] was used.

Supplementary Tables 1-7

Supplementary Table 1 - Fly strains

Fly line	Chromosome	Source
w[118]	*X*	David Bilder
hsflp[1]	*I*	BDSC 6
hsflp[122]	*I*	Iswar Hariharan
act>y[+]>-GAL4,UAS-RFP/TM6c	*III*	BDSC 30558
act>y[+]>-GAL4,UAS-GFP/TM6b	*I,III*	Bruce A. Edgar
FRT42D ubi-eGFP/CyO	*I,II*	BDSC 5626
Act5C.GAL4 (FRT.CD2), UASp-UtrABD-eGFP /TM6c	*III*	BDSC 4780 & Katja Röper (recombined in this study)
tubGAL80[ts]-20; TM2/TM6b	*II,III*	BDSC 7019
Sco/CyO; tub-GAL80[ts]-7	*II,III*	BDSC 7018
c306-GAL4	*X*	BDSC 3743
tj-GAL4, Mef2-GAL80/CyO	*II*	Sally Horne-Badovinac
tj-GAL4, UAS-CD8tom/CyO; UAS-dcr2/TM6C	*II,III*	David Bilder
MTD-GAL4 (Otu-Gal4::VP16;nos-GAL4;nos-GAL4::VP16)	*I,II,III*	BDSC 31777
mirr-GAL4/TM3, Sb[1]	*III*	BDSC 29650
tub-GAL80[ts]-20/Cyo; fru-GAL4/TM6(hu)	*II,III*	Vincent Mirouse
matalpha-GAL-VP16	*III*	BDSC 7063
GR1-Gal4	*III*	BDSC 36287
PG150-GAL4/FM7a	*I*	Kim McCall
UASp-UtrABD-eGFP	*III*	Thomas Lecuit
UAS-upd1 / CyO,ubi-GFP	*II*	Martin Zeidler
UAS-Dl RNAi (GL000520)	*II*	BDSC 36784
UAS-hts-mCherry	*III*	BDSC 66171
UAS-eya	*III*	BDSC 5675

UAS-eya RNAi (HMS04515)	*II*	BDSC 57314
FRT42D shg [R69b]/ SM6b, cn#1	*II*	Ulrich Tepass
UAS-shg RNAi (HMS00693)	*III*	BDSC 32904
UAS-shg RNAi (GL00646)	*II*	BDSC 38207
UAS-CadN RNAi (1093/GD)	*II*	VDRC1093
UAS-hpo	*II*	Barry Thompson
UAS-GFP (S56T) /CyO	*II*	BDSC 1521
UAS-mCD8::RFP	*II*	BDSC 32219
UAS-egfr$^{\lambda top}$	*III*	BDSC 59843

Supplementary Table 2 - Experimental genotypes

Figure	Genotype
**Figure 1**	
a - g	*w[118]*
**Figure S1**	
b	*w[118]*
**Figure 2**	
c - f	*tj-GAL4, UAS-CD8tom/+ ; UAS-dcr2/UASp-UtrABD-eGFP*
g,h	*w[118]*
j	*hsflp[122] /+; UAS-upd1/Sp; act>y[+]>-GAL4,UAS-GFP/+*
k	*grk(2B6) b, cn, slbo/grk(2E12)*
l	*Otu-Gal4::VP16/+;nos-GAL4/UAS-Dl RNAi;nos-GAL4::VP16/+*
**Figure S2**	
a	*hsflp [122]/+; FRT42D ubi-eGFP/FRT42D-shg[R69b]*
b	*tj-GAL4, UAS-CD8tom /UAS-CadNRNAi; UAS-shg RNAi/UAS-Dcr2*
c	*hsflp [122] /+;UAS-hpo/+; act> y[+]>Gal4, UASp-UtrABD-eGFP/+*
d-f	*w[118]*
**Figure 3**	
a,b,g,h,l,m	*hsflp [122] /+;; act> y[+]>Gal4, UASp-UtrABD-eGFP /UAS-eya*
d,e	*hsflp [122] /+;; act> y[+]>Gal4, UASp-UtrABD-eGFP /UAS-eya RNAi*
j,o	*hsflp [122] /+;; act> y[+]>Gal4, UASp-UtrABD-eGFP /UAS-hts-mCherry*
p,q	*Tj-Gal4, mef2-Gal80/+ ; UAS-hts-mCherry/+*
r,u	*w[118]*
**Figure S3**	
b	*tj-GAL4, UAS-CD8tom/CyO; UAS-dcr2/TM6C*
c	*tj-GAL4, UAS-CD8tom/UAS-eyaRNAi; UAS-dcr2/+*
e	*tj-GAL4, UAS-CD8tom/CyO; UAS-dcr2/TM6C & tj-GAL4, UAS-CD8tom/UAS-eyaRNAi; UAS-dcr2/+*
**Figure 4**	
a, c, l	*w[118]*
b, e	*tj-GAL4, UAS-CD8tom/+ ; UAS-dcr2/UASp-UtrABD-eGFP*
i	*C306-GAL4/+; tub-GAL80[ts]-20/UAS-eya RNAi*
**Figure S4**	

f, g	*hsflp [122] /+;; act> y[+]>Gal4, UASp-UtrABD-eGFP /UAS-eya*
h	*hsflp[122]/+;UAS-eya RNAi/+; act>y[+]>-GAL4,UAS-RFP/+*
j	*tj-Gal4, mef2-Gal80/+ ; UAS-htsmCherry/+*
k	*w[118]*
l	*hsflp [122]/+; FRT42D ubi-eGFP/FRT42D-shg[R69b]*
**Figure 5**	
b,d	*tub-GAL80[ts]-20/ UAS-GFP (S56T); mirr-GAL4/+*
c,e	*tub-GAL80[ts]-20/ +; mirr-GAL4/UAS-eya*
f,g,h	*tub-GAL80[ts]-20/ UAS-GFP (S56T); mirr-GAL4/+ & tub-GAL80[ts]-20/ +; mirr-GAL4/UAS-eya*
**Figure S5**	
a, b, d, f	*tub-GAL80[ts]-20/ UAS-GFP (S56T); mirr-GAL4/+*
c, e, g, h	*tub-GAL80[ts]-20/ UAS-GFP (S56T); mirr-GAL4/+ & tub-GAL80[ts]-20/ +; mirr-GAL4/UAS-eya*
**Figure 6**	
c-k	*w[118]*
**Figure 7**	
e	*GR1-Gal4/ UAS-GFP (S56T)*
f	*GR1-Gal4/ UAS-eya RNAi*
g,i-n	*GR1-Gal4/ UAS-GFP (S56T) & GR1-Gal4/ UAS-eya RNAi*
**Figure S7**	
a, b	*GR1-Gal4/ UAS-eya RNAi*
d	*GR1-Gal4/ UAS-GFP (S56T)*
c, e, f, g, h	*GR1-Gal4/ UAS-GFP (S56T) (control) & GR1-Gal4/ UAS-eya RNAi*
**Figure 8**	
e	*tub-GAL80[ts]-20/ UAS-GFP (S56T); mirr-GAL4/+*
f	*tub-GAL80[ts]-20/ +; mirr-GAL4/UAS-eya*
g-j	*tub-GAL80[ts]-20/ UAS-GFP (S56T); mirr-GAL4/+ & tub-GAL80[ts]-20/ +; mirr-GAL4/UAS-eya*
**Figure S8**	
a - c	*tub-GAL80[ts]-20/ UAS-GFP (S56T); mirr-GAL4/+ & tub-GAL80[ts]-20/ +; mirr-GAL4/UAS-eya*
d - h	*tj-GAL4, UAS-CD8tom/UAS-GFP(S56T); UAS-dcr2/+ & tj-GAL4, UAS-CD8tom/+; UAS-dcr2/UAS-egfr*$^{\lambda top}$
**Figure 9**	

e	*tj-GAL4, UAS-CD8tom/UAS-GFP(S56T); UAS-dcr2/+*
f	*tj-GAL4, UAS-CD8tom/+; UAS-dcr2/UAS-egfr*$^{\lambda top}$
g-k	*tj-GAL4, UAS-CD8tom/UAS-GFP(S56T); UAS-dcr2/+ & tj-GAL4, UAS-CD8tom/+; UAS-dcr2/UAS-egfr*$^{\lambda top}$

Supplementary Table 3 - Morphological parameters

**Parameters**	**unit**	**Parameters**	**unit**
germline area	µm^2	FC in contact with oocyte (OCC)	count
oocyte area	µm^2	FC in contact with nurse cells (NCC)	count
nurse cell area	µm^2	total no. of FCs	count
oocyte proportion of germline (oocyte area/germline area)	-	OCC proportion (OCC/total FC)	-
FC-OO interface length	µm	av. OCC length	µm
OO-NC interface length	µm	av. NCC length	µm
FC-NC interface length	µm	AP axis length	µm
FC-OO interface proportion of FC interface (FC-OO interface length/ germline-FC interface length)	-	DV axis length	µm
OO perimeter	µm	angle of anterior egg chamber tip	°
germline-FC interface length	µm	angle of posterior egg chamber tip	°
FC-OO interface proportion of OO perimeter (FC-OO interface length/ OO perimeter)	-	aspect ratio (AP axis length/DV axis length)	-
angle at FC-OO-NC meeting point	°	tip angle ratio (angle of anterior egg chamber tip/ angle of posterior egg chamber tip)	-

Supplementary Table 4 - Genotypes and number of egg chambers quantified for UMAP analysis

genotype	N	genotype	N
Wild type (w^{1118})	126	*fru>gfp*	85
gr1>gfp	97	*fru>eyaRNAi*	81
gr1>eyaRNAi	96	*tj>gfp,cd8tom,dcr2*	122
mirr>gfp	153	*tj>cd8tom,dcr2,*$egfr^{\lambda top}$	109
mirr>eya	157		

Supplementary Table 5 - Germline area size bins for Phase 1, 2, 3

Phase	Germline size (µm^2)
1	≤ 6500
2	> 6500 & ≤ 31500
3	>31500

Supplementary Table 6 - Quantification parameters for morphology of individual nurse cells

NC morphology parameter	unit
NC area	µm^2
NC perimeter	µm
NC-FC interface length	µm
Nucleus area	µm^2
NC-FC interface proportion (NC-FC interface / NC perimeter)	-
NC area proportion (NC area / germline area)	-
Nucleus proportion (Nucleus area / NC area)	-

Supplementary Table 7 - Reproducibility and Statistics

Reproducibility Statement			
Wherever representative microscopy images are shown at least 5 fully independent experiments were performed (including independent genetic crosses).			
**Figure 1 f,g,h**			
**Statistical Analysis**	Local polynomial regression fitting (LOESS), descriptive statistics and linear regression with y ~ germline size		
**Data**	Genotype: *wt* (w^{1118})<br>Multidimensional egg chamber morphology dataset. Parameters plotted against germline size.<br>Germline Sizes for phase assignment:<br>Phase 1: <6500µm^2; Phase 2: >6500µm^2 & < 31500 µm^2; Phase 3: >31500 µm^2		
**N**	126 egg chambers manually selected to sufficiently cover egg chamber development from stage 2 to 12. Frequencies are not representative of population stage or size frequencies.<br>Phase 1: 62, Phase 2: 39, Phase 3: 26		
**Descriptive Statistics**	Total cell count	OCC proportion	NC area
**LOESS**	Degree of polynomial: 2<br>Level of CI: 0.95<br>Size of neighbourhood for fitting: 0.5	Degree of polynomial: 2<br>Level of CI: 0.95<br>Size of neighbourhood for fitting: 0.4	Degree of polynomial: 2<br>Level of CI: 0.95<br>Size of neighbourhood for fitting: 0.25
**Phase 1**	Min: 13<br>Max: 66<br>Mean: 46.68<br>SD: 15.03<br>CV: 0.322	Min: 0.107<br>Max: 0.258<br>Mean: 0.1732<br>SD: 0.035<br>CV: 0.2	Min: 306<br>Max: 5375<br>Mean: 2652<br>SD: 1477<br>CV: 0.56
**Phase 2**	Min: 57<br>Max: 69<br>Mean: 61.82<br>SD: 2.752<br>CV: 0.045	Min: 0.2188<br>Max: 0.825<br>Mean: 0.4917<br>SD: 0.184<br>CV: 0.37	Min: 6043<br>Max: 20556<br>Mean: 10872<br>SD: 3570<br>CV: 0.33
**Phase 3**	Min: 52<br>Max: 64<br>Mean: 58.19<br>SD: 2.743<br>CV: 0.047	Min: 0.7869<br>Max: 0.907<br>Mean: 0.844<br>SD: 0.032<br>CV: 0.038	Min: 5214<br>Max: 32757<br>Mean: 21603<br>SD: 8557<br>CV: 0.4
**Linear Regression**	Phase 1 Slope: $8.5*10^{-3}$<br>Phase 2 Slope: $-1.5*10^{-4}$<br>Phase 3 Slope: $-5.53*10^{-5}$	Phase 1 Slope: $1.03*10^{-5}$<br>Phase 2 Slope: $2.96*10^{-5}$<br>Phase 3 Slope: $1.43*10^{-6}$	Phase 1 Slope: 0.9<br>Phase 2 Slope: 0.59<br>Phase 3 Slope: -0.38

Figure 2 f

**Statistical Analysis**	Local polynomial regression fitting (LOESS)		
**Data**	Genotype: *tj>cd8tom,ubdABD-gfp*<br>Nuclear Eya levels and apical surface areas of cells plotted as a function of their distance to the anterior pole.		
	Phase 1 (st. 4)	Phase 2 (st. 9)	Phase 3 (st. 10a&b)
**N**	2 egg chambers, 45 cells	3 egg chambers, 192 cells	3 egg chambers, 112 cells<br>Subgrouped into high (intensity >50) Eya cells and low (intensity <50) Eya cells
**Local polynomial regression fitting parameters**	Degree of polynomial: 2<br>Level of CI: 0.95<br>Size of neighbourhood for fitting: 0.75	Degree of polynomial: 2<br>Level of CI: 0.95<br>Size of neighbourhood for fitting: 0.6	Degree of polynomial: 2<br>Level of CI: 0.95<br>Size of neighbourhood for fitting: 0.75

Figure 2g

**Statistical Analysis**	Descriptive Analysis		
**Data**	Genotype: w^{1118}<br>FC row count of rows in contact with nurse cells of individual egg chambers sub-grouped into phase 1, 2 and 3.<br>For Phase 1 only egg chambers with completed FC proliferation were used.		
	Phase 1	Phase 2	Phase 3
**N**	11	32	12
**Descriptive Statistics**	Min: 20<br>Max: 23<br>Mean: 21.82<br>SD: 1.4<br>CV: 0.064	Min: 8<br>Max: 21<br>Mean: 15.81<br>SD: 3.5<br>CV: 0.222	Min: 6<br>Max: 7<br>Mean: 6.08<br>SD: 0.289<br>CV: 0.047

Figure 2h

**Statistical Analysis**	Descriptive Statistics					
**Data**	Genotype: w^{1118}<br>Mean Eya row intensities of Phase 3 (stage 10a and 10b) egg chambers.					
**N**	5 egg chambers					
**Descriptive Statistics**	Row 1<br>Min: 69.46<br>Max: 112.4<br>Mean: 92.66<br>SD: 17.95	Row 2<br>Min: 77.10<br>Max: 127.8<br>Mean: 101<br>SD: 18.98	Row 3<br>Min: 91.29<br>Max: 133.9<br>Mean: 105.8<br>SD: 16.88	Row 4<br>Min: 92.2<br>Max: 150<br>Mean: 114.4<br>SD: 21.66	Row 5<br>Min: 92.37<br>Max: 151.9<br>Mean: 115.4<br>SD: 22.14	Row 6<br>Min: 48.16<br>Max: 67.30<br>Mean: 62.40<br>SD: 8
	Row 7<br>Min: 16.39<br>Max: 23.14<br>Mean: 20.09<br>SD: 2.85	Pooled rows 8-26:<br>Min: 6.27<br>Max: 23.47<br>Mean: 13.05<br>SD: 3.4				

Figure S2e									
**Statistical Analysis**	Regression fitting								
**Data**	Genotype: w^{1118}<br>Nuclear Eya levels FCs along the anterior-posterior axis<br>Each data point represents a single cell.								
	Stage 5	Stage 6	Stage 7	Stage 8	Stage 9	Stage 10a	Stage 10a	Stage 10b	Stage 11
**N**	4	3	3	3	3	3	3	3	3
**Regression**	Linear	Linear	LOESS	LOESS	LOESS	LOESS	LOESS	LOESS	LOESS

Figure S2f			
**Statistical Analysis**	Descriptive Statistics and local polynomial regression fitting (LOESS)		
**Data**	Genotype: w^{1118}<br>Nuclear Eya levels in anterior and mid+posterior FC populations.<br>Each intensity value corresponds to the mean of a FC population of one egg chamber.<br>To determine FC population mean intensities at least 10 nuclei were quantified.		
**LOESS**	Anterior population<br>Degree of polynomial: 2<br>Level of CI: 0.95<br>Size of neighbourhood for fitting: 0.5	Mid + posterior population<br>Degree of polynomial: 2<br>Level of CI: 0.95<br>Size of neighbourhood for fitting: 0.5	
**N**	66 egg chambers		
	Phase 1:	Phase 2	Phase 3
**N**	27	28	11
**Descriptive Statistics Descriptive Statistics**	anterior population		
	Min: 45.88<br>Max: 110.2<br>Mean: 76.09<br>SD: 18.58<br>CV: 0.24	Min: 43.44<br>Max: 109.5<br>Mean: 73.91<br>SD: 18.47<br>CV: 0.25	Min: 106.9<br>Max: 181.0<br>Mean: 137.4<br>SD: 21.93<br>CV: 0.16
	posterior population		
	Min: 44.95<br>Max: 101.3<br>Mean: 72.24<br>SD: 17.44<br>CV: 0.24	Min: 11.1<br>Max: 53.8<br>Mean: 26.39<br>SD: 11.41<br>CV: 0.43	Min: 11.51<br>Max: 21..49<br>Mean: 16.41<br>SD: 3.266<br>CV: 0.20

Figure S2f									
**Statistical Analysis**	RM two-way Anova with Šídák's multiple comparisons test								
**Data**	Genotype: w^{1118}<br>Nuclear Eya levels in anterior and mid+posterior FC populations.<br>Each intensity value corresponds to the mean of a FC population of one egg chamber.<br>To determine FC population mean intensities at least 10 nuclei were quantified.								
**N**	Stage 4	Stage 5	Stage 6	Stage 7	Stage 8	Stage 9	Stage 10a	Stage 10b	Stage 11
**Anterior population**	7	5	5	5	4	17	5	5	5
**Mid + posterior population**	7	5	5	5	4	17	5	5	5

**F-Statistics**		
	Stage x intensity	F = 75.74 on 8 and 49 DF, p < 0.001
	Stage	F = 12.27 on 8 and 49 DF, p < 0.001
	intensity	F = 693.4 on 8 and 49 DF, p < 0.001

	Egg chamber				F = 3.44 on 8 and 49 DF, p < 0.001				
**Descriptive Statistics**	Stage 4	Stage 5	Stage 6	Stage 7	Stage 8	Stage 9	Stage 10a	Stage 10b	Stage 11
	Anterior population								
**Mean**	89.1	89.2	67.8	69.6	66.2	70.2	103	141.5	139.5
**SD**	14.1	22.5	18.3	6.6	17.3	16.4	6	26.2	16.1
	Mid + posterior population								
**Mean**	86.4	84.3	64.9	48.6	35.7	23.4	15.4	17.2	15.6
**SD**	12.2	18.7	16.2	7.1	9.6	7.9	1.1	3.8	3.3
**Multiple Comparison**	Stage 4	Stage 5	Stage 6	Stage 7	Stage 8	Stage 9	Stage 10a	Stage 10b	Stage 11
**t**	0.5373	0.8264	0.5	3.564	4.645	14.69	14.9	21.17	21.1
**DF**	49								
**P**	> 0.99	> 0.99	> 0.99	0.007	< 0.001	< 0.001	< 0.001	< 0.001	< 0.001

**Figure 3b**		
**Statistical Analysis**	Descriptive Statistics and two-tailed Welch's t-test	
**Data**	Genotype: *hsflp;; act>>UAS-ubdGFP/UAS-eya*<br>Phase 2 egg chambers with clonal ectopic *eya* expression.<br>Cells grouped into MBFCs ectopically expressing *eya* (eya^+) and control MBFCs (control). Apical surface areas were measured.	
**N**	3 egg chambers of similar germline size with several eya^+ clones	
	control: 71	*eya*$^+$: 64
**Descriptive Statistics**	Mean: 56.87<br>SD: 18.71	Mean: 117.7<br>SD: 45.36
**Two-tailed Welch's t-test**	t = 11.6 with 113.1 DF, p< 0.001	
**Figure 3e**		
**Statistical Analysis**	Descriptive Statistics and two-tailed Welch's t-test	
**Data**	Genotype: *hsflp; UAS-eyaRNAi; act>>Gal4, UAS-rfp*<br>Apical surface areas of control AFCs and AFCs expressing *eyaRNA*i during Phase 2 (stage 9).	
**N**	3 egg chambers of similar germline size with several *eyaRNAi* expressing clones	
	control: 20	*eyaRNAi:* 20
**Descriptive Statistics**	Mean: 287<br>SD: 133.7	Mean: 64.73<br>SD: 21.74
**Two-tailed Welch's t-test**	t = 7.339 with 20 D, p < 0.001	
**Figure 3h**		
**Statistical Analysis**	Descriptive Statistics and unpaired Student's t-test	
**Data**	Genotype: *hsflp;; act>>UAS-ubdGFP/UAS-eya*<br>Phase 2 egg chambers with clonal ectopic *eya* expression.<br>Cells grouped into MBFCs ectopically expressing *eya* (eya^+) and control MBFCs (control). Apical surface areas were measured.	
**N**	3 egg chambers of similar germline size with several eya^+ clones	
	control: 75	*eya*$^+$: 83
**Descriptive Statistics**	Mean: 26.1<br>SD: 6.5	Mean: 27.5<br>SD: 8.1
**Unpaired t-test**	t = 1.166 with 156 DF, p=0.245	
**Figure 3m**		
**Statistical Analysis**	Welch one-way Anova with Dunnett's T3 Multiple Comparisons	
**Data**	Genotype: *hsflp;; act>>UAS-ubdGFP/UAS-eya*<br>Ratio of apical vs lateral section areas in unaffected MBFCs (control), MBFCs ectopically expressing eya and in contact with eya^- cells (eya^+) and MBFCs in direct contact with eya expressing MBFCs (neighbors).	
**N**	FCs of 4 different egg chambers.<br>Control MBFCs: 16 eya^+ MBFCs : 14 neighbours: 18	
**W**	W=61.24 on 2 and 20.52 DF, p < 0.001	
**Multiple Comparison**	Control MBFC vs eya^+ MBFC: t=7.936 with 24.15 DF, p < 0.001<br>Control MBFC vs neighbour: t=2.812 with 23.74 DF, p=0.02	
**Figure 3r**		
**Statistical Analysis**	Descriptive Statistics and Unpaired Student's t-test	
**Data**	Genotype: *w^{1115}*<br>Angle between anterior lateral surface and germline surface of AFC and MBFC during Phase 2 (stage 9). Angles of cells of 4 egg chambers were measured.	
**N**	AFC: 45	MBFC: 82
**Descriptive Statistics**	AFC: Mean: 38.88<br>SD: 14.71	MBFC: Mean: 90.05<br>SD: 16.15
**Two-tailed t-test**	t = 17.61 with 125 DF, p < 0.001	

<table>
<tr><th colspan="3">Figure S3d</th></tr>
<tr><td>Statistical Analysis</td><td colspan="2">Two-tailed unpaired Student’s t-test</td></tr>
<tr><td>Data</td><td colspan="2">Genotypes: tj>cd8tom & tj>cd8tom,eyaRNAi
Ratio of perimeter shared with somatic cells (cd8-tom) vs area for individual germline cysts</td></tr>
<tr><td>N</td><td colspan="2">tj>cd8tom : 2 testis, 9 cysts
tj>cd8tom,eyaRNAi: 5 testis, 10 cysts
Only cysts of sizes >1000µm^2 and <1500µm^2 were used to determine perimeter/area ratios</td></tr>
<tr><td>Descriptive statistics</td><td>tj>cd8tom: mean = 0.1889, SE = 0.0072</td><td>tj>cd8tom,eyaRNAi: mean = 0.1173, SE = 0.00363</td></tr>
<tr><td>Two-tailed unpaired t-test</td><td colspan="2">Two-tailed, t = 9.165 with 17 DF, p < 0.001</td></tr>
<tr><th colspan="3">Figure S3g</th></tr>
<tr><td>Statistical Analysis</td><td colspan="2">Linear Regression</td></tr>
<tr><td>Data</td><td colspan="2">Genotype: tj>cd8tom,ubdABD-gfp
Eya Levels (a.u.) and apical surface areas (µm^2) of FCs from anterior rows 1-7 of stage 9 egg chambers with similar germline sizes.</td></tr>
<tr><td>N</td><td colspan="2">57 FCs, 3 egg chambers</td></tr>
<tr><td>Linear Regression</td><td colspan="2">y= 17+2.7*x (y=apical surface area, x=Eya levels)
R^2=0.67</td></tr>
</table>

Figure 4c	
**Statistical Analysis**	Descriptive Statistics
**Data**	Genotype: *w^{1118}*<br>Mean Eya fluorescence intensities of first 7 anterior rows of egg chambers from stage 5-10b.<br>Eya row intensities as a function of time. Time derived from reported stage durations [12,13]<br>Eya mean intensities of stages were assigned to the midpoint of each stage. Assigned relative affinities are shown.
**N**	stage 5: 5, stage 6: 5 , stage 7: 5 , stage 8: 4 , stage 9e: 8 , stage 9m: 9 , stage 10a: 5 , stage 10b: 5
**Polynomial Regression**	For each row dynamic a least squares approximation by sixth-order polynomial constrained to have vanishing derivative at t = 0 and t = 36 hours was generated. See table S7 for coefficients.
**Figure 4e**	
**Statistical Analysis**	Descriptive Statistics
**Data**	genotype: *tj>cd8tom,ubdABD-gfp*<br>Apical surface areas of first 7 anterior rows for stage 7,8,9e,9m & 10 (pooled stage 10a and 10b) egg chambers
**N**	Stage 7: 4, stage 8: 3, stage 9e: 5, stage 9m: 5, stage 10: 3
**LOESS**	Discrete independent variable, therefore LOESS fitted curve just for visualization of gradient development.
**Figure 4f**	
**Statistical Analysis**	Descriptive Statistics
**Data**	Apical length measurements of the results of the phase field model describing the collective behaviour of FCs as a function of their affinity.<br>Apical length were measured and squared to generate approximations of apical surface areas of the first 7 anterior rows for stage 7-10a.
**LOESS**	Discrete independent variable, therefore LOESS fitted curve just for visualization of gradient development.

Figure S4f		
**Statistical Analysis**	Linear Regression	
**Data**	Genotype: *hsflp;; act>>UAS-ubdGFP/UAS-eya*<br>Apical surface areas of control AFCs and eya^{OE} AFCs as a function of their distance to the anterior tip.<br>Data of each egg chamber were normalized for the distance and the apical surface area.	
**N**	control FCs: 71 FC, 3 EC	eya^{OE} FCs: 65 FC, 3 EC
**Linear regression**	Apical surface area ~ distance to anterior tip	
	F-statistic: 160.4 on 1 and 69 DF, $p < 0.001$, Adj. R^2=0.69	F-statistic: 0.4947 on 1 and 63 DF, $p = 0.48$, Adj. R^2=-0.0008

**Figure 5f**				
**Statistical Analysis**	Local polynomial regression fitting (LOESS)			
**Data**	Genotypes: *mirr>gfp & mirr>eya*<br>Egg chambers manually selected to sufficiently cover egg chamber development from stage 2 to 12. Therefore, frequencies are not representative of population frequencies.			
**N**	*mirr>gfp*: 153		*mirr>eya*: 157	
**LOESS**	*mirr>gfp*		*mirr>eya*	
**OCC proportion**	Degree of polynomial: 2<br>Level of CI: 0.95<br>Size of neighbourhood for fitting: 0.5		Degree of polynomial: 2<br>Level of CI: 0.95<br>Size of neighbourhood for fitting: 0.5	
**Statistical Analysis**	Multiple Linear Regression with Interaction<br>Y ~ germline size + genotype + germline size * genotype<br>If $p \geq 0.05$ for the interaction effect: multiple linear regression without interaction<br>Y ~ germline size + genotype			
**Data**	Genotypes: *mirr>gfp* & *mirr>eya*<br>Subset 1: 5000 µm^2 < germline size > 11650 µm^2<br>Subset 2: 11650 µm^2 < germline size > 31500 µm^2			
**N**	subset 1 *mirr>gfp*: 26	subset 1 *mirr>eya*: 31	subset 2 *mirr>gfp*: 41	Subset 2 *mirr>eya*: 44
**Multiple Linear Regression**	Y ~ germline size + genotype + germline size * genotype		Y ~ germline size + genotype	
**OCC proportion**	Subset1<br>F-statistic: 15.96 on 3 and 53 DF,<br>$p < 0.001$, Adj. R^2=0.445<br>Genotype*Germline Size p = 0.206		Subset1<br>F-statistic: 22.84 on 2 and 54 DF,<br>$p < 0.001$, Adj. R^2=0.438<br>Genotype effect: 0.025, p =0.028	
	Subset2<br>F-statistic: 53.71 on 3 and 81 DF,<br>$p < 0.001$, Adj. R^2=0.653<br>Genotype*Germline Size $p < 0.001$			

**Figure S5b**					
**Statistical Analysis**	Descriptive Statistics				
**Data**	Genotype: *mirr>gfp*<br>Cell count of posterior FCs without GFP in 2D-crosssections and its proportion of all FCs				
**N**	80 egg chambers				
**Descriptive Statistics**	Posterior GFP$^-$ cells				
	Min = 14	Max = 27	Mean = 21.23	SD = 2.47	CV = 0.116
	Proportion of posterior GFP$^-$ cells				
	Min = 0.206	Max = 0.4138	Mean = 0.3305	SD: 0.041	CV = 0.124
**Figure S5c**					
**Statistical Analysis**	Local polynomial regression fitting (LOESS)				
**Data**	Genotype: *mirr>gfp* and *mirr>eya*<br>Total cell count of egg chambers of all phases.				
**N**	*mirr>gfp*: 153		*mirr>eya*: 157		
**Local polynomial regression fitting parameters**	Degree of polynomial: 2<br>Level of CI: 0.95<br>Size of neighbourhood for fitting: 0.5		Degree of polynomial: 2<br>Level of CI: 0.95<br>Size of neighbourhood for fitting: 0.5		
**Statistical Analysis**	Descriptive statistics and two-tailed Student’s t-test				
**Data**	Total cell count of *mirr>gfp* and *mirr>eya* in all egg chambers with germline sizes > 6500 µm^2 and < 29000 µm^2 (Phase 2).				

**N**	mirr>gfp: 57		mirr>eya: 67	
**Descriptive Statistics**	Mean = 65<br>SD= 3.24		Mean = 60<br>SD = 3.93	
**Two-tailed unpaired t-test**	t = 7.97 with 122 DF, $p < 0.001$			
**Figure S5d**				
**Statistical Analysis**	Two intersection lines – fit the crossing point<br>Y = Ycross + (X-Xcross)*Slope			
**Data**	Genotype: mirr>gfp<br>Subset of germline sizes of > 6000 μm^2 and < 20000 μm^2<br>OCC proportion was used for linear regression of FC displacement. Proportion of posterior FC without GFP of each egg chamber with GFP signal was used to interpolate the proportion of posterior FCs without GFP.			
**N**	OCC proportion: 41 egg chambers		PFCs without GFP: 36 egg chambers	
**Fit of crossing point**	Ycross		Xcross	
**Best-fit values**	0.3161		11653	
**95% CI**	0.3032 – 0.3279		10632 - 12569	
**Goodness of Fit**	73 DF, $R^2 = 0.6377$			
**Figure S5e**				
**Statistical Analysis**	Two-Way Anova with Šídák's multiple comparisons test			
**Data**	Genotypes: *mirr>gfp* & *mirr>eya*<br>Only egg chambers with germline sizes < 40000 μm^2 (as mirr>eya egg chambers do not become bigger)<br>Subset 1: germline size < 11650 μm^2<br>Subset 2: germline size > 11650 μm^2			
**N**	*mirr>gfp* <11650μm^2<br>74	*mirr>eya* <11650μm^2<br>86	*mirr>gfp* >11650μm^2<br>74	*mirr>eya* >11650μm^2<br>71
**Two-Way Anova**				
**OCC proportion**	Interaction: 101.3 on 1 and 301 DF, $p < 0.001$<br>Germline size: 534.9 on 1 and 301 DF, $p < 0.001$<br>Genotype: 79.39 on 1 and 301 DF, $p < 0.001$			
**Multiple Comparison**	*mirr>gfp* <11650μm^2 vs. *mirr>eya* <11650μm^2		*mirr>gfp* >11650μm^2 vs. *mirr>eya* >11650μm^2	
**OCC proportion**	t = 0.8357 with 301 DF, p = 0.64		t = 13.11 with 301 DF, $p < 0.001$	

	Figure 6 c,h,i		
**Statistical Analysis**	Local polynomial regression fitting (LOESS), descriptive statistics and linear regression with y ~ germline size		
**Data**	Genotype: *wt* (w^{1118})<br>Multidimensional egg chamber morphology dataset. Parameters plotted against germline size.		
**N**	126 egg chambers manually selected to sufficiently cover egg chamber development from stage 2 to 12. Frequencies are not representative of population stage or size frequencies.<br>Phase 1: 62, Phase 2: 39, Phase 3: 26		
**Descriptive Statistics**	**c)** Interface Angle	**h)** OO-FC interface proportion	**i)** OO area proportion
**LOESS**	Degree of polynomial: 2<br>Level of CI: 0.95<br>Size of neighbourhood for fitting: 0.4	Degree of polynomial: 2<br>Level of CI: 0.95<br>Size of neighbourhood for fitting: 0.4	Degree of polynomial: 2<br>Level of CI: 0.95<br>Size of neighbourhood for fitting: 0.4
**Phase 1**	Min: 64.79<br>Max: 127.7<br>Mean: 99.27<br>SD: 15.34<br>CV: 0.15	Min: 0.079<br>Max: 0.184<br>Mean: 0.132<br>SD: 0.023<br>CV: 0.17	Min: 0.055<br>Max: 0.158<br>Mean: 0.086<br>SD: 0.019<br>CV: 0.22
**Phase 2**	Min: 31.44<br>Max: 156.1<br>Mean: 60.15<br>SD: 25.88<br>CV: 0.43	Min: 0.144<br>Max: 0.409<br>Mean: 0.292<br>SD: 0.073<br>CV: 0.25	Min: 0.067<br>Max: 0.376<br>Mean: 0.19<br>SD: 0.082<br>CV: 0.43
**Phase 3**	Min: 134.9<br>Max: 180<br>Mean: 166.6<br>SD: 14.49<br>CV: 0.087	Min: 0.378<br>Max: 0.746<br>Mean: 0.501<br>SD: 0.118<br>CV: 0.24	Min: 0.321<br>Max: 0.921<br>Mean: 0.56<br>SD: 0.204<br>CV: 0.36
**Linear Regression**	Phase 1 Slope: $-8.1*10^{-4}$<br>Phase 2 Slope: $2.32*10^{-3}$<br>Phase 3 Slope: $8.23*10^{-4}$	Phase 1 Slope: $4.4*10^{-6}$<br>Phase 2 Slope: $1.1*10^{-5}$<br>Phase 3 Slope: $8.0*10^{-6}$	Phase 1 Slope: $-3.17*10^{-6}$<br>Phase 2 Slope: $1.22*10^{-5}$<br>Phase 3 Slope: $1.41*10^{-5}$

Figure 6d			
**Statistical Analysis**	Descriptive statistics		
**Data**	Genotype: *wt* (w^{1118})<br>Interface Angle (°)		
**N**	116 EC, manually selected to cover stages 2 to 11<br>Phase 1: 62 egg chamber, phase 2: 39 egg chamber, phase 3: 15 egg chamber		
**Descriptive Statistics**	Phase 1	Phase 2	Phase 3
	Median: 100<br>Mean: 99<br>SE: 2<br>95% CI: 95 - 103	Median: 52<br>Mean: 60<br>SE: 4<br>95% CI: 52 – 69	Median: 161<br>Mean: 156<br>SE: 3<br>95% CI: 151 – 163

Figure 6e	
**Statistical Analysis**	Local polynomial regression fitting (LOESS)
**Data**	Genotype: *wt* (w^{1118})<br>Eya Levels (a.u.) of FCs at the nurse cell-oocyte boundary and the corresponding interface angle.
**N**	54 EC, manually selected to cover stages 2 to 10b.<br>Phase 1: 21 egg chamber, phase 2: 29 egg chamber, phase 3: 4 egg chamber
**LOESS**	Degree of polynomial: 2, Level of CI: 0.95, Size of neighbourhood for fitting: 0.7

Figure 6f			
**Statistical Analysis**	Linear Regression		
**Data**	Genotype: *wt (w*^{1118}*)*<br>Eya Levels of FCs at the nurse cell-oocyte boundary and the corresponding interface angle.		
**N**	36 EC, manually selected covering stage 2 to 9.		
**Linear Regression**	y= 44+0.64*x (y=interface angle, x=Eya levels)<br>R^2=0.54		
**Estimation of Eya Level giving rise to 90° Interface Angle**	x=71.57 a.u., 95% CI: 62.7 – 83.7 a.u.		
**Figure 6g**			
**Statistical Analysis**	Descriptive statistics		
**Data**	Genotype: *wt (w*^{1118}*)*<br>Eya Levels (a.u.) of FCs at the nurse cell-oocyte boundary.		
**N**	52 EC, manually selected to cover stages 3 to 10b.<br>Phase 1: 19 egg chamber, phase 2: 29 egg chamber, phase 3: 4 egg chamber		
**Descriptive Statistics**	Phase 1	Phase 2	Phase 3
	Median: 76<br>Mean: 77<br>SE: 4.<br>95% CI: 68-86	Median: 30<br>Mean: 42<br>SE: 5.7<br>95% CI: 31 – 54	Median: 146<br>Mean: 143<br>SE: 10<br>95% CI: 111 – 175
**Figure 6j**			
**Statistical Analysis**	Descriptive Statistics		
**Data**	Genotype: *w*^{1118}<br>Mean Eya fluorescence intensities of first 7 anterior rows of egg chambers from stage 5-10b.<br>Eya row intensities as a function of time. Time derived from reported stage durations [12,13]. Eya mean intensities of stages were assigned to the midpoint of each stage.<br>Assigned effective affinities are shown.		
**N**	stage 5: 5, stage 6: 5 , stage 7: 5 , stage 8: 4 , stage 9e: 8 , stage 9m: 9 , stage 10a: 5 , stage 10b: 5		
**Polynomial Regression**	For each row dynamic a least squares approximation by sixth-order polynomial constrained to have vanishing derivative at t = 0 and t = 36 hours was generated. See table S7 for coefficients.		
**Figure 6k**			
**Statistical Analysis**	Descriptive Statistics		
**Data**	Genotype: *w*^{1118}<br>Mean proportional distance from anterior tip of first 7 anterior rows of egg chambers from stage 5-10b.<br>Mean proportional distance as a function of time. Time derived from reported stage durations [12,13]. Mean proportional distance of stages were assigned to the midpoint of each stage.		
**N**	stage 5: 5, stage 6: 5 , stage 7: 5 , stage 8: 4 , stage 9e: 8 , stage 9m: 9 , stage 10a: 5 , stage 10b: 5		
**Polynomial Regression**	For each row dynamic a least squares approximation by fifth-order polynomial constrained to have vanishing derivative at t = 0 and t = 36 hours was generated. See table S8 for coefficients.		
**Figure 6m**			
**Statistical Analysis**	Descriptive Statistics		
**Data**	Simulations based on wt Eya levels<br>Measured interface angle of simulated images as a function of reported stage durations [12,13].		
**LOESS**	LOESS fitted curve to represent dynamics		

Figure 6n	
**Statistical Analysis**	Descriptive Statistics
**Data**	Simulations based on wt Eya levels<br>Measured OO-FC interface proportion of simulated images as a function of reported stage durations [12,13].
**LOESS**	LOESS fitted curve to represent dynamics
**Figure 6o**	
**Statistical Analysis**	Descriptive Statistics
**Data**	Simulations based on wt Eya levels<br>Measured proportional oocyte area of simulated images as a function of reported stage durations [12,13].
**LOESS**	LOESS fitted curve to represent dynamics

Figure 7b			
**Statistical Analysis**		Descriptive Statistics	
**Data**		Simulations with ectopic effective oocyte affinity from stage 5 onwards.<br>Measured interface angle of simulated images as a function of reported stage durations [12,13].	
**LOESS**		LOESS fitted curve to represent dynamics	
**Figure 7c**			
**Statistical Analysis**		Descriptive Statistics	
**Data**		Simulations with ectopic effective oocyte affinity from stage 5 onwards.<br>Measured OO-FC interface proportion of simulated images as a function of reported stage durations [12,13].	
**LOESS**		LOESS fitted curve to represent dynamics	
**Figure 7d**			
**Statistical Analysis**		Descriptive Statistics	
**Data**		Simulations with ectopic effective oocyte affinity from stage 5 onwards.<br>Measured proportional oocyte area of simulated images as a function of reported stage durations [12,13].	
**LOESS**		LOESS fitted curve to represent dynamics	
**Figure 7 g,k,l**			
**Statistical Analysis**		Local polynomial regression fitting (LOESS)	
**Data**		Genotypes: *gr1>gfp & gr1>eyaRNAi*<br>Egg chambers manually selected to sufficiently cover Phase 1 egg chamber development. Frequencies are not representative of population stage or size frequencies.	
**N**		*gr1>gfp*: 42	*gr1>eyaRNAi*: 60
**LOESS**		*gr1>gfp*	*gr1>eyaRNAi*
**g**	**Interface Angle**	Degree of polynomial: 2<br>Level of CI: 0.95<br>Size of neighbourhood for fitting: 0.7	Degree of polynomial: 2<br>Level of CI: 0.95<br>Size of neighbourhood for fitting: 0.7
**k**	**OO-FC interface**	Degree of polynomial: 2<br>Level of CI: 0.95<br>Size of neighbourhood for fitting: 0.7	Degree of polynomial: 2<br>Level of CI: 0.95<br>Size of neighbourhood for fitting: 0.7
**l**	**OO proportion**	Degree of polynomial: 2<br>Level of CI: 0.95<br>Size of neighbourhood for fitting: 0.7	Degree of polynomial: 2<br>Level of CI: 0.95<br>Size of neighbourhood for fitting: 0.7
**Statistical Analysis**		Multiple Linear Regression with Interaction<br>Y ~ germline size + genotype + germline size * genotype<br>If p ≥ 0.05 for the interaction effect: multiple linear regression without interaction<br>Y ~ germline size + genotype	

**Data**		Genotypes: *gr1>gfp* & *gr1>eyaRNAi*<br>Subset 1: 500 μm^2 < germline size < 1609 μm^2<br>Subset 2: 1609 μm^2 < germline size < 6500 μm^2			
**N**		subset 1 *gr1>gfp*: 13	subset 1 *gr1>eyaRNAi*: 17	subset 2 *gr1>gfp*: 29	subset 2 *gr1>eyaRNAi*: 43
**Multiple Linear Regression**		Y ~ germline size + genotype + germline size * genotype		Y ~ germline size + genotype	
**g**	**Interface Angle**	Subset 1<br>F-statistic: 3.514 on 3 and 26 DF, p = 0.029, Adj. R^2=0.0.206<br>Genotype*Germline Size p = 0.361		Subset 1<br>F-statistic: 4.863 on 2 and 27 DF, p = 0.0157, Adj. R^2=0.2104<br>Genotype effect: 0.019, p = 0.029	
		Subset 2<br>F-statistic: 19.48 on 3 and 68 DF, p < 0.001, Adj. R^2=0.4385<br>Genotype*Germline Size p = 0.222		Subset 2<br>F-statistic: 28.25 on 2 and 69 DF, p < 0.001, Adj. R^2=0.4343<br>Genotype effect: 0.019, p < 0.001	
**k**	**OO-FC interface proportion**	Subset 1<br>F-statistic: 8.874 on 3 and 26 DF, p < 0.001, Adj. R^2=0.4489<br>Genotype*Germline Size p = 0.866		Subset 1<br>F-statistic: 13.79 on 2 and 27 DF, p < 0.001, Adj. R^2=0.4687<br>Genotype effect: 0.03, p < 0.001	
		Subset 2<br>F-statistic: 63.09 on 3 and 68 DF, p < 0.001, Adj. R^2=0.724<br>Genotype*Germline Size p = 0.0364			
**l**	**OO area proportion**	Subset 1<br>F-statistic: 16.2 on 3 and 26 DF, p < 0.001, Adj. R^2=0.6113<br>Genotype*Germline Size p = 0.214		Subset 1<br>F-statistic: 22.96 on 2 and 27 DF, p < 0.001, Adj. R^2=0.6023<br>Genotype effect: 0.019, p <0.001	
		Subset 2<br>F-statistic: 37.17 on 3 and 68 DF, p < 0.001, Adj. R^2=0.4923<br>Genotype*Germline Size p = 0.00189			

Figure 7i

**Statistical Analysis**	Two-tailed unpaired Student's t-test	
**Data**	Genotypes: *gr1>gfp* & *gr1>eyaRNAi*<br>Coefficient of variation of NC-FC interface proportions of NCs within their respective egg chamber<br>Only egg chambers with germline sizes > 2000 μm^2 and <5500 μm^2 were selected.<br>At least 3 NCs covering all rows were used to determine the CV of individual egg chambers.	
**N**	*gr1>gfp* : 15 egg chambers, 71 NCs	*gr1>eyaRNAi:* 15 egg chambers, 89 NCs
**Descriptive statistics**	*gr1>gfp* : mean CV = 0.1191, SE = 0.01382	*gr1>eyaRNAi:* mean CV = 0.2484, SE = 0.01957
**Two-tailed unpaired t-test**	Two-tailed, t=5.397 with 28DF, p < 0.001	

Figure 7j

**Statistical Analysis**	Two-tailed unpaired Welch's t-test	
**Data**	Genotypes: *gr1>gfp* & *gr1>eyaRNAi*<br>Coefficient of variation of NC areas normalized to germline area<br>Only egg chambers of with germline sizes >2000 μm^2 and <5500 μm^2 were selected.<br>At least 3 NCs covering all rows were used to determine the CV of individual egg chambers.	
**N**	*gr1>gfp* : 15 egg chambers, 71 NCs	*gr1>eyaRNAi:* 15 egg chambers, 89 NCs
**Descriptive statistics**	*gr1>gfp* : mean = 0.2008, SE = 0.01422	*gr1>eyaRNAi:* mean = 0.3619, SE = 0.02489
**Welch's t-test**	Two-tailed, t=5.618 with 22.26 DF, p < 0.001	

<table>
<tr><th colspan="5">Figure S7b</th></tr>
<tr><td>Statistical Analysis</td><td colspan="4">Descriptive Statistics</td></tr>
<tr><td>Data</td><td colspan="4">Genotype: gr1 > eyaRNAi
Germline sizes of egg chambers grouped by Eya presence into Eya^+, sporadic Eya and no Eya.</td></tr>
<tr><td>N</td><td>Eya present: 6</td><td colspan="2">sporadic Eya: 15</td><td>no Eya: 75</td></tr>
<tr><td>Descriptive Statistics</td><td>Mean: 743 μm^2
Min: 515.8 μm^2
Max: 995.1 μm^2
95% CI: 554.3 – 933.3 μm^2</td><td colspan="2">Mean: 1609 μm^2
Min: 1088 μm^2
Max: 2787 μm^2
95% CI: 1300 – 1918 μm^2</td><td>Mean: 8622 μm^2
Min: 1181 μm^2
Max: 26206 μm^2
95% CI: 7167 – 10077 μm^2</td></tr>
<tr><th colspan="5">Figure S7c,d,e</th></tr>
<tr><td>Statistical Analysis</td><td colspan="4">Two-Way Anova with Šídák's multiple comparisons test</td></tr>
<tr><td>Data</td><td colspan="4">Genotypes: gr1>gfp & gr1>eyaRNAi
Subset 1: germline size < 1600 μm^2
Subset 2: germline size > 1600 & <6500 μm^2</td></tr>
<tr><td rowspan="2">N</td><td colspan="2">gr1>gfp</td><td colspan="2">gr1>eyaRNAi</td></tr>
<tr><td><1600 μm^2:
13</td><td>>1600 & <6500 μm^2:
27</td><td><1600 μm^2:
16</td><td><1600 & <6500 μm^2:
36</td></tr>
<tr><td>Two-Way Anova</td><td colspan="4"></td></tr>
<tr><td>Interface Angle</td><td colspan="4">Interaction: 6.978 on 1 and 88 DF, p = 0.01
Germline size: 14.26 on 1 and 88 DF, p < 0.001
Genotype: 438.32 on 1 and 88 DF, p < 0.001</td></tr>
<tr><td>OO-FC interface</td><td colspan="4">Interaction: 20.49 on 1 and 88 DF, p < 0.001
Germline size: 21.76on 1 and 88 DF, p < 0.001
Genotype: 68.3 on 1 and 88 DF, p < 0.001</td></tr>
<tr><td>OO proportion</td><td colspan="4">Interaction: 13.28 on 1 and 88 DF, p < 0.001
Germline size: 0.444 on 1 and 88 DF, p = 0.51
Genotype: 41.26 on 1 and 88 DF, p < 0.001</td></tr>
<tr><td>Multiple Comparison</td><td colspan="2">gr1>gfp vs. gr1>eyaRNAi (<1600 μm^2)</td><td colspan="2">gr1>gfp vs. gr1>eyaRNAi (>1600 & <6500 μm^2)</td></tr>
<tr><td>Interface Angle</td><td colspan="2">t = 2.147 with 88 DF, p = 0.07</td><td colspan="2">t = 87.839 with 88 DF, p < 0.001</td></tr>
<tr><td>OO-FC interface</td><td colspan="2">t = 2.262 with 88 DF, p = 0.05</td><td colspan="2">t = 11.35 with 88 DF, p < 0.001</td></tr>
<tr><td>OO proportion</td><td colspan="2">t = 1.682 with 88 DF, p = 0.18</td><td colspan="2">t = 8.936 with 88 DF, p < 0.001</td></tr>
</table>

<table>
<tr><th colspan="6">Figure 8b</th></tr>
<tr><td colspan="2">Statistical Analysis</td><td colspan="4">Descriptive Statistics</td></tr>
<tr><td colspan="2">Data</td><td colspan="4">Simulations with ectopic effective nurse cell affinity from stage 7 onwards.
Measured interface angle of simulated images as a function of reported stage durations [12,13].</td></tr>
<tr><td colspan="2">LOESS</td><td colspan="4">LOESS fitted curve to represent dynamics</td></tr>
<tr><th colspan="6">Figure 8c</th></tr>
<tr><td colspan="2">Statistical Analysis</td><td colspan="4">Descriptive Statistics</td></tr>
<tr><td colspan="2">Data</td><td colspan="4">Simulations with ectopic effective nurse cell affinity from stage 7 onwards.
Measured OO-FC interface proportion of simulated images as a function of reported stage durations [12,13].</td></tr>
<tr><td colspan="2">LOESS</td><td colspan="4">LOESS fitted curve to represent dynamics</td></tr>
<tr><th colspan="6">Figure 8d</th></tr>
<tr><td colspan="2">Statistical Analysis</td><td colspan="4">Descriptive Statistics</td></tr>
<tr><td colspan="2">Data</td><td colspan="4">Simulations with ectopic effective nurse cell affinity from stage 7 onwards.
Measured proportional oocyte area of simulated images as a function of reported stage durations [12,13].</td></tr>
<tr><td colspan="2">LOESS</td><td colspan="4">LOESS fitted curve to represent dynamics</td></tr>
<tr><th colspan="6">Figure 8g,h,j</th></tr>
<tr><td colspan="2">Statistical Analysis</td><td colspan="4">Local polynomial regression fitting (LOESS)</td></tr>
<tr><td colspan="2">Data</td><td colspan="4">Genotypes: mirr>gfp & mirr>eya
Egg chambers manually selected to sufficiently cover egg chamber development from stage 2 to 12. Therefore, frequencies are not representative of population frequencies.</td></tr>
<tr><td colspan="2">N</td><td colspan="2">mirr>gfp: 153</td><td colspan="2">mirr>eya: 157</td></tr>
<tr><td colspan="2">LOESS</td><td colspan="2">mirr>gfp</td><td colspan="2">mirr>eya</td></tr>
<tr><td>g</td><td>Interface Angle</td><td colspan="2">Degree of polynomial: 2
Level of CI: 0.95
Size of neighbourhood for fitting: 0.4</td><td colspan="2">Degree of polynomial: 2
Level of CI: 0.95
Size of neighbourhood for fitting: 0.4</td></tr>
<tr><td>h</td><td>OO-FC interface</td><td colspan="2">Degree of polynomial: 2
Level of CI: 0.95
Size of neighbourhood for fitting: 0.3</td><td colspan="2">Degree of polynomial: 2
Level of CI: 0.95
Size of neighbourhood for fitting: 0.8</td></tr>
<tr><td>j</td><td>OO proportion</td><td colspan="2">Degree of polynomial: 2
Level of CI: 0.95
Size of neighbourhood for fitting: 0.4</td><td colspan="2">Degree of polynomial: 2
Level of CI: 0.95
Size of neighbourhood for fitting: 0.4</td></tr>
<tr><td colspan="2">Statistical Analysis</td><td colspan="4">Multiple Linear Regression with Interaction
Y ~ germline size + genotype + germline size * genotype
If p ≥ 0.05 for the interaction effect: multiple linear regression without interaction
Y ~ germline size + genotype</td></tr>
<tr><td colspan="2">Data</td><td colspan="4">Genotypes: mirr>gfp & mirr>eya
Subset 1: 5000 μm^2 < germline size > 11650 μm^2
Subset 2: 11650 μm^2 < germline size > 31500 μm^2</td></tr>
<tr><td colspan="2">N</td><td>subset 1 mirr>gfp: 26</td><td>subset 1 mirr>eya: 31</td><td>subset 2 mirr>gfp: 41</td><td>Subset 2 mirr>eya: 44</td></tr>
<tr><td colspan="2">Multiple Linear Regression</td><td colspan="2">Y ~ germline size + genotype + germline size * genotype</td><td colspan="2">Y ~ germline size + genotype</td></tr>
<tr><td>g</td><td>Interface Angle</td><td colspan="2">Subset1
F-statistic: 19.1 on 3 and 53 DF,
$p < 0.001$, Adj. R^2=0.4923
Genotype*Germline Size $p < 0.001$</td><td colspan="2"></td></tr>
</table>

		Subset2<br>F-statistic: 85.22 on 3 and 81 DF,<br>$p < 0.001$, Adj. R^2=0.75<br>Genotype*Germline Size p = 0.826	Subset2<br>F-statistic: 129.3 on 2 and 82 DF, $p < 0.001$,<br>Adj. R^2=0.75<br>Genotype effect: $p < 0.001$
**h**	**OO-FC interface proportion**	Subset 1<br>F-statistic: 8.879 on 3 and 53 DF,<br>$p < 0.001$, Adj. R^2= 0.297<br>Genotype*Germline Size p = 0.054	Subset1<br>F-statistic: 12.93 on 2 and 54 DF,<br>$p < 0.001$, Adj. R^2=0.2988<br>Genotype effect: p =0.1434
		Subset 2<br>F-statistic: 81.86 on 3 and 81 DF,<br>$p < 0.001$, Adj. R^2=0.74<br>Genotype*Germline Size $p < 0.001$	
**j**	**OO area proportion**	Subset 1<br>F-statistic: 34.36 on 3 and 53 DF,<br>$p < 0.001$, Adj. R^2=0.64<br>Genotype*Germline Size $p < 0.001$	
		Subset 2<br>F-statistic: 40.57 on 3 and 81 DF,<br>$p < 0.001$, Adj. R^2=0.59<br>Genotype*Germline Size $p < 0.001$	

Figure 8i

**Statistical Analysis**	Two-Way Anova with Šídák's multiple comparisons test	
**Data**	Genotypes: *mirr>gfp* & *mirr>eya*<br>NC-FC interface proportion of NC perimeter for individual nurse cells .NC are grouped by NC row and averaged within each egg chamber. NC row averages of different egg chambers are analysed.	
**N**	*mirr>gfp*<br>egg chamber: N=8<br>row averages:<br>A: 8, MA: 6, MP: 8, P: 8	*mirr>eya*<br>egg chambers: N=9<br>individual nurse cells:<br>A: 7, MA: 9, MP: 9, P: 9
**Descriptive Statistics**	*mirr>gfp*<br>A: mean = 0.488, SE = 0.032<br>MA: mean = 0.398, SE = 0.021<br>MP: mean = 0.306, SE = 0.01<br>P: mean = 0.287, SE = 0.014	*mirr>eya*<br>A: mean = 0.416, SE = 0.048<br>MA: mean = 0.337, SE = 0.021<br>MP: mean = 0.297, SE = 0.018<br>P: mean = 0.406, SE = 0.014
**F**	Interaction: 6.828 on 3 and 56DF, $p < 0.001$<br>NC row: 13.86 on 3 and 56 DF, $p < 0.001$<br>Genotype: 0.09731 on 3 and 56 DF, p = 0.76	
**Multiple Comparison**	*mirr>gfp* vs *mirr>eya* for each NC row<br>A: t= 2.062 with 56 DF, p = 0.16<br>MA: t= 1.701 with 56 DF, p = 0.33<br>MP: t= 0.2504 with 56 DF, $p > 0.99$<br>P: t= 3.644 with 56 DF, p = 0.002	

Figure S8a,b,c

**Statistical Analysis**	Two-Way Anova with Šídák's multiple comparisons test			
**Data**	Genotypes: *mirr>gfp* & *mirr>eya*<br>Only egg chambers with germline sizes < 40000 µm^2 (as mirr>eya egg chambers do not become bigger)<br>Subset 1: germline size < 11650 µm^2<br>Subset 2: germline size > 11650 µm^2			
**N**	*mirr>gfp* <11650µm^2<br>74	*mirr>eya*<br><11650µm^2<br>86	*mirr>gfp*<br>>11650µm^2<br>74	*mirr>eya*<br>>11650µm^2<br>71
**Two-Way Anova**				

<table>
<tr><td>Interface Angle</td><td colspan="12">Interaction: 3.653 on 1 and 301 DF, p = 0.06
Germline size: 30.21 on 1 and 301 DF, p < 0.001
Genotype: 47.55 on 1 and 301 DF, p < 0.001</td></tr>
<tr><td>OO-FC interface</td><td colspan="12">Interaction: 112.7 on 1 and 301 DF, p < 0.001
Germline size: 311.9 on 1 and 301 DF, p < 0.001
Genotype: 98.45 on 1 and 301 DF, p < 0.001</td></tr>
<tr><td>OO proportion</td><td colspan="12">Interaction: 27.4 on 1 and 301 DF, p < 0.001
Germline size: 189.8 on 1 and 301 DF, p < 0.001
Genotype: 10.85 on 1 and 301 DF, p = 0.001</td></tr>
<tr><td>Multiple Comparison</td><td colspan="6"><i>mirr>gfp</i> <11650µm^2 vs. <i>mirr>eya</i> <11650µm^2</td><td colspan="6"><i>mirr>gfp</i> >11650µm^2 vs. <i>mirr>eya</i> >11650µm^2</td></tr>
<tr><td>OO-FC interface</td><td colspan="6">t = 0.5035 with 301 DF, p = 0.85</td><td colspan="6">t = 14.2 with 301 DF, p < 0.001</td></tr>
<tr><td>OO proportion</td><td colspan="6">t = 1.412 with 301 DF, p = 0.29</td><td colspan="6">t = 5.901 with 301 DF, p < 0.001</td></tr>
<tr><td>Interface Angle</td><td colspan="6">t = 3.609 with 301 DF, p < 0.001</td><td colspan="6">t = 6.087 with 301 DF, p < 0.001</td></tr>
<tr><td colspan="13">Figure S8d,e,f</td></tr>
<tr><td>Statistical Analysis</td><td colspan="12">Two-Way Anova with Šídák's multiple comparisons test</td></tr>
<tr><td>Data</td><td colspan="12">Genotypes: <i>tj>gfp</i> & <i>tj>λtop</i>
Subset 1: germline size >600µm^2 & <i><6500</i>µm^2
Subset 2: germline size <i>>6500</i>µm^2 <i>&</i> <31500µm^2
Subset 3: germline size <i>>31500</i>µm^2 <i>&</i> <62000µm^2</td></tr>
<tr><td>N</td><td colspan="3">tj>gfp subset 1
N: 36</td><td colspan="3">tj>λtop subset 1
N: 55</td><td colspan="3">tj>gfp subset 2
N: 21</td><td colspan="3">tj>λtop subset 2
N: 13</td></tr>
<tr><td>Two-Way Anova</td><td colspan="12"></td></tr>
<tr><td>Interface Angle</td><td colspan="12">Interaction: 35.85 on 2 and 214 DF, p < 0.001
Germline size: 225.2 on 2 and 214 DF, p < 0.001
Genotype: 62.85 on 1 and 214 DF, p < 0.001</td></tr>
<tr><td>OO-FC interface</td><td colspan="12">Interaction: 3.34 on 2 and 214 DF, p = 0.04
Germline size: 296.2 on 2 and 214 DF, p < 0.001
Genotype: 5.136 on 1 and 214 DF, p = 0.02</td></tr>
<tr><td>OO proportion</td><td colspan="12">Interaction: 7.894 on 2 and 214 DF, p < 0.001
Germline size: 262.1 on 2 and 214 DF, p < 0.001
Genotype: 11.92 on 1 and 214 DF, p < 0.001</td></tr>
<tr><td>Multiple Comparison</td><td colspan="4">Subset 1: tj>gfp vs. tj>λtop</td><td colspan="4">Subset 2: tj>gfp vs. tj>λtop</td><td colspan="4">Subset 3: tj>gfp vs. tj>λtop</td></tr>
<tr><td>Interface Angle</td><td colspan="4">t = 1.125 with 214 DF,
p = 0.6</td><td colspan="4">t = 0.005 with 214 DF,
p > 0.99</td><td colspan="4">t = 9.29 with 214 DF,
p < 0.001</td></tr>
<tr><td>OO-FC interface</td><td colspan="4">t = 0.103 with 214 DF,
p > 0.99</td><td colspan="4">t = 0.0165 with 214 DF,
p > 0.99</td><td colspan="4">t = 2.77 with 214 DF,
p = 0.02</td></tr>
<tr><td>OO proportion</td><td colspan="4">t = 0.107 with 214 DF,
p > 0.99</td><td colspan="4">t = 0.027 with 214 DF,
p > 0.99</td><td colspan="4">t = 4.26 with 214 DF,
p < 0.001</td></tr>
</table>

**Figure 9b**					
**Statistical Analysis**		Descriptive Statistics			
**Data**		Simulations with ectopic effective oocyte affinity from stage 6 onwards.<br>Measured interface angle of simulated images as a function of reported stage durations [12,13].			
**LOESS**		LOESS fitted curve to represent dynamics			
**Figure 9c**					
**Statistical Analysis**		Descriptive Statistics			
**Data**		Simulations with ectopic effective oocyte affinity from stage 6 onwards.<br>Measured OO-FC interface proportion of simulated images as a function of reported stage durations [12,13].			
**LOESS**		LOESS fitted curve to represent dynamics			
**Figure 9d**					
**Statistical Analysis**		Descriptive Statistics			
**Data**		Simulations with ectopic effective oocyte affinity from stage 6 onwards.<br>Measured proportional oocyte area of simulated images as a function of reported stage durations [12,13].			
**LOESS**		LOESS fitted curve to represent dynamics			
**Figure 9g,h,i**					
**Statistical Analysis**		Local polynomial regression fitting (LOESS)			
**Data**		Genotypes: *tj>gfp & tj>λtop*<br>Egg chambers manually selected to sufficiently cover egg chamber development from stage 2 to 12. Therefore, frequencies are not representative of population frequencies.			
**N**		*tj>gfp*: 122		*tj>λtop*: 109	
**LOESS**		*tj>gfp*		*tj>λtop*	
**g**	**Interface Angle**	Degree of polynomial: 2<br>Level of CI: 0.95<br>Size of neighbourhood for fitting: 0.3		Degree of polynomial: 2<br>Level of CI: 0.95<br>Size of neighbourhood for fitting: 0.3	
**h**	**OO-FC interface proportion**	Degree of polynomial: 2<br>Level of CI: 0.95<br>Size of neighbourhood for fitting: 0.3		Degree of polynomial: 2<br>Level of CI: 0.95<br>Size of neighbourhood for fitting: 0.3	
**i**	**OO proportion**	Degree of polynomial: 2<br>Level of CI: 0.95<br>Size of neighbourhood for fitting: 0.3		Degree of polynomial: 2<br>Level of CI: 0.95<br>Size of neighbourhood for fitting: 0.3	
**Statistical Analysis**		Multiple Linear Regression with Interaction<br>Y ~ germline size + genotype + germline size * genotype<br>If p ≥ 0.05 for the interaction effect: multiple linear regression without interaction<br>Y ~ germline size + genotype			
**Data**		Genotypes: *tj>gfp & tj>λtop*<br>Subset 1: 6500 μm^2 < germline size > 31500μm^2<br>Subset 2: 31500 μm^2 < germline size > 62000 μm^2			
**N**		subset 1 *tj>gfp*: 39	subset 1 *tj>λtop*: 58	subset 2 *tj>gfp*: 18	subset 2 *tj>λtop*: 10
**Multiple Linear Regression**		Y ~ germline size + genotype + germline size * genotype		Y ~ germline size + genotype	
**g**	**Interface Angle**	Subset1<br>F-statistic: 0.2759 on 3 and 93 DF,<br>p = 0.84, Adj. R^2=-0.023			
		Subset2			

		F-statistic: 36.23 on 3 and 24 DF,<br>$p < 0.001$, Adj. R^2=0.7965<br>Genotype*Germline Size p = 0.017	
**h**	**OO-FC interface proportion**	Subset 1<br>F-statistic: 450.5 on 3 and 93 DF,<br>$p < 0.001$, Adj. R^2= 0.9335<br>Genotype*Germline Size p = 0.0222	
		Subset 2<br>F-statistic: 18.82 on 3 and 30 DF,<br>$p < 0.001$, Adj. R^2=0.6645<br>Genotype*Germline Size p = 0.9727	Subset 2<br>F-statistic: 29.41 on 2 and 25 DF,<br>$p < 0.001$, Adj. R^2=0.678<br>Genotype effect: $p < 0.001$
**i**	**OO area proportion**	Subset 1<br>F-statistic: 252.8 on 3 and 93 DF,<br>$p < 0.001$, Adj. R^2=0.887<br>Genotype*Germline Size $p < 0.001$	
		Subset 2<br>F-statistic: 27.16 on 3 and 24 DF,<br>$p < 0.001$, Adj. R^2= 0.744<br>Genotype*Germline Size p = 0.478	Subset 2<br>F-statistic: 41.27 on 2 and 25 DF,<br>$p < 0.001$, Adj. R^2=0.749<br>Genotype effect: $p < 0.001$

Supplementary References

1. Allen, S. M. & Cahn, J. W. Microscopic Theory for Antiphase Boundary Motion and Its Application to Antiphase Domain Coarsening. *Acta Metallurgica* 27, 1085–1095 (1979).
2. Nonomura, M. Study on Multicellular Systems Using a Phase Field Model. *PLoS ONE* 7, e33501 (2012).
3. Moure, A. & Gomez, H. Phase-Field Modeling of Individual and Collective Cell Migration. *Archives of Computational Methods in Engineering* 28, 311–344 (2021).
4. Wenzel, D. & Voigt, A. Multiphase field models for collective cell migration. *Phys. Rev. E* 104, 054410 (5 Nov. 2021).
5. Garcke, H., Lam, K. F. & Rocca, E. Optimal control of treatment time in a diffuse interface model of tumor growth. *Appl. Math. Optim.* 78, 495–544 (2018).
6. Fritz, M., Lima, E. A. B. F., Nikolić, V., Oden, J. T. & Wohlmuth, B. Local and nonlocal phase-field models of tumor growth and invasion due to ECM degradation. *Math. Models Methods Appl. Sci.* 29, 2433–2468 (2019).
7. Chen, L.-Q. & Yang, W. Computer simulation of the domain dynamics of a quenched system with a large number of nonconserved order parameters: The grain-growth kinetics. *Physical Review B* 50, 15752–15756 (1994).
8. Fan, D., Geng, C. & Chen, L.-Q. Computer simulation of topological evolution in 2-D grain growth using a continuum diffuse-interface field model. *Acta Materialia* 45, 1115–1126 (1997).
9. Modica, L. & Mortola, S. Un esempio di Γ−-convergenza. *Boll. Un. Mat. Ital. B (5)* 14, 285–299 (1977).
10. Ahrens, J., Geveci, B. & Law, C. Paraview: An end-user tool for large data visualization. *The visualization handbook* 717 (2005).
11. Ayachit, U. *The ParaView Guide: A Parallel Visualization Application* (Kitware, Inc., Clifton Park, NY, USA, 2015).
12. Spradling, A. C. Developmental genetics of oogenesis. *The development of Drosophila melanogaster*, 1-70 (1993).
13. Horne-Badovinac, S. & Bilder, D. Mass transit: epithelial morphogenesis in the Drosophila egg chamber. *Developmental dynamics : an official publication of the American Association of Anatomists* 232, 559-574, doi:10.1002/dvdy.20286 (2005).
