# Supplementary material for: Eya-controlled affinity between cell lineages drives tissue self-organization during Drosophila oogenesis
Source: Nat Commun. 2022 Oct 26;13:6377. doi: 10.1038/s41467-022-33845-1 (PMC9605976; doi:10.1038/s41467-022-33845-1)
Supplement: Supplementary file 2 — Description of Additional Supplementary Files [file 41467_2022_33845_MOESM2_ESM.pdf]

Description of Additional Supplementary Files

File Name: Supplementary Movie 1
Description: Phase field model simulating the collective behaviour of 3 FCs in contact with nurse cells. The central cell is developing a relatively higher affinity.

File Name: Supplementary Movie 2
Description: Phase field model simulating the collective behaviour of 3 FCs as a function of their affinity to the germline. The gradient in affinity is based on Eya levels quantified in wild type egg chambers.

File Name: Supplementary Movie 3
Description: Phase field model simulating the collective behaviour of FCs as a function of their affinity to the germline representing the wild type condition. The gradient in affinity is based on Eya levels quantified in wild type egg chambers.

File Name: Supplementary Movie 4
Description: Phase field model simulating the collective behaviour of FCs as a function of their affinity to the germline. No affinity gradient in AFCs. High-affinity dynamic of cell 1 was assigned to all 6 AFCs.

File Name: Supplementary Movie 5
Description: Phase field model simulating the collective behaviour of FCs as a function of their affinity to the germline. AFC row 3 has reduced affinity.

File Name: Supplementary Movie 6
Description: Phase field model simulating the collective behaviour of FCs as a function of their affinity to the germline. Ectopic affinity assigned to rows 6-8.

File Name: Supplementary Movie 7
Description: Phase field model simulating the collective behaviour of the oocyte and the nurse cell compartment as a function of their effective affinities for the follicle epithelium, simulated by changing the boundary affinity. Boundary affinities were based on Eya levels and the AP-positions of rows 1-7 in wild type egg chambers.

File Name: Supplementary Movie 8
Description: Phase field model simulating the collective behaviour of the oocyte and the nurse cell compartment as a function of their effective affinities for the follicle epithelium, simulated by changing the boundary affinity. Boundary affinities induce premature effective oocyte affinity during phase 1.

File Name: Supplementary Movie 9
Description: Phase field model simulating the collective behaviour of the oocyte and the nurse cell compartment as a function of their effective affinities for the follicle epithelium, simulated by changing the boundary affinity. Boundary affinities induce effective nurse cell affinity along 80% of the boundary (representing AFCs + MBFCs) after phase 1.

File Name: Supplementary Movie 10
Description: Phase field model simulating the collective behaviour of the oocyte and the nurse cell compartment as a function of their effective affinities for the follicle epithelium, simulated by changing the boundary affinity. Boundary affinities induce effective oocyte affinity along 100% of the boundary after phase 1.
