# Supplementary material for: Eya-controlled affinity between cell lineages drives tissue self-organization during Drosophila oogenesis
Source: Nat Commun. 2022 Oct 26;13:6377. doi: 10.1038/s41467-022-33845-1 (PMC9605976; doi:10.1038/s41467-022-33845-1)
Supplement: Supplementary file 13 — Reporting Summary [file 41467_2022_33845_MOESM13_ESM.pdf]

Corresponding author(s): Anne-Kathrin Classen
Last updated by author(s): Sep 27, 2022

Reporting Summary

Nature Portfolio wishes to improve the reproducibility of the work that we publish. This form provides structure for consistency and transparency in reporting. For further information on Nature Portfolio policies, see our Editorial Policies and the Editorial Policy Checklist.

Statistics

For all statistical analyses, confirm that the following items are present in the figure legend, table legend, main text, or Methods section.

n/a	Confirmed	
☐	☒	The exact sample size (*n*) for each experimental group/condition, given as a discrete number and unit of measurement
☐	☒	A statement on whether measurements were taken from distinct samples or whether the same sample was measured repeatedly
☐	☒	The statistical test(s) used AND whether they are one- or two-sided<br>*Only common tests should be described solely by name; describe more complex techniques in the Methods section.*
☒	☐	A description of all covariates tested
☐	☒	A description of any assumptions or corrections, such as tests of normality and adjustment for multiple comparisons
☐	☒	A full description of the statistical parameters including central tendency (e.g. means) or other basic estimates (e.g. regression coefficient) AND variation (e.g. standard deviation) or associated estimates of uncertainty (e.g. confidence intervals)
☐	☒	For null hypothesis testing, the test statistic (e.g. *F*, *t*, *r*) with confidence intervals, effect sizes, degrees of freedom and *P* value noted<br>*Give P values as exact values whenever suitable.*
☒	☐	For Bayesian analysis, information on the choice of priors and Markov chain Monte Carlo settings
☒	☐	For hierarchical and complex designs, identification of the appropriate level for tests and full reporting of outcomes
☒	☐	Estimates of effect sizes (e.g. Cohen's *d*, Pearson's *r*), indicating how they were calculated

Our web collection on statistics for biologists contains articles on many of the points above.

Software and code

Policy information about availability of computer code

Data collection	LEICA LAS X Software, FIJI, MATLAB R2022a
Data analysis	FIJI, MATLAB R2022a, R (R version 4.0.5), GraphPad (GraphPad Prism 9), Paraview 5.10.1

For manuscripts utilizing custom algorithms or software that are central to the research but not yet described in published literature, software must be made available to editors and reviewers. We strongly encourage code deposition in a community repository (e.g. GitHub). See the Nature Portfolio guidelines for submitting code & software for further information.

Data

Policy information about availability of data

All manuscripts must include a data availability statement. This statement should provide the following information, where applicable:
- Accession codes, unique identifiers, or web links for publicly available datasets
- A description of any restrictions on data availability
- For clinical datasets or third party data, please ensure that the statement adheres to our policy

The data generated in this study are provided in the Source Data file.

Human research participants

Policy information about studies involving human research participants and Sex and Gender in Research.

Reporting on sex and gender	not applicable
Population characteristics	not applicable
Recruitment	not applicable
Ethics oversight	not applicable

Note that full information on the approval of the study protocol must also be provided in the manuscript.

Field-specific reporting

Please select the one below that is the best fit for your research. If you are not sure, read the appropriate sections before making your selection.

☒ Life sciences ☐ Behavioural & social sciences ☐ Ecological, evolutionary & environmental sciences

For a reference copy of the document with all sections, see nature.com/documents/nr-reporting-summary-flat.pdf

Life sciences study design

All studies must disclose on these points even when the disclosure is negative.

Sample size	Sample size was chosen to cover developmental processes sufficiently and to provide a reliable sample size for statistical analysis. Sample sizes vary dependent on the experiment and statistical analysis. For details see the file "Reproducibility and Statistics".
Data exclusions	Data were only excluded if proper sample preparation for microscopy failed.
Replication	To confirm reproducibility of our data, we performed each experiment at least 5 times independently and found no problem with reproducibility.
Randomization	Due to genetic experiments rather than experiments based on treatments, randomization is not applicable.
Blinding	Blinding was not necessary as the quantification of data was based on defined and extensive objective morphological measurements.

Reporting for specific materials, systems and methods

We require information from authors about some types of materials, experimental systems and methods used in many studies. Here, indicate whether each material, system or method listed is relevant to your study. If you are not sure if a list item applies to your research, read the appropriate section before selecting a response.

Materials & experimental systems

n/a	Involved in the study	
☐	☒	Antibodies
☒	☐	Eukaryotic cell lines
☒	☐	Palaeontology and archaeology
☐	☒	Animals and other organisms
☒	☐	Clinical data
☒	☐	Dual use research of concern

Methods

n/a	Involved in the study	
☒	☐	ChIP-seq
☒	☐	Flow cytometry
☒	☐	MRI-based neuroimaging

Antibodies

Antibodies used	mouse anti-β-catenin (1:100, DSHB, N27A1), rat anti-E-cadherin (1:50, DSHB, DCAD2), rabbit anti-GFP (1:200, Thermo Fisher, G10362), rat anti-RFP (1:20, gift from H. Leonhardt, Chromotek 5F8), mouse anti-β-gal (1:1000, Promega Z378B), mouse anti-Eya (1:100, DSHB eya10H6), rabbit anti-pMad (1:200, abcam ab52903), DAPI (0.25 ng/µl, Sigma), Phalloidin (Alexa Fluor 488, Alexa Fluor 647 and Alexa Fluor 555, Molecular Probes, or Phalloidin-TRITC, Sigma), goat anti-mouse Alexa Fluor488 (Abcam, AB150117, 1:500), goat anti-rat Alexa Fluor488 (Abcam, AB150153, 1:500), goat anti-rabbit Alexa Fluor488 (Invitrogen, A11008, 1:500), donkey anti-mouse Alexa Fluor555 (Abcam, AB150110, 1:500), donkey anti-rat Alexa Fluor555 (Abcam, AB150154, 1:500), donkey anti-mouse

Alexa Fluor647 (Abcam, AB150111, 1:500), donkey anti-rat Alexa Fluor647 (Abcam, AB150155, 1:500), goat anti-rabbit Alexa Fluor647 (Invitrogen, A21244, 1:500)

Validation

mouse anti-β-catenin (1:100, DSHB, N27A1): DSHB website states positive tested species reactivity and lists Immunohistochemistry under recommended applications.
at anti-E-cadherin (1:50, DSHB, DCAD2): DSHB website states positive tested species reactivity and lists immunohistochemistry under recommended applications.
rabbit anti-GFP (1:200, Thermo Fisher, G10362): Thermo Fisher website lists publications for tested reactivity and tested immunohistochemistry applications.
rat anti-RFP (1:20, gift from H. Leonhardt, Chromotek 5F8): Chromotek website states tested reactivity and tested immunohistochemistry application.
mouse anti-β-gal (1:1000, Promega Z378B): Promega website states tested reactivity and tested immunohistochemistry application.
mouse anti-Eya (1:100, DSHB eya10H6): DSHB website states positive tested species reactivity and lists Immunohistochemistry under recommended applications.
rabbit anti-pMad (1:200, abcam ab52903): Abcam website states tested predicted reactivity and tested immunohistochemistry application.

Animals and other research organisms

Policy information about studies involving animals; ARRIVE guidelines recommended for reporting animal research, and Sex and Gender in Research

Laboratory animals

This study worked with Drosophila melanogaster. Using females of age 2-5 days and male pupae.
Used fly lines (genotype, source):
w[118] , David Bilder
hsflp[1] , BDSC 6
hsflp[122] , Iswar Hariharan
act>y[+]>-GAL4,UAS-RFP/TM6c , BDSC 30558
act>y[+]>-GAL4,UAS-GFP/TM6b , Bruce A. Edgar
FRT42D ubi-eGFP/CyO , BDSC 5626
Act5C.GAL4 (FRT.CD2), UASp-UtrABD-eGFP /TM6c , BDSC 4780 & Katja Röper (recombined in this study)
tubGAL80[ts]-20; TM2/TM6b , BDSC 7019
Sco/CyO; tub-GAL80[ts]-7 , BDSC 7018
c306-GAL4 , BDSC 3743
tj-GAL4, Mef2-GAL80/CyO , Sally Horne-Badovinac
tj-GAL4, UAS-CD8tom/CyO; UAS-dcr2/TM6C , David Bilder
MTD-GAL4 (Otu-Gal4::VP16;nos-GAL4;nos-GAL4::VP16) , BDSC 31777
mirr-GAL4/TM3, Sb[1] , BDSC 29650
tub-GAL80[ts]-20/Cyo; fru-GAL4/TM6(hu) , Vincent Mirouse
matalpha-GAL-VP16 , BDSC 7063
GR1-Gal4 , BDSC 36287
PG150-GAL4/FM7a , Kim McCall
UASp-UtrABD-eGFP , Thomas Lecuit
UAS-upd1 / CyO,ubi-GFP , Martin Zeidler
UAS-Dl RNAi (GL000520) , BDSC 36784
UAS-hts-mCherry , BDSC 66171
UAS-eya , BDSC 5675
UAS-eya RNAi (HMS04515) , BDSC 57314
FRT42D shg [R69b]/ SM6b, cn#1 , Ulrich Tepass
UAS-shg RNAi (HMS00693) , BDSC 32904
UAS-shg RNAi (GL00646) , BDSC 38207
UAS-CadN RNAi (1093/GD) , VDRC1093
UAS-hpo , Barry Thompson
UAS-GFP (S56T) /CyO , BDSC 1521
UAS-mCD8::RFP , BDSC 32219
UAS-egfrλtop , BDSC 59843

Wild animals

This study did not involve wild animals.

Reporting on sex

As we studied mainly oogenesis, the findings of this study apply to females only.

Field-collected samples

This study did not involve samples collected in the field.

Ethics oversight

No ethical approval and guidance is necessary for research using Drosophila melanogaster.

Note that full information on the approval of the study protocol must also be provided in the manuscript.
